# Performance of image-based deep learning models for aortic dissection segmentation and diagnosis: a systematic review and meta-analysis

**DOI:** 10.3389/fcvm.2026.1734208

**Published:** 2026-04-14

**Authors:** Yichen Zhao, Yuhan Zhang, Jingyi Zhang, Caiying Chen, Ping Li, Ji Li, Qiang Fu

**Affiliations:** 1 The First Clinical Medical College, Heilongjiang University of Chinese Medicine, Harbin, China; 2 School of Basic Medicine, Heilongjiang University of Chinese Medicine, Harbin, China

**Keywords:** aortic dissection, deep learning, diagnosis, meta-analysis, segmentation, systematic review

## Abstract

**Objective:**

This meta-analysis was conducted to systematically evaluate the accuracy of image-based deep learning models for aortic dissection segmentation and diagnosis, aiming to provide an evidence base for developing intelligent detection tools.

**Methods:**

A comprehensive search was performed across the Cochrane Library, PubMed, Embase, and Web of Science to identify studies on the effectiveness of deep learning in aortic dissection segmentation or diagnosis up to November 3, 2024. Risk evaluation was carried out with the Quality Assessment of Diagnostic Accuracy Studies—2 (QUADAS-2).

**Results:**

A total of 48 studies on deep learning models for aortic dissection segmentation or diagnostic tasks were included, with 28 on segmentation tasks and 20 on diagnostic tasks. For segmentation tasks, the mean Dice coefficient was 89.2% ± 4.4% for false lumen segmentation, 90.8% ± 3.4% for true lumen segmentation, and 91.7% ± 6.1% for entire aorta segmentation. For diagnostic tasks, computed tomography (CT) based deep learning showed pooled sensitivity and specificity of 0.94 [95% confidence interval (CI): 0.89–0.96] and 0.92 (95% CI: 0.88–0.95), respectively. In terms of electrocardiogram-based deep learning, the pooled sensitivity and specificity were 0.85 (95% CI: 0.79–0.89) and 0.90 (95% CI: 0.87–0.92), respectively. Regarding the computed tomography angiography (CTA) based deep learning, the pooled sensitivity and specificity were 0.94 (95% CI: 0.90–0.96) and 0.95 (95% CI: 0.91–0.98), respectively. Additionally, some studies compared the diagnostic performance of deep learning with that of clinicians. The pooled sensitivity and specificity were 0.79 (95% CI: 0.65–0.89) and 0.95 (95% CI: 0.88–0.94), respectively.

**Conclusions:**

Image-based deep learning models demonstrated high accuracy for aortic dissection segmentation and diagnosis. They performed comparably to or better than clinicians. These findings support their potential as clinical assistive tools. Future work should prioritize multicenter validation, seamless integration of these models into clinical workflows, and enhancement of model generalizability to facilitate broader clinical adoption.

**Systematic Review Registration:**

https://www.crd.york.ac.uk/PROSPERO/view/CRD42024619403, identifier CRD42024619403.

## 1 Introduction

Aortic dissection (AD) is a critical cardiovascular disease. According to the Oxford Vascular Study, the annual incidence of AD is estimated to be six cases per 100,000 individuals (1). Currently, the Stanford classification is the prevailing standard for AD. Type A involves the ascending aorta, while Type B involves only the descending aorta. Type A is more dangerous, with an unoperated hospital mortality of 58% and a post-emergency mortality of 13% to 17% (1–3). Due to the strong link between the mortality of AD and the timeliness of rescue interventions, a critical issue for improving prognosis is to reduce the time window from symptom recognition to diagnosis.

Computed tomography (CT) and magnetic resonance imaging (MRI) angiography are the gold standards for diagnosing AD. Both techniques offer high resolution, minimal invasiveness, and accurate delineation of the location, extent, tear condition, and progression of AD (3). Nonetheless, AD diagnosis presents significant challenges. Radiologist expertise is required to identify intimal tears, differentiate between true (TL) and false lumen (FL) blood flow, and assess branch vessel involvement (1). Moreover, atypical cases may be overlooked. Therefore, a comprehensive evaluation using multimodal imaging is essential (3). Clinical physicians also face several challenges during the diagnostic process, including atypical symptoms, strong subjectivity in image interpretation, and time constraints.

Recent advances in artificial intelligence (AI), particularly deep learning (DL), have enabled the automated detection of complex lesions, transforming medical image analysis. In cardiovascular imaging, DL models show great potential for overcoming diagnostic challenges, such as interobserver variability and the need for timely interpretation (4). Using a computational medicine framework to approach AD diagnosis is consistent with the priority of the emerging digital health framework for scalable, real-time decision support systems (5).

A DL model enables automated analysis. It rapidly identifies dissection features in images (e.g., the intimal flap and TLs/FLs). This reduces human error and substantially alleviates clinicians' workload. The diagnostic accuracy of the model is not affected by an increased workload. Moreover, it can provide early warnings and analyze atypical cases (e.g., intramural hematomas) to assist in clinical decision-making. This compensates the clinician's shortcomings (1, 3). In contrast to traditional machine learning (ML), DL excels at image processing. Unlike DL, ML relies on handcrafted variables for model building and manual extraction of morphological features (such as shape and texture) from images (6). DL models can autonomously process image data, achieving segmentation and automatic classification of pathological regions (7) and outperforming ML models.

Some investigators have explored DL models for the segmentation, diagnosis, and classification of AD. However, no systematic evidence confirms the accuracy of these models. This presents challenges in developing intelligent detection tools for AD. Hence, this study was conducted to systematically examine the effectiveness of DL models for AD segmentation, diagnosis, and classification. The findings will establish an evidence-based foundation for developing these tools.

Although many studies have examined DL-based AD segmentation and diagnostic models, the evidence is still scattered. Comprehensive systematic reviews and meta-analyses that rigorously evaluate and quantify the integrated performance of these models are currently lacking. This gap leads to limitations in crucial areas. First, dataset size, model architecture, validation methods, and performance reporting metrics vary across individual studies. Consequently, it is challenging for clinical practitioners and technology developers to derive clear, comparable conclusions about the efficacy of different models (8). Second, the absence of high-level evidence hinders the critical step of translating these promising DL tools from research settings to clinical practice and impedes the development of relevant clinical application guidelines (9). The current rigorous systematic review and meta-analysis aim to address these limitations by providing a comprehensive, evidence-based, quantitative assessment of the accuracy of image-based DL models in AD segmentation and diagnosis.

## 2 Methods

### 2.1 Study registration

This meta-analysis was conducted and reported per the Preferred Reporting Items for Systematic Reviews and Meta-Analyses 2020 (PRISMA-2020) guidelines and was prospectively registered in the International Prospective Register of Systematic Reviews (CRD42024619403).

### 2.2 Eligibility criteria

**Inclusion criteria:**

(i) Studies that comprehensively developed DL models for AD image segmentation;
(ii) Studies that fully constructed DL models for AD diagnosis;
(iii) Studies with case-control, cohort, or cross-sectional designs;
(iv) English studies.

**Exclusion criteria:**

**The following studies were excluded:**

(i) Meta-analyses, reviews, guidelines, expert opinions, or unpublished conference abstracts;
(ii) Studies that leveraged non-imaging data (e.g., clinical features or laboratory results) to develop DL models for AD segmentation or diagnosis;
(iii) Studies that failed to report at least one standard performance metric, including c-index, sensitivity (Sen), specificity (Spe), accuracy, precision, confusion matrix, F1 score, calibration curve, or decision curve.

### 2.3 Data sources and search strategy

A search was conducted across the Cochrane Library, PubMed, Embase, and Web of Science up to November 3, 2024. A combination of subject headings and free-text terms was used for the search. No restrictions on publication year or region were imposed. Details are presented in Supplementary Table S1.

### 2.4 Study selection and data extraction

The retrieved records were imported into EndNote to eliminate duplicates. Then, a title or abstract review was conducted to select potentially relevant articles. Next, the full texts of the selected articles were downloaded and reviewed to determine final inclusion.

Prior to data extraction, a standardized data extraction spreadsheet was developed. The extracted data included the following: title, DOI, first author, publication year, country, study type, patient source, task type (segmentation or diagnosis), image source, segmentation method, diagnostic criteria for AD, AD case numbers, total case numbers, number of AD cases and total cases in the training set, number of AD cases and total cases in the validation set, validation set generation method, model type, and comparison with clinician's diagnosis.

These processes were executed independently by two investigators, followed by a cross-check to ensure consistency. Any discrepancies were addressed through consultation with an additional investigator.

### 2.5 Risk of bias evaluation

The Quality Assessment of Diagnostic Accuracy Studies-2 (QUADAS-2) tool was leveraged to appraise the risk of bias (ROB) and clinical applicability of the included articles (10). The tool involves four domains: patient selection, index test, reference standard, and flow and timing. Each domain comprises several signaling questions with the following response options: yes, no, or unclear. These responses correspond to a rating of low, high, or unclear ROB, respectively. A domain was rated as low ROB if all of its signaling questions were answered with yes. A rating of potential ROB was made if any signaling questions were answered with no. In this context, investigators used the provided guidelines to determine the ROB. An unclear rating was assigned when an article did not provide sufficient information to make a judgment.

These processes were independently executed by two investigators (YH-Z and JY-Z), followed by a cross-check to ensure consistency. The two investigators' evaluations showed high inter-rater reliability (Kappa = 0.861). Any discrepancies were addressed through consultation with an additional investigator (Q-F OR YC-Z).

### 2.6 Handling of potential dataset overlapping

To address potential dataset overlap across the included studies—a situation that can introduce bias into pooled estimates—the following mitigation measures were implemented. First, the origins of each study were meticulously reviewed. Each study was examined for reported author affiliations, patient recruitment periods, and data sources (e.g., specific hospital names and public database identifiers). Studies originating from the same institution with overlapping recruitment timelines were flagged for further scrutiny. Second, the cohort characteristics were compared. For studies suspected of potential overlap (e.g., those from the same research group or institution), we compared the reported patient demographics (e.g., age and sex), sample sizes, and image acquisition protocols. In cases of highly similar or identical cohorts, only the study with the most comprehensive data or the largest sample size was retained for the meta-analysis to prevent duplication.

### 2.7 Outcome measures

For segmentation tasks, the primary outcome measures were the Dice coefficient and the Dice similarity coefficient. For diagnostic tasks, the main outcome measures were indicators reflecting the accuracy of DL models, including Sen, Spe, positive likelihood ratio (PLR), negative likelihood ratio (NLR), diagnostic odds ratio (DOR), and the area under the summary receiver operating characteristic (SROC) curve (AUSROC).

### 2.8 Synthesis methods

Data analyses were performed using Stata 15.0 (StataCorp LLC, College Station, TX). The pooled effect sizes for Sen, Spe, PLR, NLR, and DOR, along with their corresponding 95% confidence intervals (CIs), were computed. The heterogeneity parameter (*I*^2^) was extracted. Furthermore, the AUSROC was estimated. Deeks' funnel plots were leveraged to evaluate publication bias. The clinical applicability was appraised through Fagan's nomogram. In certain studies where the 2 × 2 contingency table was not directly provided, the table was derived from Sen, Spe, precision, accuracy, and the number of cases. Furthermore, this meta-analysis was conducted exclusively on validation or training sets. Subgroup analyses were executed based on image type, validation set generation method, and the Stanford classification of AD.

However, some studies on segmentation tasks provided the Jaccard index. The Dice coefficient was converted utilizing a formula proposed by Muhammad Ibrahim Khalil et al. (11) (Equation 1). A meta-analysis of the Dice coefficient was not conducted. Instead, only the mean and standard deviation (SD) were calculated because standard errors (SEs) or CIs were unavailable for each original article. Results were deemed statistically significant at *P* < 0.05.

$$\mathrm{Dice}\;(\text{\%})=\frac{2*\;\mathrm{Jaccard}}{1+\mathrm{Jaccard}}$$ (1)

## 3 Results

### 3.1 Study selection

**Figure 1:**
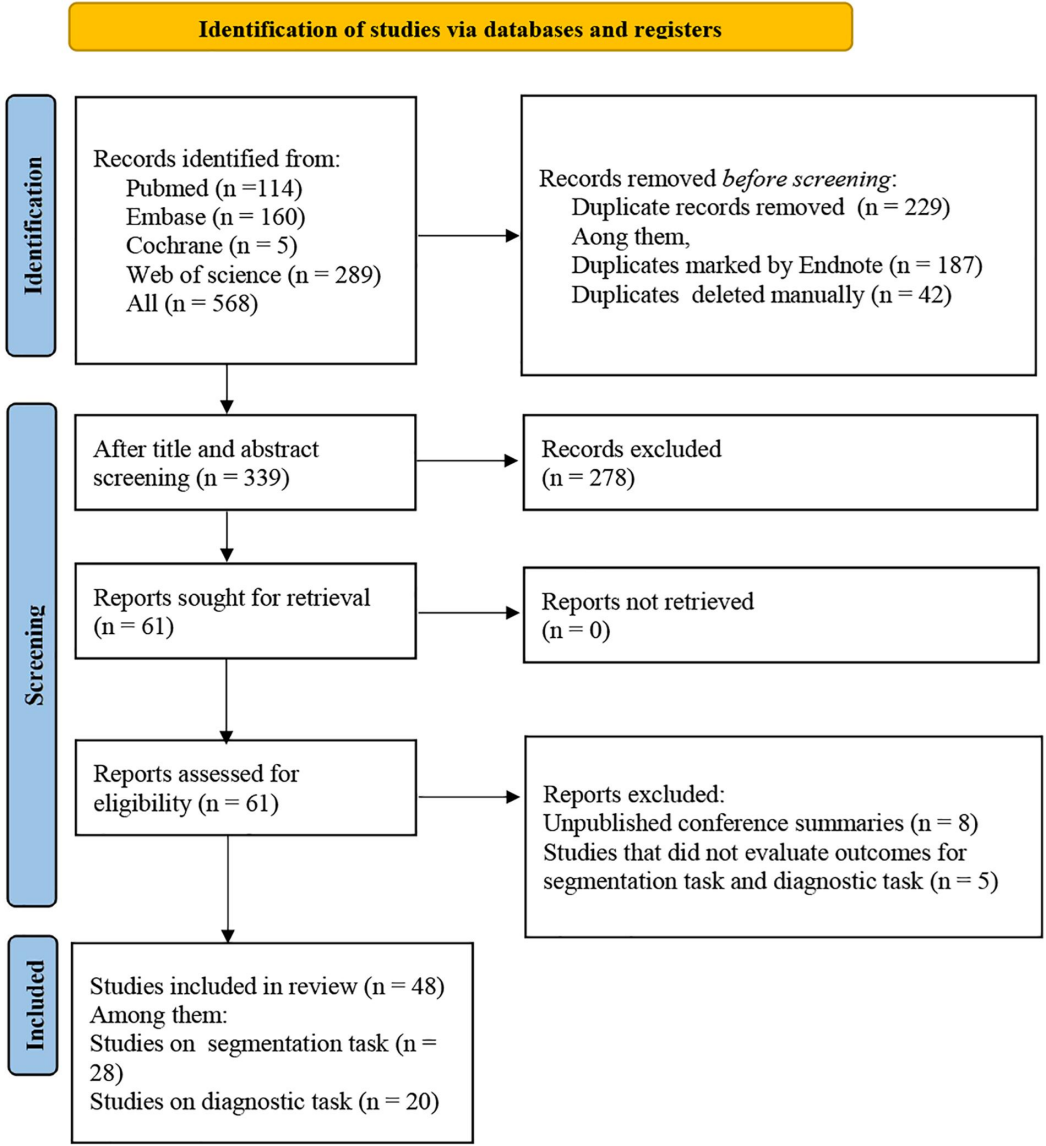
PRISMA flowchart.

A total of 568 articles were retrieved from the databases, 187 duplicates were automatically identified by EndNote, and an additional 42 duplicates were marked manually. After excluding 229 articles, the review focused on the titles and abstracts of the remaining 339 articles. Then, 278 irrelevant articles were removed. Finally, the full texts of the remaining 61 articles were downloaded for further reading. Eight unpublished conference abstracts and five articles that did not evaluate outcome indicators for segmentation tasks and diagnostic tasks were deleted. Ultimately, 48 articles were included, including 28 on segmentation tasks and 28 on diagnostic tasks (Figure 1).

### 3.2 Study characteristics

DL models for AD segmentation tasks were constructed in 28 articles, with publication years ranging from 2019 to 2024. These articles involved 1,810 AD patients, with 9,488 images. The articles were from seven countries: 17 from China, five from the USA, two from Korea, and one each from Finland, France, Greece, and Italy. Of the articles, 19 were single-center, six were multi-center, one was derived from a registry database, and two did not explicitly describe the patient source. Regarding image source, 13 articles focused on computed tomography angiography (CTA) (one of which also included MRI), 13 articles were based on CT, one article relied on digital subtraction angiography, and one article incorporated both CTA and CT. In terms of the validation set generation method, 15 articles were randomly sampled, and 11 underwent cross-validation (three with 3-fold cross-validation, four with 4-fold cross-validation, two with 5-fold cross-validation, and two with 6-fold cross-validation). Additionally, two articles did not specify the method Supplementary Table S2) (2, 12–38).

DL models for AD diagnostic tasks were created in 20 articles, with publication years ranging from 2019 to 2024. A total of 29,388 patients (with 92,319 images) were included, of whom 2,560 had AD. The articles were all case-control designs, encompassing six countries. Among them, 13 articles originated from China, three from Japan, and one each from France, Germany, Korea, and the USA. The patient sources included nine studies from a single center, eight studies from multiple centers, and three studies where the patient source was not clearly stated. In terms of image segmentation methods, six articles employed automatic segmentation, five utilized manual segmentation, and nine did not specify the method. Regarding imaging sources, eight articles utilized CTA, six employed CT (including one study that involved both CT and CTA), three used electrocardiograms (ECGs) (including one study that included both ECGs and chest x-rays), two focused on non-contrast enhanced CT (NCE-CT), and one addressed x-ray. Nine articles generated the validation set via random sampling, nine others utilized 5-fold cross-validation, and two did not provide information on validation set generation. Among them, four articles with patients from multiple centers performed external validation at other medical institutions Supplementary Table S3) (18, 39–57).

### 3.3 Quality evaluation

**Figure 2:**
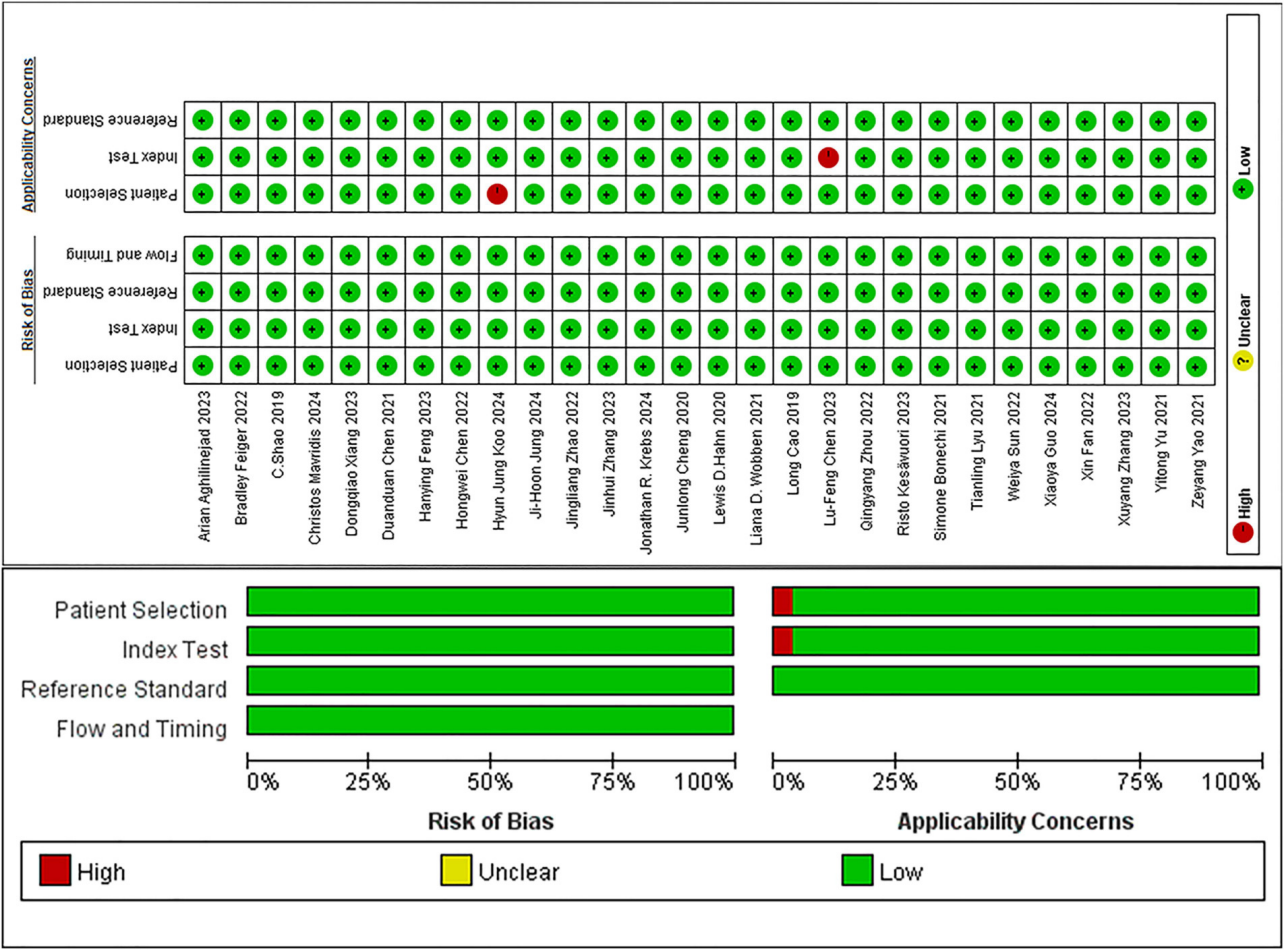
Quality evaluation for articles on segmentation tasks.

In studies on segmentation tasks, all employed continuous or random case sequences, avoiding case-control designs and inappropriate exclusions. Therefore, the selection of cases in these studies had a low ROB. The included articles employed supervised ML, conducted with a certain degree of awareness regarding the gold standard. Unlike humans, machine interpretation did not exhibit bias due to knowledge of the gold standard. DL models operated based on established rules for identifying positive events and were trained accordingly, meaning that the threshold could be considered pre-determined. Thus, it was posited that the implementation or interpretation of the included articles did not introduce notable bias. The interpretation of the gold standard could well differentiate the status of the target disease, and therefore, its implementation and interpretation of the gold standard were unlikely to introduce significant bias. The included studies ensured an appropriate time interval between image acquisition and the gold standard. Furthermore, all cases were incorporated into the analysis, and every patient underwent the same gold standard procedure. Therefore, it was concluded that the case flow does not introduce significant bias. In the evaluation of clinical practicality, one study included healthy participants, resulting in a mismatch with the evaluation criteria, and was assessed as having a high ROB. The remaining studies included patients whose backgrounds were consistent with the evaluation criteria, resulting in a low ROB. One study was unable to extract the expected evaluation parameters, and its implementation and interpretation were not aligned with the evaluation issues, resulting in a high ROB. The implementation and interpretation of the remaining studies aligned well with the evaluation issues, resulting in a low ROB. In summary, 26 of the 28 studies on segmentation tasks exhibited a low ROB, while two demonstrated a high ROB (Figure 2).

**Figure 3:**
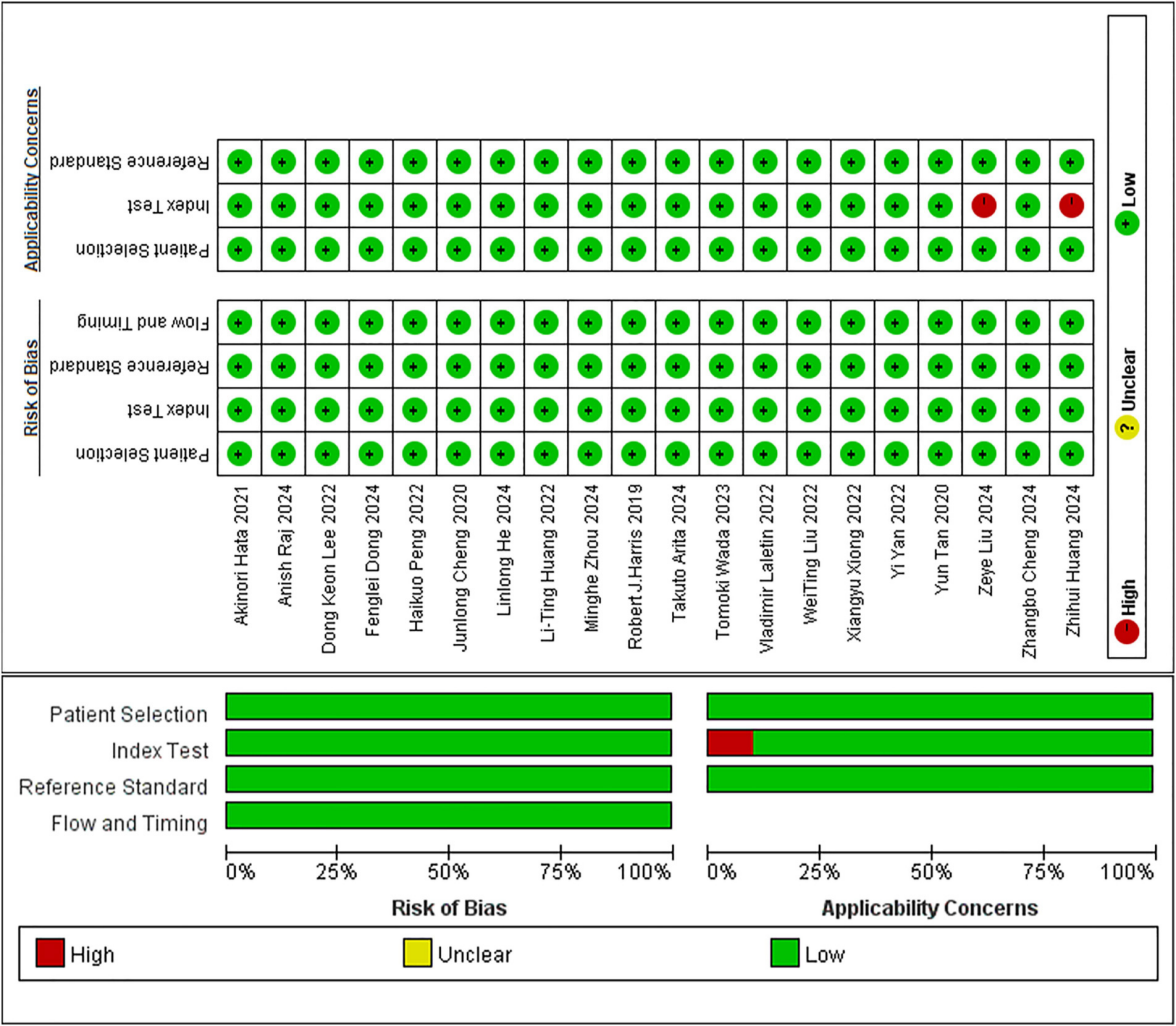
Quality evaluation for articles on diagnostic tasks.

In studies on diagnostic tasks, although all employed case-control designs, the interpretation rules were unaffected by knowledge of the study type because machine interpretation rules differed from human judgment. Moreover, the included studies avoided inappropriate exclusions, resulting in a low ROB in case selection. All studies were supervised ML tasks, which did not introduce bias from knowing the gold standard. Therefore, the ROB for the implementation or interpretation of the evaluated studies was low. The gold standard used effectively distinguished the status of target diseases, thereby minimizing the potential for bias in its implementation and interpretation. The included studies ensured an appropriate time interval between image acquisition and the gold standard. Furthermore, all cases were incorporated into the analysis, and each patient underwent the same gold standard procedure. Thus, it was concluded that the case flow did not introduce significant bias. In terms of clinical applicability, two studies were unable to extract valid information for the 2 × 2 contingency table. The implementation and interpretation of these studies did not align with the evaluation questions, resulting in a high ROB. In contrast, the remaining studies demonstrated a better alignment between their implementation and interpretation with the evaluation questions, thus presenting a low ROB. In summary, 18 of the 20 studies on diagnostic tasks exhibited a low ROB, while two demonstrated a high ROB (Figure 3).

### 3.4 Accuracy analysis of the segmentation task

The Dice coefficient of DL for AD segmentation was extracted to appraise its accuracy. Some articles used the Jaccard coefficient, from which the Dice coefficient was derived. However, some studies did not report the SD or SE of the Dice coefficient, making quantitative analysis impossible and limiting calculations to averages only. The analysis revealed that 23 studies reported the accuracy of entire aortic segmentation, with a mean Dice coefficient of 91.7 (SD: 6.1). Among these, the images from nine studies were sourced from CTA, with a mean Dice coefficient of 91.9 (SD: 5.8). In 14 studies, the source of images was CT, and the mean Dice coefficient was 91.4 (SD: 6.9). Eleven studies reported on the accuracy of FL segmentation in AD, with a mean Dice coefficient of 89.2 (SD: 4.4). Out of these, the images of seven studies were derived from CTA, with a mean Dice coefficient of 88.5 (SD: 4.8). In four studies, the image source was CT, along with a mean Dice coefficient of 91.5 (SD: 2.7). Thirteen studies reported the accuracy of TL segmentation in AD, and the mean Dice coefficient was 90.8 (SD: 3.4). In these studies, the images of nine studies were CTA, and the mean Dice coefficient was 89.5 (SD: 2.5). In four studies, the images were from CT, with a mean Dice coefficient of 93.7 (SD: 3.7) (Table 1). Some studies also focused on the Dice coefficient for branch vessels, TL thrombus, and FL thrombus separately. Additionally, a few studies investigated the axial, coronal, and sagittal planes individually. However, these studies were limited. Hence, their Dice coefficients were not pooled.

**Table 1:** Mean dice coefficients.

Segmentation task	Image	*n*	Mean (SD)	Range
FL	CT	4	91.5 (2.7)	87.1–92.2
	CTA	7	88.5 (4.8)	77.0–93.2
	Overall	11	89.2 (4.4)	77.0–93.2
TL	CT	4	93.7 (3.7)	88.4–96.2
	CTA	9	89.5 (2.5)	86.0–93.0
	Overall	13	90.8 (3.4)	86.0–96.2
Entire Aorta	CT	9	91.4 (6.9)	75.4–96.9
	CTA	14	91.9 (5.8)	73.0–96.0
	Overall	23	91.7 (6.1)	73.0–96.9

Notably, the current meta-analysis encountered a significant methodological challenge in quantifying segmentation task performance. Due to the absence of SD, SE, or CI values for Dice coefficients in most of the eligible studies, a conventional meta-analysis to calculate pooled effect sizes and CIs could not be performed. Thus, the reported mean and SD of Dice coefficients were descriptive statistics aimed at providing a narrative summary of the accuracy reported across investigations. However, these results are subject to potential heterogeneity due to the inability to weight studies, requiring cautious interpretation. This limitation prevented a statistical evaluation of the robustness and consistency of the segmentation performance of DL models.

### 3.5 Meta-analysis for articles on diagnostic tasks

**Figure 4:**
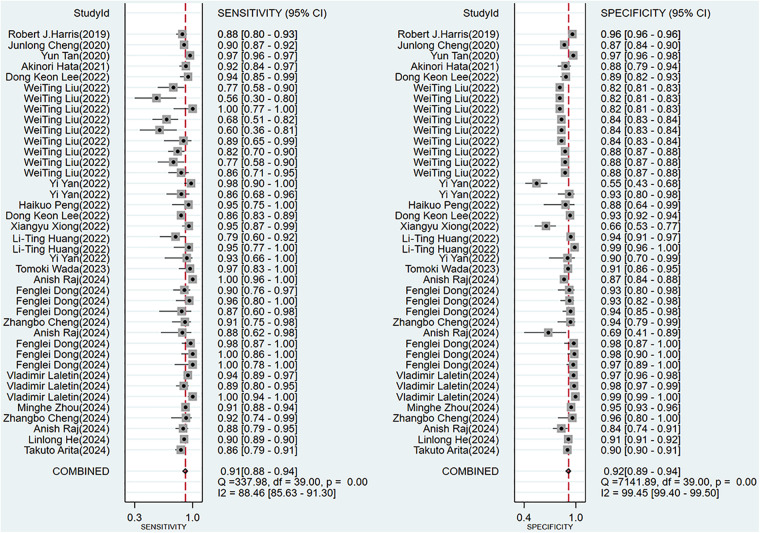
Forest plot for sensitivity and specificity of DL.

**Figure 5:**
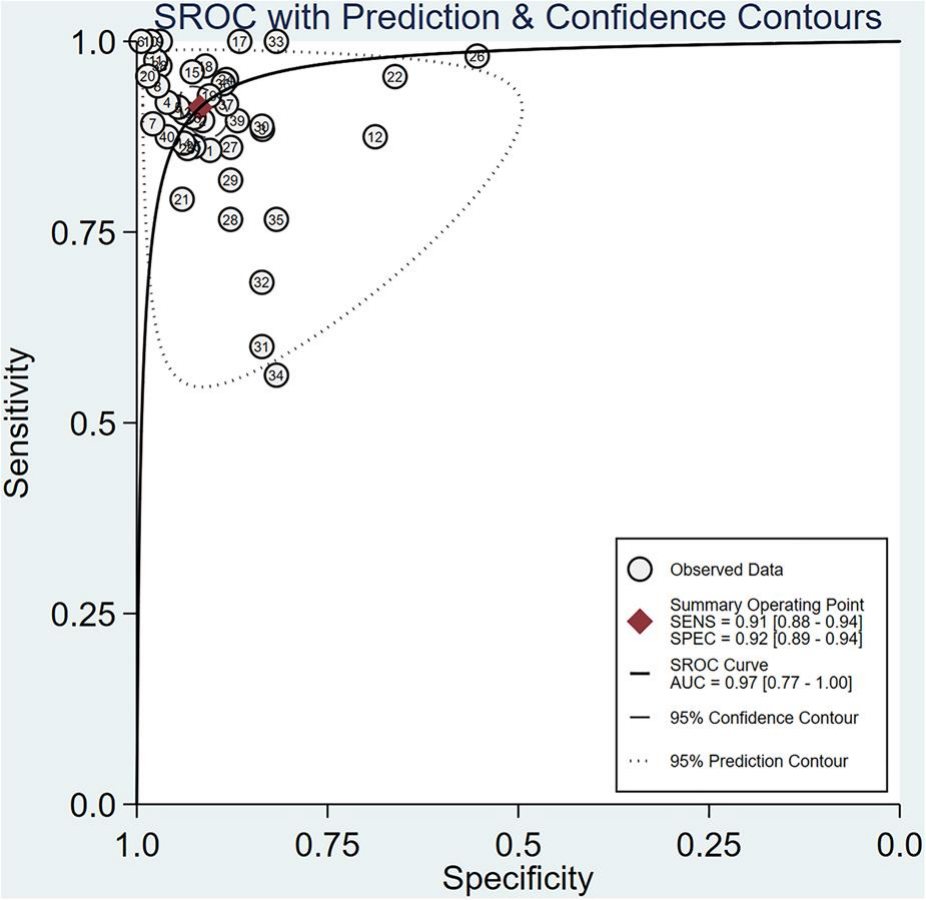
SROC curve for sensitivity and specificity of DL.

**Figure 6:**
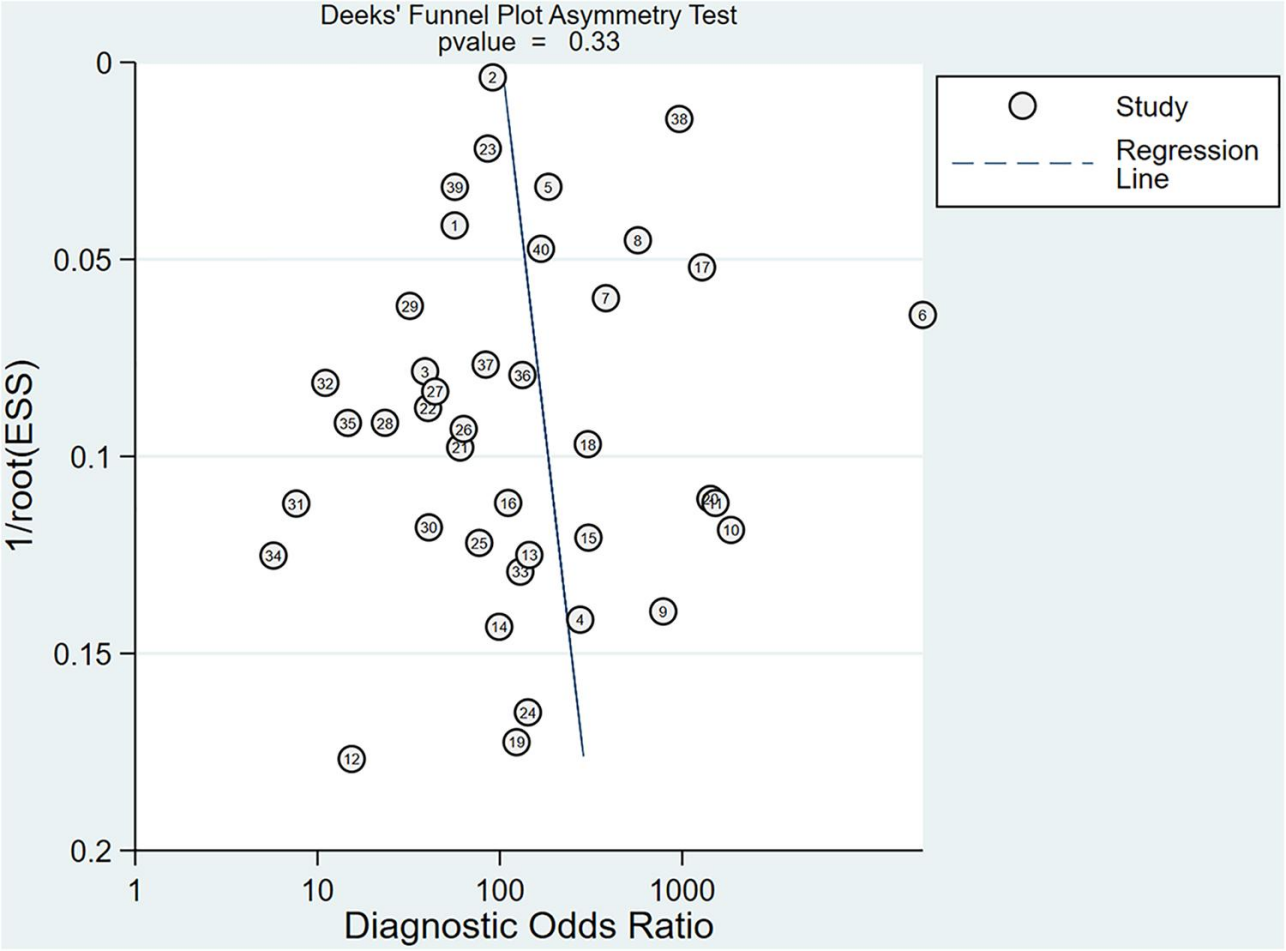
Deek's funnel plot for sensitivity and specificity of DL.

**Figure 7:**
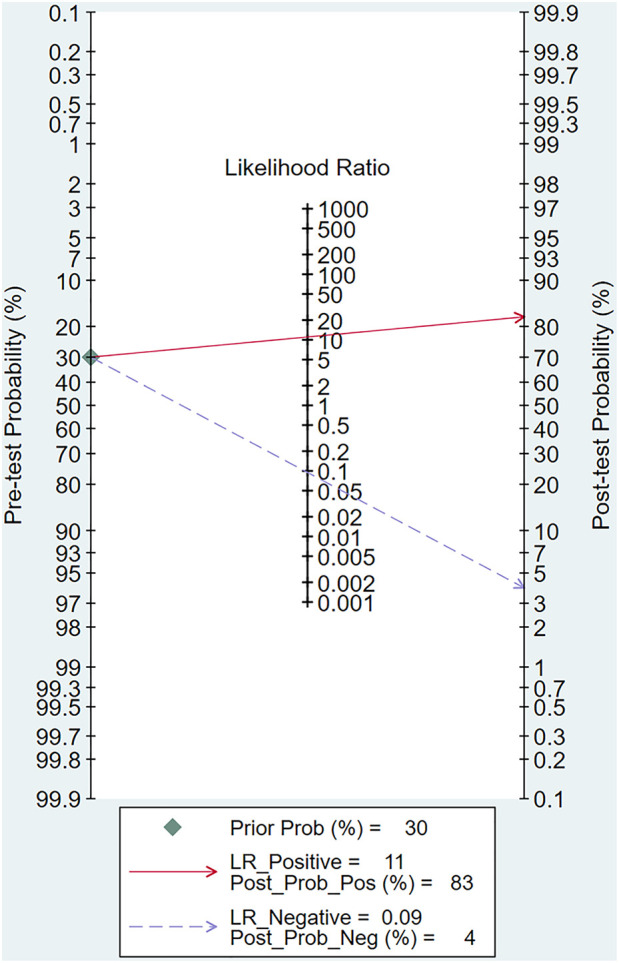
Fagan's nomogram for sensitivity and specificity of DL.

To provide an overview of the diagnostic capabilities of DL models, we first pooled data from all available studies. Of the 20 studies included, forty 2 × 2 contingency tables reported the effectiveness of DL-based models in diagnosing AD. The meta-analysis revealed that the Sen, Spe, PLR, NLR, DOR, and AUSROC were 0.91 (95% CI: 0.88–0.94), 0.92 (95% CI: 0.89–0.94), 11.1 (95% CI: 8.2–14.9), 0.09 (95% CI: 0.07–0.13), 117 (95% CI: 70–196), and 0.97 (95% CI: 0.77–1.00), respectively (Table 2, Figures 4, 5). According to Deek's funnel plot, no significant publication bias was observed (*P* = 0.33) (Figure 6). Given a prior probability of 0.3, when the DL model predicted AD, the probability that the case was truly AD was 0.83 (Figure 7).

**Table 2:** Subgroup analysis results.

Subgroup	*n*	Sen (95% CI)	Spe (95% CI)	PLR (95% CI)	NLR (95% CI)	DOR (95% CI)	AUSROC (95% CI)	Heterogeneity (*I*^2^)
Validation set generation method								
Cross-validation	9	0.90 (0.88, 0.92)	0.91 (0.85, 0.94)	9.9 (6.2, 15.7)	0.11 (0.10, 0.13)	88 (64, 120)	0.95 (0.74–0.99)	0.94
Random sampling	26	0.91 (0.87, 0.94)	0.92 (0.88, 0.95)	11.4 (7.5, 17.3)	0.10 (0.06, 0.15)	117 (55, 251)	0.97 (0.66–1.00)	0.95
External validation	5	0.95 (0.86, 0.98)	0.91 (0.86, 0.95)	11.0 (6.9, 17.3)	0.05 (0.02, 0.16)	210 (70, 627)	0.96 (0.64–1.00)	0.77
Image type								
CTA	11	0.94 (0.90, 0.96)	0.95 (0.91, 0.98)	20.2 (9.8, 41.8)	0.06 (0.04, 0.10)	323 (119, 881)	0.98 (0.96–0.99)	0.93
CT	13	0.94 (0.89, 0.96)	0.92 (0.88, 0.95)	11.5 (7.7, 17.4)	0.07 (0.04, 0.12)	172 (79, 371)	0.98 (0.80–1.00)	0.90
ECGs	5	0.85 (0.79, 0.89)	0.90 (0.87, 0.92)	8.4 (6.2, 11.4)	0.17 (0.12, 0.24)	51 (27, 96)	0.94 (0.73–0.99)	1.00
Others	11	0.86 (0.77, 0.92)	0.85 (0.81, 0.89)	5.8 (4.4, 7.6)	0.16 (0.10, 0.28)	35 (18, 71)	0.91 (0.59–0.99)	0.92
AD type								
A	8	0.95 (0.89, 0.98)	0.95 (0.88, 0.98)	17.5 (7.3, 41.9)	0.05 (0.02, 0.13)	356 (66, 1,914)	0.99 (0.77–1.00)	1.00
B	7	0.83 (0.68, 0.91)	0.94 (0.87, 0.97)	12.9 (5.5, 30.3)	0.18 (0.09, 0.37)	70 (15, 319)	0.95 (0.72–0.99)	1.00
Untyped	25	0.91 (0.88, 0.94)	0.90 (0.87, 0.93)	9.2 (6.7, 12.6)	0.10 (0.07, 0.13)	96 (59, 156)	0.96 (0.76–1.00)	0.97
Image segmentation type								
Automatic segmentation	10	0.93 (0.88, 0.97)	0.89 (0.80, 0.94)	8.3 (4.6, 14.9)	0.07 (0.04, 0.14)	113 (49, 258)	0.97 (0.79–1.00)	0.93
Manual segmentation	14	0.94 (0.90, 0.96)	0.96 (0.94, 0.98)	26.1 (16.1, 42.3)	0.06 (0.04, 0.10)	415 (189, 914)	0.98 (0.68–1.00)	0.69
Overall	**40**	**0.91** (**0.88, 0.94)**	**0.92** (**0.89, 0.94)**	**11.1** (**8.2, 14.9)**	**0.09** (**0.07, 0.13)**	**117** (**70, 196)**	**0.97** (**0.77–1.00)**	**0**.**98**

The bold values in the last row represent the overall (pooled) performance estimates derived from meta-analysis of all included studies/subgroups.

### 3.6 Subgroup analysis

The following sections present multiple meta-analyses on the diagnostic performance of DL models. Due to the large number of analyses, the most comprehensive numerical comparison of all pooled estimates is provided in Table 2. This section focuses on interpreting the key findings and their clinical implications.

#### 3.6.1 Subgroup analysis based on validation set generation methods

Subgroup analysis by validation method is crucial for assessing the risk of overfitting and the true generalizability of models. Performance on a single random split of the development data (random sampling) may be optimistically biased; cross-validation provides a more robust internal estimate by leveraging multiple splits. However, external validation on independent datasets remains the gold standard for evaluating real-world clinical applicability.

##### 3.6.1.1 Cross-validation

In studies using cross-validation to generate validation sets, nine 2 × 2 contingency tables reported the effectiveness of DL-based models in diagnosing AD. The meta-analysis showed that the Sen, Spe, PLR, NLR, DOR, and AUSROC were 0.90 (95% CI: 0.88–0.92), 0.91 (95% CI: 0.85–0.94), 9.9 (95% CI: 6.2–15.7), 0.11 (95% CI: 0.10–0.13), 88 (95% CI: 64–120), and 0.95 (95% CI: 0.74–0.99), respectively (Table 2). According to Deek's funnel plot, no significant publication bias was observed (*P* = 0.73). Assuming that the prior probability was 0.3, the probability of true AD was 0.81 when the DL result indicated AD (Supplementary Figures S1–S4).

##### 3.6.1.2 Random sampling

In studies using random sampling to generate validation sets, twenty-six 2 × 2 contingency tables reported the effectiveness of the DL-based model for diagnosing AD. The meta-analysis revealed that the Sen, Spe, PLR, NLR, DOR, and AUSROC were 0.91 (95% CI: 0.87–0.94), 0.92 (95% CI: 0.88–0.95), 11.4 (95% CI: 7.5–17.3), 0.10 (95% CI: 0.06–0.15), 117 (95% CI: 55–251), and 0.97 (95% CI: 0.66–1.00), respectively (Table 2). Based on Deek's funnel plot, a potential publication bias was observed (*P* = 0.01). Given a prior probability of 0.3, when the DL model predicted AD, the probability that the case was truly AD was 0.83 (Supplementary Figures S5–S8).

##### 3.6.1.3 External validation

In studies using external validation, five 2 × 2 contingency tables reported the effectiveness of DL-based models in diagnosing AD. The meta-analysis showed that the Sen, Spe, PLR, NLR, DOR, and AUSROC were 0.95 (95% CI: 0.86–0.98), 0.91 (95% CI: 0.86–0.95), 11.0 (95% CI: 6.9–17.3), 0.05 (95% CI: 0.02–0.16), 210 (95% CI: 70–627), and 0.96 (95% CI: 0.64–1.00), respectively (Table 2). According to Deek's funnel plot, a potential publication bias was observed (*P* = 0.01). Assuming that the prior probability was 0.3, the probability of true AD was 0.82 when the DL result indicated AD (Supplementary Figures S9–S12).

#### 3.6.2 Subgroup analysis based on image types

Stratifying results by imaging modality is essential for evaluating clinical applicability, as the choice between definitive (e.g., CTA) and screening (e.g., ECG) tools involves a trade-off between accuracy and practicality.

##### 3.6.2.1 CTA

In studies using CTA images for diagnosis, eleven 2 × 2 contingency tables reported the effectiveness of the DL-based model in diagnosing AD. The meta-analysis showed that the Sen, Spe, PLR, NLR, DOR, and AUSROC were 0.94 (95% CI: 0.90–0.96), 0.95 (95% CI: 0.91–0.98), 20.2 (95% CI: 9.8–41.8), 0.06 (95% CI: 0.04–0.10), 323 (95% CI: 119–881), and 0.98 (95% CI: 0.96–0.99), respectively (Table 2). Deek's funnel plot indicated no significant publication bias (*P* = 0.13). Assuming that the prior probability was 0.3, and when the DL result was AD, the probability that it was truly AD was 0.90 (Supplementary Figures S13–S16).

##### 3.6.2.2 CT

In studies using CT images for diagnosis, thirteen 2 × 2 contingency tables reported the effectiveness of the DL-based model in diagnosing AD. The meta-analysis demonstrated that the Sen, Spe, PLR, NLR, DOR, and AUSROC were 0.94 (95% CI: 0.89–0.96), 0.92 (95% CI: 0.88–0.95), 11.5 (95% CI: 7.7–17.4), 0.07 (95% CI: 0.04–0.12), 172 (95% CI: 79–371), and 0.98 (95% CI: 0.80–1.00), respectively (Table 2). Deek's funnel plot revealed no significant publication bias (*P* = 0.36). Assuming that the prior probability was 0.3, the probability of true AD was 0.83 when the DL result indicated AD (Supplementary Figures S17–S20).

##### 3.6.2.3 ECGs

In studies using ECG images for diagnosis, five 2 × 2 contingency tables reported the effectiveness of the DL-based model in diagnosing AD. The meta-analysis revealed that the Sen, Spe, PLR, NLR, DOR, and AUSROC were 0.85 (95% CI: 0.79–0.89), 0.90 (95% CI: 0.87–0.92), 8.4 (95% CI: 6.2–11.4), 0.17 (95% CI: 0.12–0.24), 51 (95% CI: 27–96), and 0.94 (95% CI: 0.73–0.99), respectively (Table 2). Deek's funnel plot showed no significant publication bias (*P* = 0.08). Given a prior probability of 0.3, when the DL model predicted AD, the probability that the case was truly AD was 0.78 (Supplementary Figures S21–S24).

##### 3.6.2.4 Other images

In studies using other images for diagnosis, eleven 2 × 2 contingency tables reported the effectiveness of the DL-based model in diagnosing AD. The meta-analysis demonstrated that the Sen, Spe, PLR, NLR, DOR, and AUSROC were 0.86 (95% CI: 0.77–0.92), 0.85 (95% CI: 0.81–0.89), 5.8 (95% CI: 4.4–7.6), 0.16 (95% CI: 0.10–0.28), 35 (95% CI: 18–71), and 0.91 (95% CI: 0.59–0.99), respectively (Table 2). Deek's funnel plot revealed no significant publication bias (*P* = 0.16). Assuming that the prior probability was 0.3, the probability of true AD was 0.71 when the DL result indicated AD (Supplementary Figures S25–S28).

#### 3.6.3 Subgroup analysis based on classification of aortic dissection

Analyzing performance by dissection type (A vs. B) addresses a key clinical need, as the diagnostic priority and morphological challenges differ significantly between these entities, potentially impacting model accuracy.

##### 3.6.3.1 Type-A AD

In studies focusing on Type-A AD, eight 2 × 2 contingency tables reported the effectiveness of the DL-based model in diagnosing AD. The meta-analysis indicated that the Sen, Spe, PLR, NLR, DOR, and AUSROC were 0.95 (95% CI: 0.89–0.98), 0.95 (95% CI: 0.88–0.98), 17.5 (95% CI: 7.3–41.9), 0.05 (95% CI: 0.02–0.13), 356 (95% CI: 66–1,914), and 0.99 (95% CI: 0.77–1.00), respectively (Table 2). Deek's funnel plot revealed no publication bias (*P* = 0.11). Given a prior probability of 0.3, when the DL model predicted AD, the probability that the case was truly AD was 0.88 (Supplementary Figures S29–S32).

##### 3.6.3.2 Type-B AD

In studies focusing on Type-B AD, seven 2 × 2 contingency tables reported the effectiveness of the DL-based model in diagnosing AD. The meta-analysis showed that the Sen, Spe, PLR, NLR, DOR, and AUSROC were 0.83 (95% CI: 0.68–0.91), 0.94 (95% CI: 0.87–0.97), 12.9 (95% CI: 5.5–30.3), 0.18 (95% CI: 0.09–0.37), 70 (95% CI: 15–319), and 0.95 (95% CI: 0.72–0.99), respectively (Table 2). Deek's funnel plot indicated no significant publication bias (*P* = 0.36). Assuming that the prior probability was 0.3, and when the DL result was AD, the probability that it was truly AD was 0.85 (Supplementary Figures S33–S36).

#### 3.6.4 Subgroup analysis based on image segmentation type

Investigating the influence of the segmentation method (manual vs. automatic) can help clarify whether diagnostic accuracy depends on the quality of prior image processing. This could be a potential bottleneck in fully automated pipelines.

##### 3.6.4.1 Automatic segmentation

In investigations using automatically segmented images for diagnostic tasks, ten 2 × 2 contingency tables reported the effectiveness of decision tree models in AD diagnosis. The meta-analysis demonstrated the Sen of 0.93 (95% CI: 0.88–0.97), Spe of 0.89 (95% CI: 0.80–0.94), PLR of 8.3 (95% CI: 4.6–14.9), NLR of 0.07 (95% CI: 0.04–0.14), DOR of 113 (95% CI: 49–258), and AUSROC of 0.97 (95% CI: 0.79–1.00) (Table 2). Deek's funnel plots indicated no significant publication bias (*P* = 0.14). Assuming that the prior probability was 0.3, and the decision tree result for AD was positive, the probability of true AD was 0.78 (Supplementary Figures S37–S40).

##### 3.6.4.2 Manual segmentation

In studies using manually segmented images for diagnostic tasks, fourteen 2 × 2 contingency tables reported the effectiveness of decision tree models in AD diagnosis. The meta-analysis showed the Sen of 0.94 (95% CI: 0.90–0.96), Spe of 0.96 (95% CI: 0.94–0.98), PLR of 26.1 (95% CI: 16.1–42.3), NLR of 0.06 (95% CI: 0.04–0.10), DOR of 415 (95% CI: 189–914), and AUSROC of 0.98 (95% CI: 0.68–1.00) (Table 2). Deek's funnel plots revealed no significant publication bias (*P* = 0.80). Given a prior probability of 0.3, when the decision tree predicted AD, the probability that the case was truly AD was 0.92 (Supplementary Figures S41–S44).

### 3.7 Diagnostic accuracy of deep learning vs. clinicians

A direct comparison with clinicians' performance is the ultimate benchmark for evaluating the potential value of DL models as tools that assist in clinical decision-making.

Four studies compared the diagnostic accuracy of 13 clinicians for AD with that of DL models. Among the studies, one focused on CTA, two evaluated CT, and one examined NCE-CT. The meta-analysis revealed that the Sen, Spe, PLR, NLR, DOR, and AUSROC were 0.79 (95% CI: 0.65–0.89), 0.92 (95% CI: 0.88–0.94), 9.6 (95% CI: 6.9–13.3), 0.23 (95% CI: 0.13–0.40), 42 (95% CI: 21–82), and 0.94 (95% CI: 0.18–1.00), respectively. Deek's funnel plot indicated no significant publication bias (*P* = 0.22). Given a prior probability of 0.3, when the DL model predicted AD, the probability that the case was truly AD was 0.80 (Supplementary Figures S45–S48). The external validation results for the diagnostic DL models from these four studies were analyzed to compare the performance of models with that of clinicians. The analysis revealed that the Sen, Spe, PLR, NLR, DOR, and AUSROC were 0.93 (95% CI: 0.86–0.97), 0.89 (95% CI: 0.76–0.96), 8.8 (95% CI: 3.7–20.9), 0.08 (95% CI: 0.04–0.15), 117 (95% CI: 48–287), and 0.97 (95% CI: 0.77–1.00), respectively (Supplementary Figures S49, 50).

## 4 Discussion

This systematic review and meta-analysis demonstrated that image-based DL models achieved high accuracy in both segmentation and diagnostic tasks for AD, and their performance was comparable or even superior to that of clinicians in certain scenarios. However, translating these promising algorithmic results into clinical practice requires a critical appraisal of the underlying evidence, especially regarding the substantial heterogeneity, potential for publication bias, and real-world applicability.

In segmentation tasks, DL models demonstrated encouraging potential. Unlike previous reviews that focused on the entire aortic structure, such as Wang et al.'s report of a 96% Dice coefficient for aortic segmentation using DL (58), the present analysis emphasized pathological features specific to AD. These features include the separation of the TL and FL. The pathological core of AD is the intimal flap, which distinguishes the TL from the FL and serves as the key diagnostic criterion (59). Our analysis revealed that DL models achieved high mean Dice coefficients when segmenting the FL, TL, and thrombus. These results suggest that existing DL algorithms can delineate not only the macroscopic contours of large vessels, but also intricate, complex pathological anatomies. This provides a solid foundation for subsequent hemodynamic analysis and surgical planning.

For diagnostic tasks, choosing an imaging modality involves making a trade-off between accuracy and clinical accessibility, where DL models have demonstrated certain value. CTA is currently considered the gold standard for diagnosis (1, 3), offering exceptionally high sensitivity and specificity. However, its clinical application faces limitations related to patient conditions, such as renal insufficiency or contrast agent allergy (60). Additionally, although ECG-gating techniques can reduce motion artifacts, they may produce false-positive results (61). Conversely, non-gated scans risk missing subtle intimal tears and producing false-negative results (62).

The current research showed that DL-assisted diagnosis using non-contrast CT achieved performance comparable to CTA. This observation has significant clinical implications. For high-risk patients unsuitable for CTA, non-contrast CT combined with DL algorithms may be an effective alternative diagnostic strategy. In contrast, DL models based solely on ECGs exhibited lower sensitivity, which is consistent with clinical reality. Approximately 31.3% of acute AD patients present with normal ECG readings (63). Consequently, ECGs are better suited as a low-cost, radiation-free tool for pre-hospital or emergency department triage rather than as a definitive diagnostic method. Moreover, image interpretation by clinicians is inevitably subject to human error, with inter-observer variability potentially leading to misdiagnosis rates of 2%–5% (64). Integrating DL models has the potential to minimize such errors by providing standardized feature extraction.

Although pooled data suggest high diagnostic efficacy, underlying heterogeneity and potential biases require caution.

Firstly, most existing studies in the context of segmentation tasks fail to provide SEs or SDs. The Dice coefficient is calculated using a simple average method, which can introduce certain biases into the results of the quantitative analysis. Therefore, it is important to recognize that the presented mean and SD of Dice coefficients are descriptive statistics intended to provide a narrative summary of the reported accuracy across investigations. Since it was not possible to weight the studies, the results may exhibit heterogeneity and require cautious interpretation. Secondly, existing research demonstrates favorable performance in the segmentation of TL and FL. However, studies on the effects of segmentation related to AD thrombosis are relatively scarce. This limits the ability to further examine the performance of DL on thrombus segmentation for AD.

Regarding diagnostic tasks, certain heterogeneity and potential bias underlying the pooled data were also observed. First, the greatest obstacle to assessing model generalizability is the heterogeneity in validation methods. Most current studies still rely on internal validation methods, such as random splitting or cross-validation. While convenient during development, random splitting often overestimates model performance (65), and cross-validation on small datasets can lead to overfitting and hinder adaptation to specific data distributions (66). Of the 40 diagnostic studies included in this analysis, only five conducted external validation. The scarcity of geographically and equipment-independent external validations makes it difficult to judge the robustness of these models across real-world environments with varying hospitals and scanning parameters (65). Second, the heterogeneity of both the data and the models cannot be overlooked. The low incidence of AD restricts existing training data, resulting in limited sample sizes and a lack of diversity (67). Furthermore, variations in image acquisition protocols and scanning equipment across institutions undermine the generalizability of the model across centers (12). Additionally, there are significant differences in model architecture design. Specialized models developed for specific subtypes (e.g., Type A or Type B) (15, 33) may experience substantial performance degradation when confronted with clinically complex mixed or variant dissections.

Furthermore, although efforts were made to consider potential overlap in patient datasets across the included studies, incomplete reporting in the original literature may have limited the detection of all potential overlaps. This limitation is common in meta-analyses of this type, particularly concerning unreported inter-institutional data sharing. Finally, Deeks' funnel plot indicated the possibility of publication bias in certain subgroup analyses. This implies that studies with small sample sizes, negative results, or failure to achieve expected precision may not be published. Therefore, conclusions that DL models “outperform clinicians” should be interpreted with caution, as the current pooled results may overestimate their actual efficacy.

Translating DL models from the laboratory to clinical practice, especially in time-critical emergency settings, presents unique challenges. In the emergency department, timeliness is paramount. Although models such as ADSeg that incorporate attention mechanisms can improve segmentation accuracy, their complex, cascaded network structures significantly increase computational load and potentially fail to meet the stringent demands of real-time diagnosis (12). Furthermore, emergency CT images often have poor quality (e.g., noise and artifacts), which severely tests model robustness (15).

Concurrently, current models are inadequate for handling complex complications, such as thrombosis. A thrombus can significantly alter vascular morphology, making it more difficult to recognize anatomical structures (67). Furthermore, high-quality annotation of AD data requires experienced radiologists. The high cost of annotation and inter-annotator variability (12, 67) constitute additional obstacles to large-scale model development.

Addressing these issues requires a more targeted approach in the future. Federated learning technology allows for collaborative training using multi-center data without sharing raw data, which effectively breaks down data silos (68). Domain adaptation and meta-learning algorithms can help models rapidly adapt to data distributions from different hospitals and devices, thereby enhancing generalizability (69). Using generative adversarial networks to synthesize imaging data for rare subtypes could improve the balance and diversity of training datasets (70).

Beyond technical hurdles, the clinical integration of DL tools must also overcome regulatory and ethical barriers. Approval from regulatory bodies such as the Food and Drug Administration (FDA), the National Medical Products Administration (NMPA), and the CE mark (indicating Conformité Européenne) should be based on rigorous prospective clinical trials rather than on retrospective study data alone (5, 71). For practical deployment, models must seamlessly integrate into existing Picture Archiving and Communication Systems (PACS) or Radiology Information Systems (RIS) to avoid disrupting clinician workflows (71). Cost-effectiveness is another critical consideration for hospital administrators. They must balance the costs of software procurement and maintenance against the benefits of reducing misdiagnoses and shortening diagnostic time (72). Ultimately, establishing clinical trust is essential. Making model decision logic understandable to clinicians through explainable AI techniques and clarifying accountability between AI recommendations and final clinical decisions are prerequisites for widespread physician acceptance of DL tools (73).

In summary, image-based DL models demonstrated performance comparable to or superior to that of clinicians in AD segmentation and diagnosis. However, this conclusion is substantially influenced by study heterogeneity, limitations in validation methods, and publication bias. The applicability of these models in real-world settings, especially in emergency environments, requires confirmation through large-scale, multicenter, prospective, external validation. Future research should focus on resolving challenges related to model generalizability, real-time performance, and integration with clinical workflows, rather than merely pursuing marginal improvements in algorithmic accuracy.

## 5 Conclusions

This meta-analysis indicates that image-based DL models demonstrate high accuracy in both automatic segmentation and diagnostic tasks for AD. These models perform excellently in segmentation and have a similarly high overall discriminative ability in diagnosis, performing comparably to or better than clinicians. These results support the potential of DL as a clinical auxiliary tool, particularly for reducing diagnostic time. However, the current evidence is limited by substantial inter-study heterogeneity, incomplete reporting of methodological details in some studies, and a lack of rigorous external validation for most models. Therefore, the performance of these models should be interpreted cautiously. Prospective, multicenter studies are needed to validate the clinical utility of the models and address challenges related to their generalizability and integration into real-world practice.

## Data Availability

The original contributions presented in the study are included in the article/Supplementary Material, further inquiries can be directed to the corresponding author/s.
