# Supplementary material for: Performance of image-based deep learning models for aortic dissection segmentation and diagnosis: a systematic review and meta-analysis
Source: Front Cardiovasc Med. 2026 Apr 14;13:1734208. doi: 10.3389/fcvm.2026.1734208 (PMC13121068; doi:10.3389/fcvm.2026.1734208)
Supplement: Supplementary file 1 [file Table1.docx]

**Table S1 Literature search strategy**

**1.Pubmed**

| Search number | Query | Results |
| --- | --- | --- |
| #1 | Aortic Dissection[MeSH Terms] | 22,210 |
| #2 | Aortic Dissection[Title/Abstract] OR Aortic Dissections[Title/Abstract] OR Dissecting Aneurysms[Title/Abstract] OR Dissecting Aneurysm[Title/Abstract] OR Dissecting Aneurysm Aorta[Title/Abstract] OR Dissecting Aneurysm Aortas[Title/Abstract] OR Aortic Dissecting Aneurysm[Title/Abstract] OR Aortic Dissecting Aneurysms[Title/Abstract] OR Aortic Arch Dissection[Title/Abstract] OR Aortic Arch Dissections[Title/Abstract] OR Descending Aorta Dissections[Title/Abstract] OR Descending Aorta Dissection[Title/Abstract] OR Thoracic Aorta Dissection[Title/Abstract] | 23,065 |
| #3 | Deep learning[MeSH Terms] | 23,572 |
| #4 | deep learning[Title/Abstract] OR Computer aided diagnostic system[Title/Abstract] OR Computer-aided diagnostic system[Title/Abstract] OR Transfer Learning[Title/Abstract] OR Ensemble Learning[Title/Abstract] OR artificial intelligence[Title/Abstract] OR Hierarchical Learning[Title/Abstract] OR Machine Learning[Title/Abstract] OR Neural Networks[Title/Abstract] OR Neural Network[Title/Abstract] OR CNN[Title/Abstract] OR AlexNet[Title/Abstract] OR VGGNet[Title/Abstract] OR ResNet[Title/Abstract] OR GoogLeNet[Title/Abstract] OR DenseNet[Title/Abstract] OR MobileNet[Title/Abstract] OR EfficientNet[Title/Abstract] OR VGG-11[Title/Abstract] OR VGG-13[Title/Abstract] OR VGG-16[Title/Abstract] OR VGG-19[Title/Abstract] OR VGG11[Title/Abstract] OR VGG13[Title/Abstract] OR VGG16[Title/Abstract] OR VGG19[Title/Abstract] OR ResNet50[Title/Abstract] OR ResNet101[Title/Abstract] OR ResNet34[Title/Abstract] OR ResNet18[Title/Abstract] OR Long Short-Term Memory[Title/Abstract] OR LSTM[Title/Abstract] | 296,769 |
| #5 | ((Aortic Dissection[MeSH Terms]) OR (Aortic Dissection[Title/Abstract] OR Aortic Dissections[Title/Abstract] OR Dissecting Aneurysms[Title/Abstract] OR Dissecting Aneurysm[Title/Abstract] OR Dissecting Aneurysm Aorta[Title/Abstract] OR Dissecting Aneurysm Aortas[Title/Abstract] OR Aortic Dissecting Aneurysm[Title/Abstract] OR Aortic Dissecting Aneurysms[Title/Abstract] OR Aortic Arch Dissection[Title/Abstract] OR Aortic Arch Dissections[Title/Abstract] OR Descending Aorta Dissections[Title/Abstract] OR Descending Aorta Dissection[Title/Abstract] OR Thoracic Aorta Dissection[Title/Abstract])) AND ((Deep learning[MeSH Terms]) OR (deep learning[Title/Abstract] OR Computer aided diagnostic system[Title/Abstract] OR Computer-aided diagnostic system[Title/Abstract] OR Transfer Learning[Title/Abstract] OR Ensemble Learning[Title/Abstract] OR artificial intelligence[Title/Abstract] OR Hierarchical Learning[Title/Abstract] OR Machine Learning[Title/Abstract] OR Neural Networks[Title/Abstract] OR Neural Network[Title/Abstract] OR CNN[Title/Abstract] OR AlexNet[Title/Abstract] OR VGGNet[Title/Abstract] OR ResNet[Title/Abstract] OR GoogLeNet[Title/Abstract] OR DenseNet[Title/Abstract] OR MobileNet[Title/Abstract] OR EfficientNet[Title/Abstract] OR VGG-11[Title/Abstract] OR VGG-13[Title/Abstract] OR VGG-16[Title/Abstract] OR VGG-19[Title/Abstract] OR VGG11[Title/Abstract] OR VGG13[Title/Abstract] OR VGG16[Title/Abstract] OR VGG19[Title/Abstract] OR ResNet50[Title/Abstract] OR ResNet101[Title/Abstract] OR ResNet34[Title/Abstract] OR ResNet18[Title/Abstract] OR Long Short-Term Memory[Title/Abstract] OR LSTM[Title/Abstract])) | 114 |

**2.Cochrane**

| Search number | Query | Results |
| --- | --- | --- |
| #1 | MeSH descriptor: [Aortic Dissection] explode all trees | 228 |
| #2 | (Aortic Dissection OR Aortic Dissections OR Dissecting Aneurysms OR Dissecting Aneurysm OR Dissecting Aneurysm Aorta OR Dissecting Aneurysm Aortas OR Aortic Dissecting Aneurysm OR Aortic Dissecting Aneurysms OR Aortic Arch Dissection OR Aortic Arch Dissections OR Descending Aorta Dissections OR Descending Aorta Dissection OR Thoracic Aorta Dissection):ti,ab,kw | 905 |
| #3 | MeSH descriptor: [Deep Learning] explode all trees | 339 |
| #4 | (deep learning OR Computer aided diagnostic system OR Computer-aided diagnostic system OR Transfer Learning OR Ensemble Learning OR artificial intelligence OR Hierarchical Learning OR Machine Learning OR Neural Networks OR Neural Network OR CNN OR AlexNet OR VGGNet OR ResNet OR GoogLeNet OR DenseNet OR MobileNet OR EfficientNet OR VGG-11 OR VGG-13 OR VGG-16 OR VGG-19 OR VGG11 OR VGG13 OR VGG16 OR VGG19 OR ResNet50 OR ResNet101 OR ResNet34 OR ResNet18 OR Long Short-Term Memory OR LSTM):ti,ab,kw | 11,777 |
| #5 | (#1 OR #2) AND (#3 OR #4) | 5 |

**3.Embase**

| Search number | Query | Results |
| --- | --- | --- |
| #1 | 'aortic dissection'/exp | 31,429 |
| #2 | 'aortic dissection':ti,ab OR 'aortic dissections':ti,ab OR 'dissecting aneurysms':ti,ab OR 'dissecting aneurysm':ti,ab OR 'dissecting aneurysm aorta':ti,ab OR 'dissecting aneurysm aortas':ti,ab OR 'aortic dissecting aneurysm':ti,ab OR 'aortic dissecting aneurysms':ti,ab OR 'aortic arch dissection':ti,ab OR 'aortic arch dissections':ti,ab OR 'descending aorta dissections':ti,ab OR 'descending aorta dissection':ti,ab OR 'thoracic aorta dissection':ti,ab | 29,076 |
| #3 | 'deep learning'/exp | 66,197 |
| #4 | 'deep learning':ti,ab OR 'computer aided diagnostic system':ti,ab OR 'computer-aided diagnostic system':ti,ab OR 'transfer learning':ti,ab OR 'ensemble learning':ti,ab OR 'artificial intelligence':ti,ab OR 'hierarchical learning':ti,ab OR 'machine learning':ti,ab OR 'neural networks':ti,ab OR 'neural network':ti,ab OR 'cnn':ti,ab OR 'alexnet':ti,ab OR 'vggnet':ti,ab OR 'resnet':ti,ab OR 'googlenet':ti,ab OR 'densenet':ti,ab OR 'mobilenet':ti,ab OR 'efficientnet':ti,ab OR 'vgg-11':ti,ab OR 'vgg-13':ti,ab OR 'vgg-16':ti,ab OR 'vgg-19':ti,ab OR 'vgg11':ti,ab OR 'vgg13':ti,ab OR 'vgg16':ti,ab OR 'vgg19':ti,ab OR 'resnet50':ti,ab OR 'resnet101':ti,ab OR 'resnet34':ti,ab OR 'resnet18':ti,ab OR 'long short-term memory':ti,ab OR 'lstm':ti,ab | 322,568 |
| #5 | ('aortic dissection'/exp OR 'aortic dissection' OR 'aortic dissection':ti,ab OR 'aortic dissections':ti,ab OR 'dissecting aneurysms':ti,ab OR 'dissecting aneurysm':ti,ab OR 'dissecting aneurysm aorta':ti,ab OR 'dissecting aneurysm aortas':ti,ab OR 'aortic dissecting aneurysm':ti,ab OR 'aortic dissecting aneurysms':ti,ab OR 'aortic arch dissection':ti,ab OR 'aortic arch dissections':ti,ab OR 'descending aorta dissections':ti,ab OR 'descending aorta dissection':ti,ab OR 'thoracic aorta dissection':ti,ab) AND ('deep learning'/exp OR 'deep learning' OR 'deep learning':ti,ab OR 'computer aided diagnostic system':ti,ab OR 'computer-aided diagnostic system':ti,ab OR 'transfer learning':ti,ab OR 'ensemble learning':ti,ab OR 'artificial intelligence':ti,ab OR 'hierarchical learning':ti,ab OR 'machine learning':ti,ab OR 'neural networks':ti,ab OR 'neural network':ti,ab OR 'cnn':ti,ab OR 'alexnet':ti,ab OR 'vggnet':ti,ab OR 'resnet':ti,ab OR 'googlenet':ti,ab OR 'densenet':ti,ab OR 'mobilenet':ti,ab OR 'efficientnet':ti,ab OR 'vgg-11':ti,ab OR 'vgg-13':ti,ab OR 'vgg-16':ti,ab OR 'vgg-19':ti,ab OR 'vgg11':ti,ab OR 'vgg13':ti,ab OR 'vgg16':ti,ab OR 'vgg19':ti,ab OR 'resnet50':ti,ab OR 'resnet101':ti,ab OR 'resnet34':ti,ab OR 'resnet18':ti,ab OR 'long short-term memory':ti,ab OR 'lstm':ti,ab) | 160 |

**4.Web of science**

| Search number | Query | Results |
| --- | --- | --- |
| #1 | TS=(Aortic Dissection) and Preprint Citation Index (Exclude – Database) | 37,642 |
| #2 | TS=(Aortic Dissection OR Aortic Dissections OR Dissecting Aneurysms OR Dissecting Aneurysm OR Dissecting Aneurysm Aorta OR Dissecting Aneurysm Aortas OR Aortic Dissecting Aneurysm OR Aortic Dissecting Aneurysms OR Aortic Arch Dissection OR Aortic Arch Dissections OR Descending Aorta Dissections OR Descending Aorta Dissection OR Thoracic Aorta Dissection) and Preprint Citation Index (Exclude – Database) | 48,611 |
| #3 | TS=(deep learning) and Preprint Citation Index (Exclude – Database) | 345,183 |
| #4 | TS=(deep learning OR Computer aided diagnostic system OR Computer-aided diagnostic system OR Transfer Learning OR Ensemble Learning OR artificial intelligence OR Hierarchical Learning OR Machine Learning OR Neural Networks OR Neural Network OR CNN OR AlexNet OR VGGNet OR ResNet OR GoogLeNet OR DenseNet OR MobileNet OR EfficientNet OR VGG-11 OR VGG-13 OR VGG-16 OR VGG-19 OR VGG11 OR VGG13 OR VGG16 OR VGG19 OR ResNet50 OR ResNet101 OR ResNet34 OR ResNet18 OR Long Short-Term Memory OR LSTM) and Preprint Citation Index (Exclude – Database) | 1,506,346 |
| #5 | TS=(Aortic Dissection OR Aortic Dissections OR Dissecting Aneurysms OR Dissecting Aneurysm OR Dissecting Aneurysm Aorta OR Dissecting Aneurysm Aortas OR Aortic Dissecting Aneurysm OR Aortic Dissecting Aneurysms OR Aortic Arch Dissection OR Aortic Arch Dissections OR Descending Aorta Dissections OR Descending Aorta Dissection OR Thoracic Aorta Dissection) AND TS=(deep learning OR Computer aided diagnostic system OR Computer-aided diagnostic system OR Transfer Learning OR Ensemble Learning OR artificial intelligence OR Hierarchical Learning OR Machine Learning OR Neural Networks OR Neural Network OR CNN OR AlexNet OR VGGNet OR ResNet OR GoogLeNet OR DenseNet OR MobileNet OR EfficientNet OR VGG-11 OR VGG-13 OR VGG-16 OR VGG-19 OR VGG11 OR VGG13 OR VGG16 OR VGG19 OR ResNet50 OR ResNet101 OR ResNet34 OR ResNet18 OR Long Short-Term Memory OR LSTM) | 289 |
