# Supplementary material for: Performance of image-based deep learning models for aortic dissection segmentation and diagnosis: a systematic review and meta-analysis
Source: Front Cardiovasc Med. 2026 Apr 14;13:1734208. doi: 10.3389/fcvm.2026.1734208 (PMC13121068; doi:10.3389/fcvm.2026.1734208)
Supplement: Supplementary file 2 [file Table2.docx]

Table S2 Basic characteristics for articles on segmentation tasks

| No. | First author | Publication year | Country of author | Patient source | Image source | Total number of cases | Total number of cases in training set | Generation method of validation set | Number of cases in validation set |
| --- | --- | --- | --- | --- | --- | --- | --- | --- | --- |
| 1 | Dongqiao Xiang[12] | 2023 | China | Multicenter | CTA | P:108 | P:68 | Cross-validation | P:40 |
| 2 | Christos Mavridis[13] | 2024 | Greece | Single center | CT | P:16 3D CT data sets | P:16 3D CT data sets |  |  |
| 3 | Hanying Feng[14] | 2023 | China | Single center | CTA | P:463 CTA scans | P:463 CTA scans | 5-fold cross-validation |  |
| 4 | Xiaoya Guo[15] | 2024 | China | Single center | CT | P:24 | P:18 | Cross-validation | P:6 |
| 5 | Xin Fan[16] | 2022 | China | Single center: The First Affiliated Hospital of Xinjiang Medical University | CT | P:7500 CT scan slices | P:4500 CT scan slices | Random sampling | P:1500 CT scan slices |
| 6 | C.Shao[38] | 2019 | France | Multicenter | CT |  | P:35CT scan slices | Random sampling | P:10 CT scan slices |
| 7 | Lewis D.Hahn[17] | 2020 | USA | Single center | CTA | P:45 (B) 153 CTA images | P:30 (B) 103 CTA images | Random sampling | P:7 (B) 22 CTA images |
| 8 | Junlong Cheng[18] | 2020 | China | Single center | CT | P:749 images |  | Random sampling |  |
| 9 | Lu-Feng Chen[19] | 2023 | China |  | CT |  |  |  |  |
| 10 | Hyun Jung Koo[20] | 2024 | Korea | Single center | CT | P: 704 healthy people | P:138 | Random sampling | P:35 |
| 11 | Risto Kesävuori[21] | 2023 | Finland | Multicenter | CTA | P:206 (B) | P:136 | Random sampling | P:30 |
| 12 | Liana D. Wobben[22] | 2021 | USA | Single center | CTA | P:40 (B) 147 CTA images | P:103 CTA images | Random sampling | P:22 CTA images |
| 13 | Xuyang Zhang[23] | 2023 | China | Multicenter | CT | P:133 (B) 306 CT images |  | Cross-validation |  |
| 14 | Tianling Lyu[24] | 2021 | China | Single center | CTA | P:42 | P:35 | Cross-validation | P:7 |
| 15 | BRADLEY FEIGER&ERICK LORENZANA- SALDIVAR [25] | 2022 | USA | Single center | CTA | P:21 (B) | P:15 (B) | Cross-validation | P:2 (B) |
| 16 | Arian Aghilinejad[26] | 2023 | USA | Single center | CT | P:19 (B) | P:15 (B) | Random sampling | P:4 (B) |
| 17 | Long Cao[27] | 2019 | China | Registration database (multicenter) | CTA | P:276 (B) | P:246 (B) | Random sampling Cross-validation | P:30 (B) |
| 18 | Zeyang Yao[28] | 2021 | China | Single center | CTA | P:100 (B) |  | Cross-validation |  |
| 19 | Qingyang Zhou[36] | 2022 | China | Single center | CT | P:35 participants (21 patients and 14 healthy controls) | P:29 (3707 CT slices) | Random sampling | P:6 (719 CT slices) |
| 20 | Duanduan Chen[29] | 2021 | China | Multicenter | CTA | P:120 (B) | P:80 (B) | Cross-validation | P:20 (B) |
| 21 | Weiya Sun[30] | 2022 | China |  | DSA | P:80 | P:64 (2048 DSA frames) | Cross-validation | P:16 (512 DSA frames) |
| 22 | Simone Bonechi[31] | 2021 | Italy | Single center | CT | P:154 images | P:134 images | Random sampling | P:10 images |
| 23 | Jingliang Zhao[32] | 2022 | China | Single center | CTA | P:35 | P:35 | Random sampling |  |
| 24 | Jinhui Zhang[37] | 2023 | China | Single center | CTA/MRI | P:100 (B) | P:80 (B) | Random sampling | P:20 (B) |
| 25 | Yitong Yu[33] | 2021 | China | Single center | CT | P:139 (B) | P:99 (B) | Random sampling | P:15 (B) |
| 26 | Jonathan R. Krebs[2] | 2024 | USA | Single center | CTA | P:59 (B) |  | 4-fold cross-validation |  |
| 27 | Ji-Hoon Jung[34] | 2024 | Korea | Multicenter | CT | P:173 | P:173 for training and validation, 80 for internal test | Random sampling | P:173 for training and validation, 80for internal test |
| 28 | Hongwei Chen[35] | 2022 | China | Single center | CT/CTA | P:100 | P:704 CT&CTA images | Random sampling | P:410 CT&CTA images |
