# Supplementary material for: Performance of image-based deep learning models for aortic dissection segmentation and diagnosis: a systematic review and meta-analysis
Source: Front Cardiovasc Med. 2026 Apr 14;13:1734208. doi: 10.3389/fcvm.2026.1734208 (PMC13121068; doi:10.3389/fcvm.2026.1734208)
Supplement: Supplementary file 3 [file Table3.docx]

Table S3 Basic characteristics for articles on diagnostic tasks

| No. | First author | Publication year | Country of author | Patient source | Task type | Image source | Segmentation method | Number of aortic dissection cases | Total number of cases | Total number of cases in training set | Generation method of validation set | Number of cases in validation set | Model type |
| --- | --- | --- | --- | --- | --- | --- | --- | --- | --- | --- | --- | --- | --- |
| 1 | Yi Yan[39] | 2022 | China | Multicenter | Diagnosis | CTA | Automatic segmentation | P:185 | P:452 | P:238 | Multicenter |  | Resnet Gaussian NB |
| 2 | Haikuo Peng[40] | 2022 | China |  | Diagnosis | CT | Automatic segmentation | P:70(A)/18(B) | P:105 | P:68 | Random sampling | P:37 | Resnet EfficientNetV2 DenseNet-121 |
| 3 | Tomoki Wada[41] | 2023 | Japan | Single center | Diagnosis | CTA | Manual segmentation |  | P:209 | P:108 | Cross-validation | P:132 | DCNN |
| 4 | Li‑Ting Huang[42] | 2022 | China | Single center | Diagnosis | CTA | Automatic segmentation | P:57(A)/43(B) | P:130 |  | Cross-validation |  | U-Net ResNet |
| 5 | Yun Tan[43] | 2020 | China | Single center | Diagnosis | CTA |  | P:43 (2368 slice images) | P:88 (4840 slice images) | 2904 slice images | Random sampling |  | DenseNet Resnet InceptionV3 |
| 6 | Xiangyu Xiong[44] | 2022 | China | Multicenter | Diagnosis | NCE-CT |  | P:65 | P:154 |  | Cross-validation |  |  |
| 7 | Robert J.Harris[45] | 2019 | USA | Single center | Diagnosis | CT | Manual segmentation | P:34196 slice images | P:87479 slice images | P:778 (81047 slice images) | Random sampling | P:43 (6432 slice images) | NLP CNN |
| 8 | Akinori Hata[46] | 2021 | Japan | Single center | Diagnosis | CT | Automatic segmentation | P:85 | P:170 |  | Cross-validation |  | CNN of Xception |
| 9 | Zhangbo Cheng[47] | 2024 | China | Multicenter | Diagnosis | NCE-CT | Manual segmentation | P:75(A)/85(B) | P:320 | 206 | Random sampling | 50 | 3D full-resolution U-Net model |
| 10 | Junlong Cheng[18] | 2020 | China |  | Segmentation and diagnosis | CT |  | P:10 | P:20 | P:5000 slices | Random sampling | P:5000 slices | U-Net (segmentation) |
| 11 | Zeye Liu[48] | 2024 | China | Multicenter | Diagnosis | CTA |  | P:63(A)/74(B) | P:1303 |  |  |  | U-Net |
| 12 | Minghe Zhou[49] | 2024 | China | Single center | Diagnosis | ECGs |  | P:313 | P:1878 | P:1096 | Random sampling |  |  |
| 13 | WeiTing Liu[50] | 2022 | China | Single center | Diagnosis | ECGs, CXR (chest X-rays) |  |  |  | ECGs:P:33222 (45806 records) CXR:P:30309 (43365 records) | Cross-validation | ECGs:P:7715 (8905 records) CXR:P:7014 (8430 records) | ECGs:ECG12Net CXR:DenseNet |
| 14 | Dong Keon Lee[51] | 2022 | Korea | Multicenter | Diagnosis | X-ray |  | P:716 | P:3331 |  | Cross-validation |  | Resnet |
| 15 | Vladimir Laletin[58] | 2024 | France | Multicenter | Segmentation and diagnosis | CTA | Manual segmentation | P:63(A)/74(B) | P:1303 |  | Retrospective study |  | CNNs |
| 16 | Zhihui Huang[53] | 2024 | China | Single center | Diagnosis | CTA | Automatic segmentation |  |  | P:18892 images | Random sampling |  | CNNs |
| 17 | Takuto Arita[54] | 2024 | Japan | Single center | Diagnosis | ECGs |  | P:115(A)/32(B) | P:19170 | Four out of five groups | Cross-validation |  | CNN (Resnet) |
| 18 | Fenglei Dong[55] | 2024 | China | Multicenter | Diagnosis | CT | Manual segmentation | P:90(A)/150(B) | P:480 These cases were finally included, and some other cases were excluded. | P:320 | Internal validation, Random sampling, external validation | Internal validation:P:80 external validation:P:80 | YOLOv5 (deep CNN) |
| 19 | Linlong He[56] | 2024 | China |  | Diagnosis | CTA |  | P:40 | P:80 | P:48 | 5-fold cross-validation | P:16 | DAT-DenseNet |
| 20 | Anish Raj[57] | 2024 | Germany | Multicenter | Diagnosis | CT | Automatic segmentation | P:94 | P:195 | P:163 | Cross-validation center validation | P:32 | CNN |
