# Supplementary material for: Performance of image-based deep learning models for aortic dissection segmentation and diagnosis: a systematic review and meta-analysis
Source: Front Cardiovasc Med. 2026 Apr 14;13:1734208. doi: 10.3389/fcvm.2026.1734208 (PMC13121068; doi:10.3389/fcvm.2026.1734208)
Supplement: Supplementary file 4 [file Supplementaryfile1.docx]

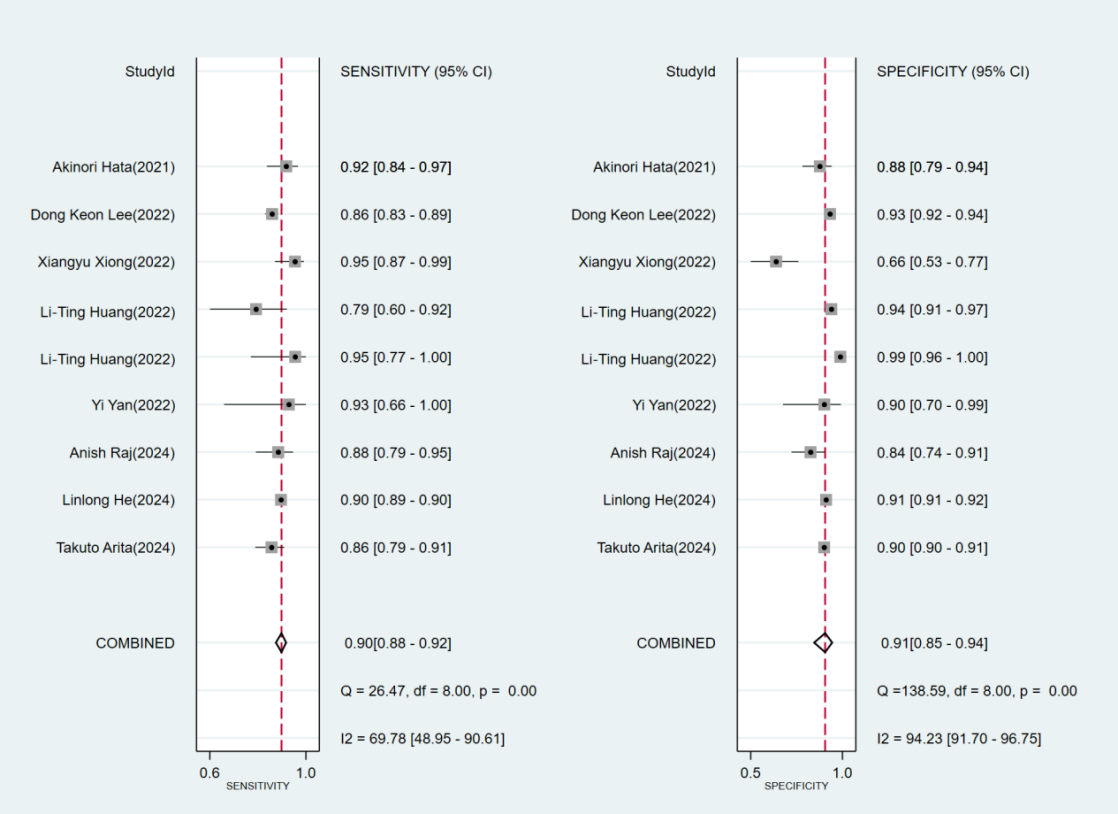
Figure S1 Forest plot for sensitivity and specialty of DL


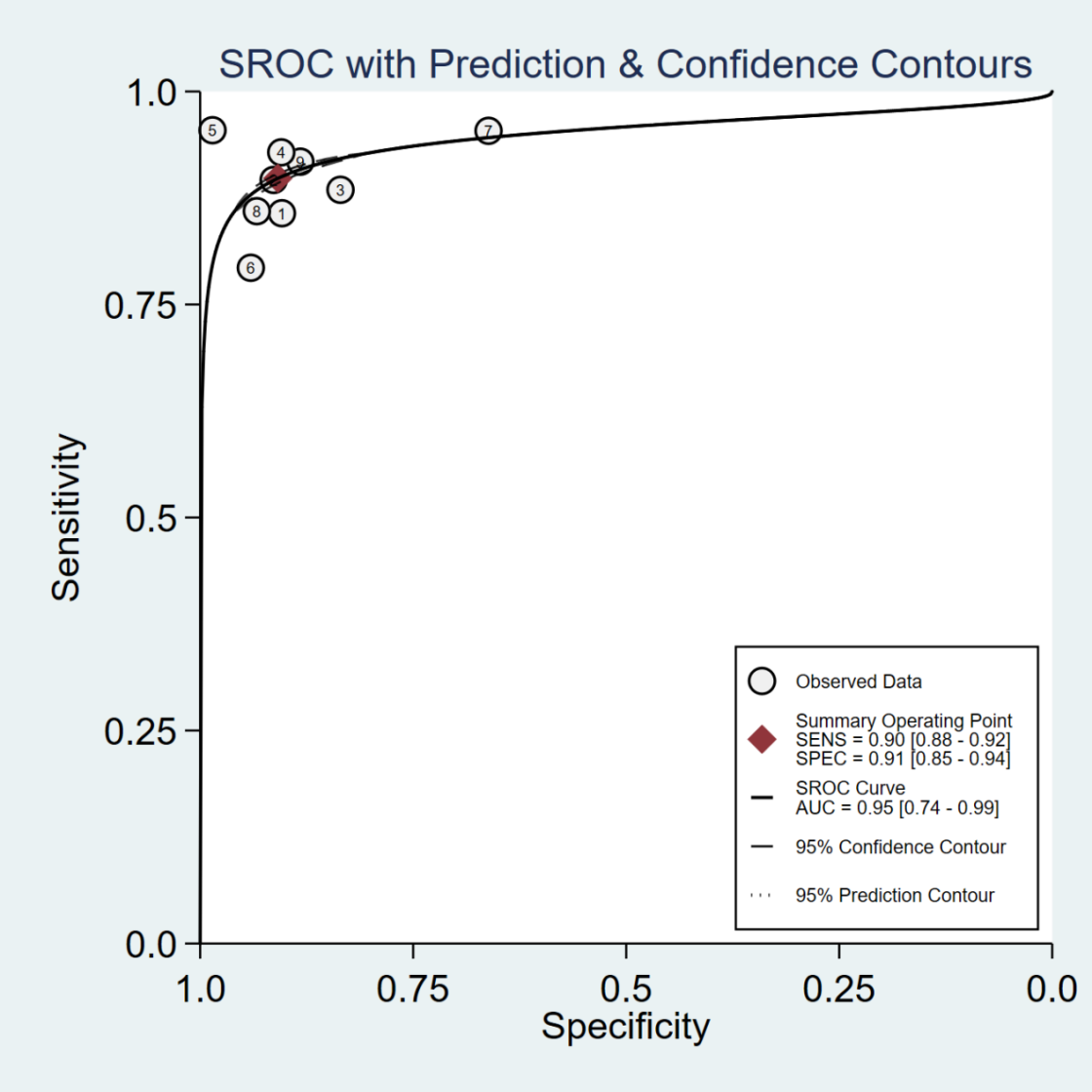
Figure S2 SROC curve for sensitivity and specialty of DL


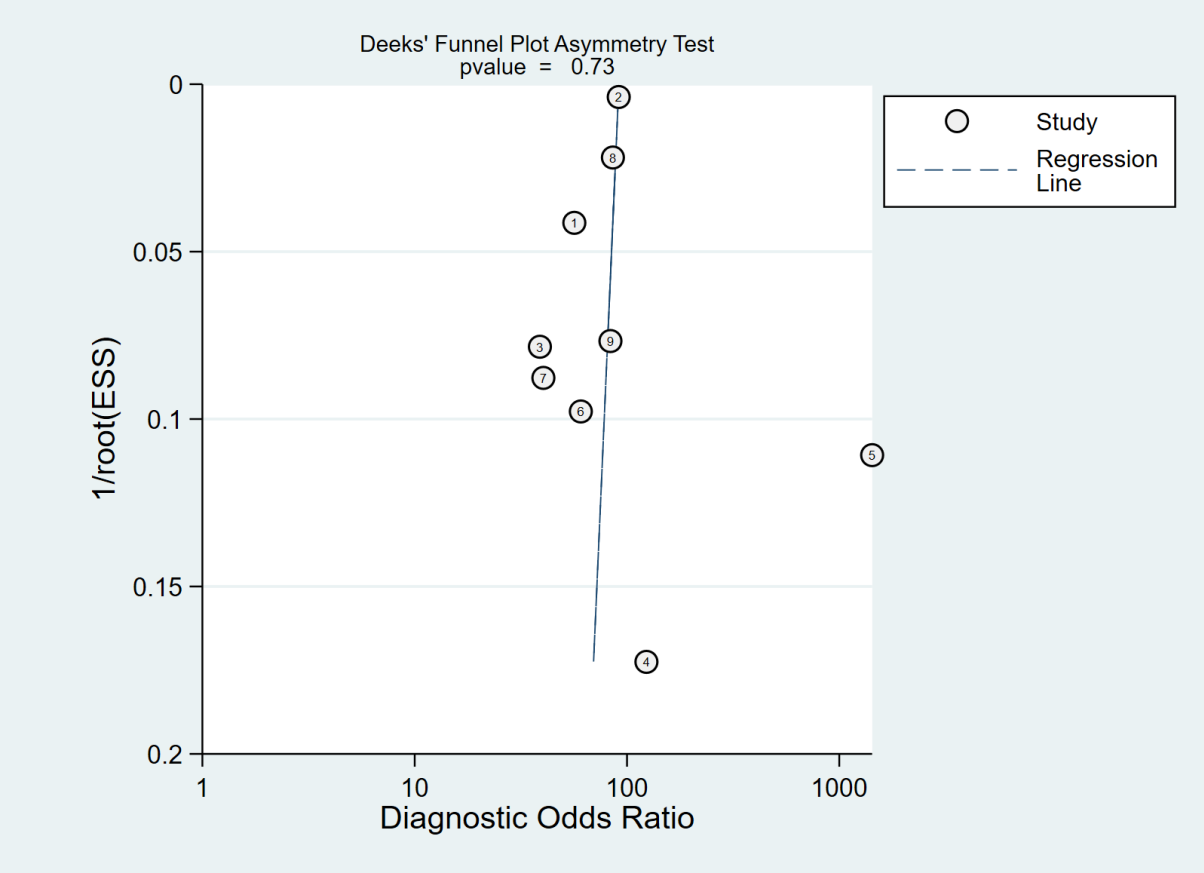
Figure S3 Deek’s funnel plot for sensitivity and specialty of DL


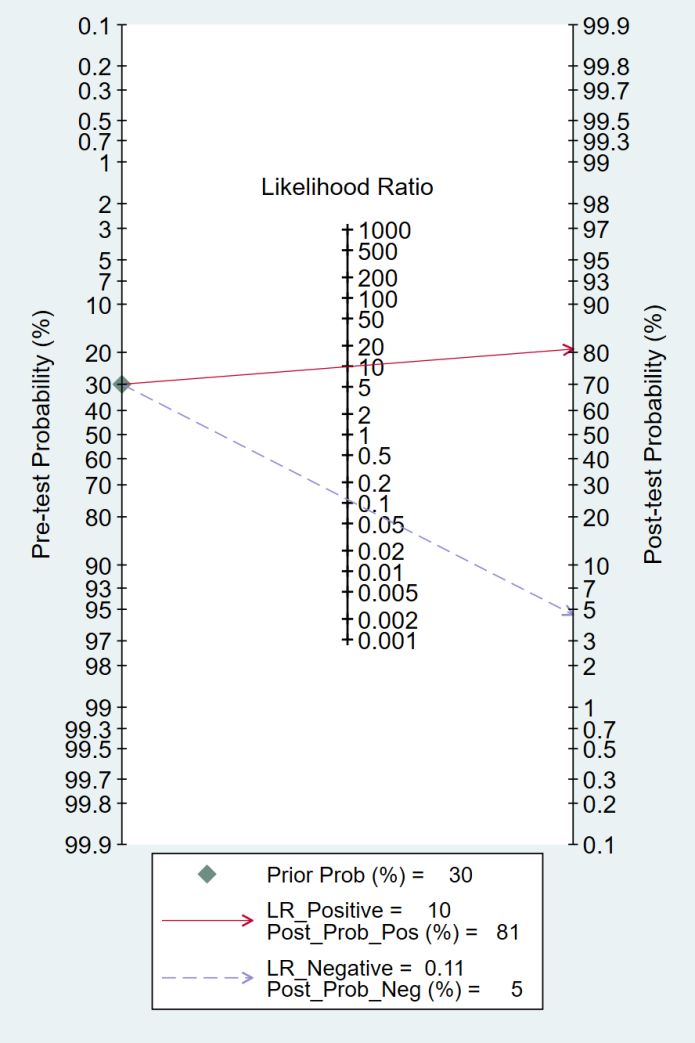


Figure S4 Fagan’s nomogram for sensitivity and specialty of DL


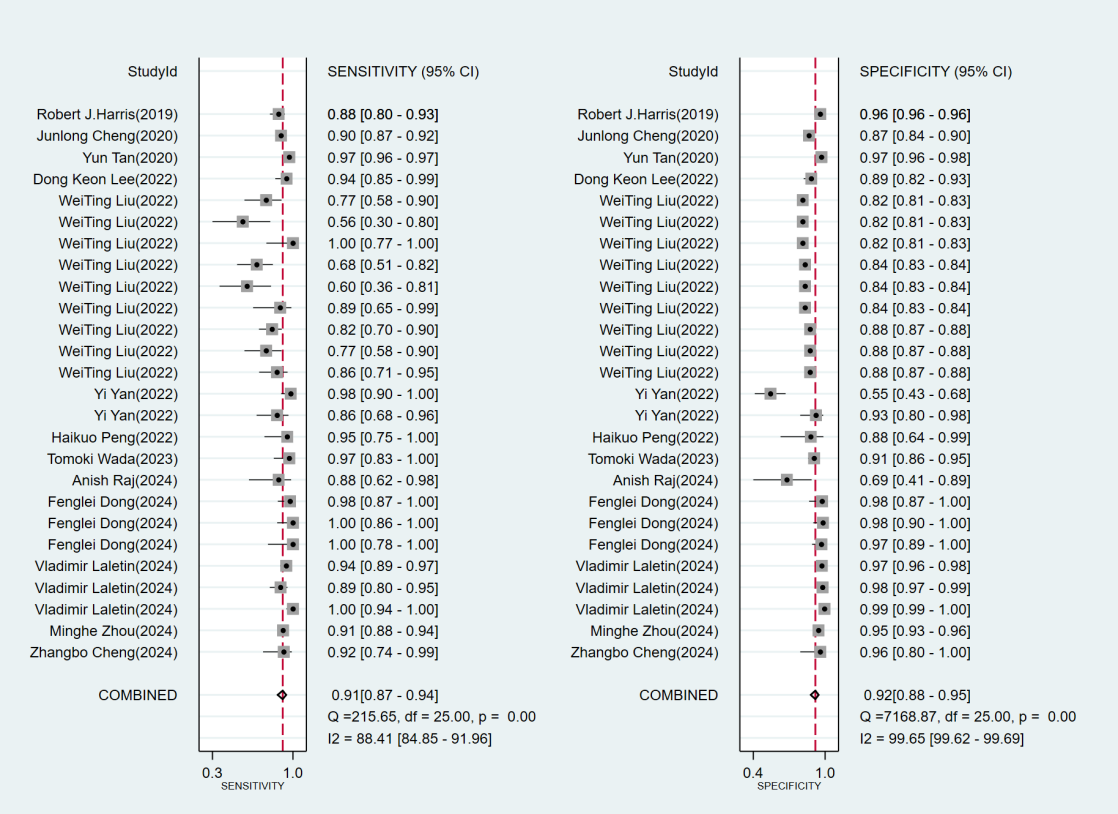
Figure S5 Forest plot for sensitivity and specialty of DL


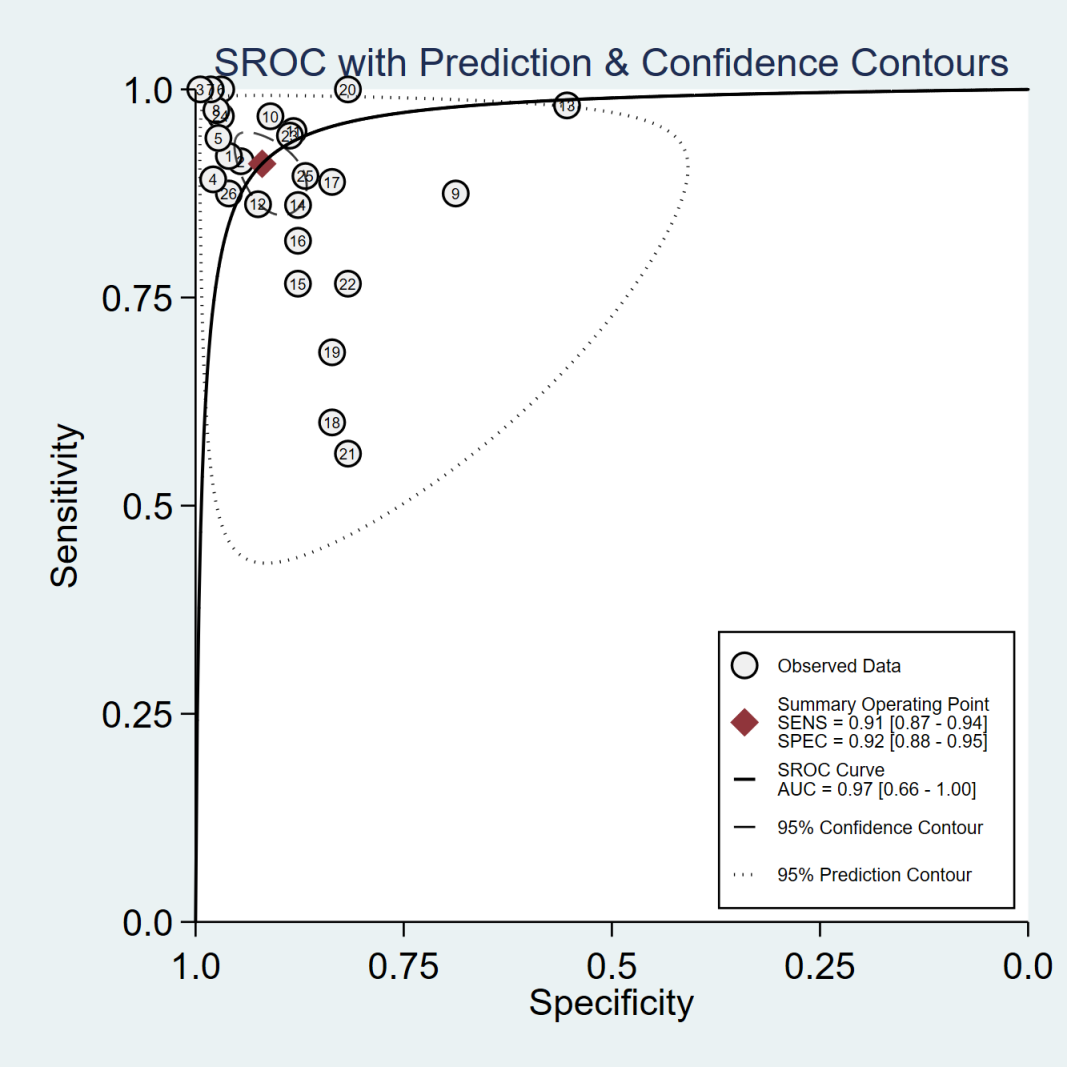
Figure S6 SROC curve for sensitivity and specialty of DL


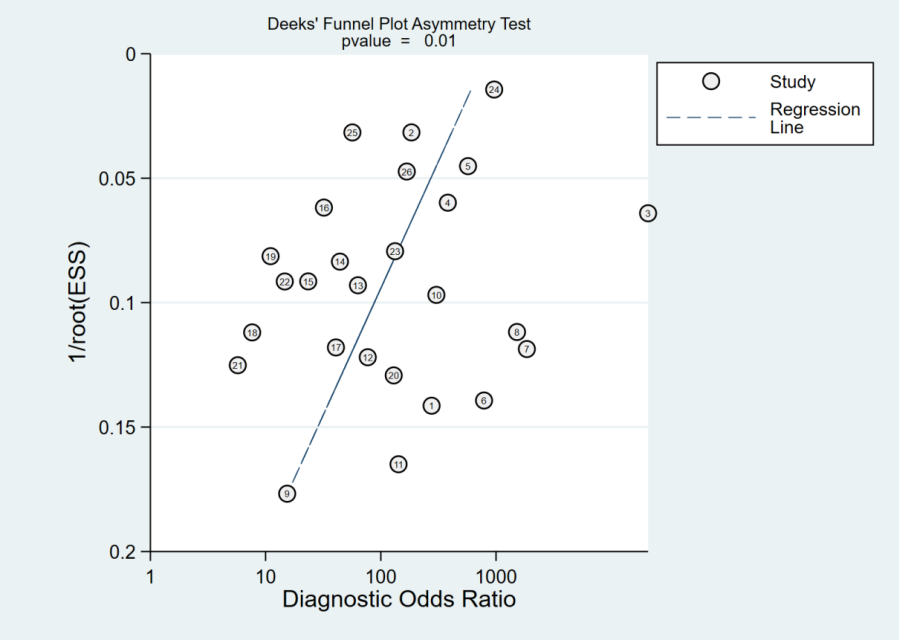
Figure S7 Deek’s funnel plot for sensitivity and specialty of DL


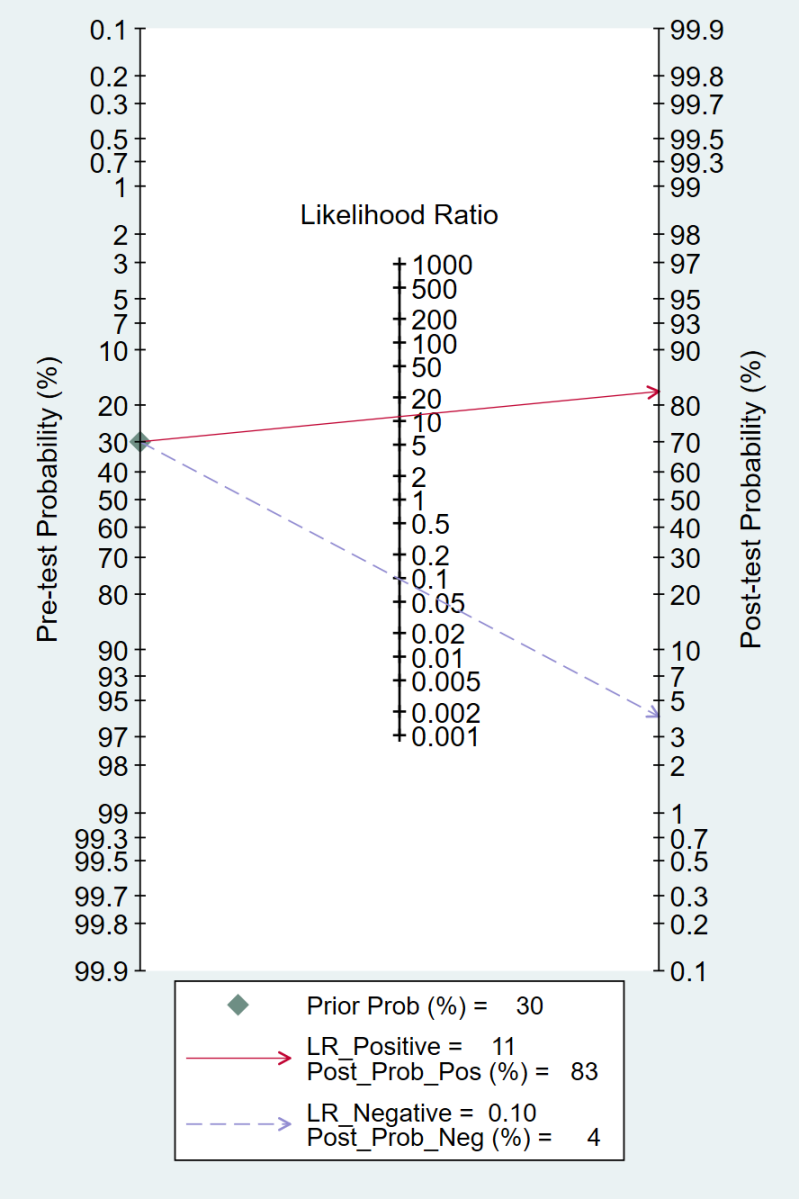
Figure S8 Fagan’s nomogram for sensitivity and specialty of DL


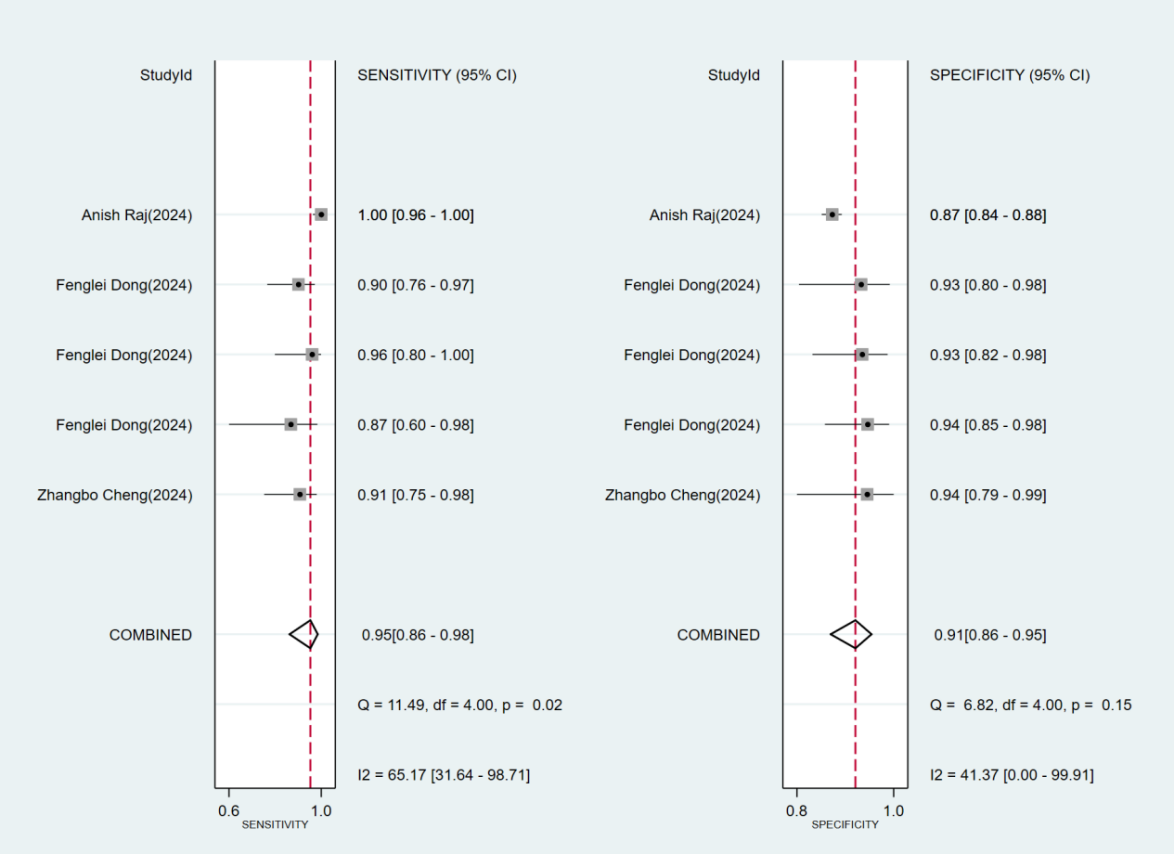


Figure S9 Forest plot for sensitivity and specialty of DL


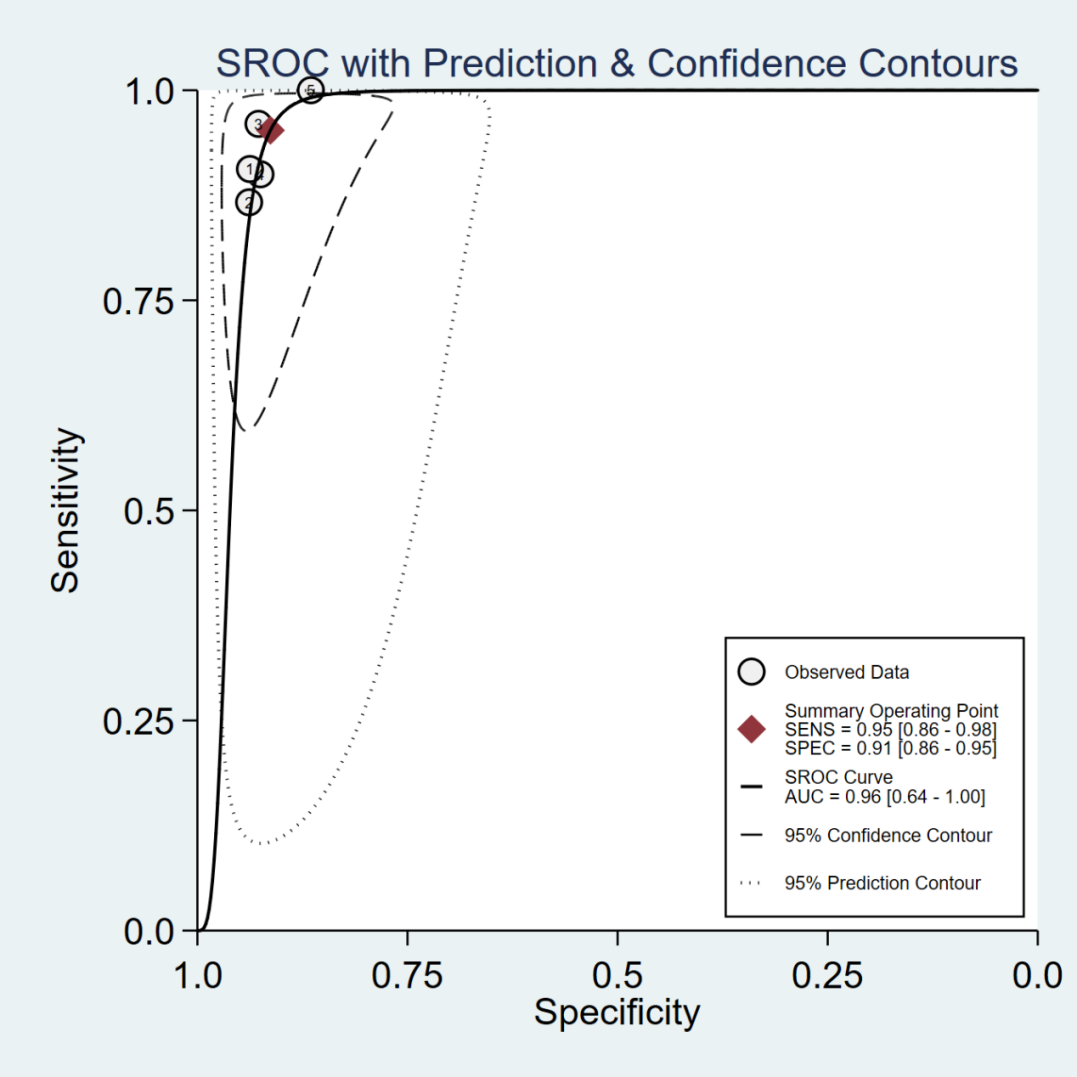


Figure S10 SROC curve for sensitivity and specialty of DL
