# Supplementary material for: Performance of image-based deep learning models for aortic dissection segmentation and diagnosis: a systematic review and meta-analysis
Source: Front Cardiovasc Med. 2026 Apr 14;13:1734208. doi: 10.3389/fcvm.2026.1734208 (PMC13121068; doi:10.3389/fcvm.2026.1734208)
Supplement: Supplementary file 5 [file Supplementaryfile2.docx]

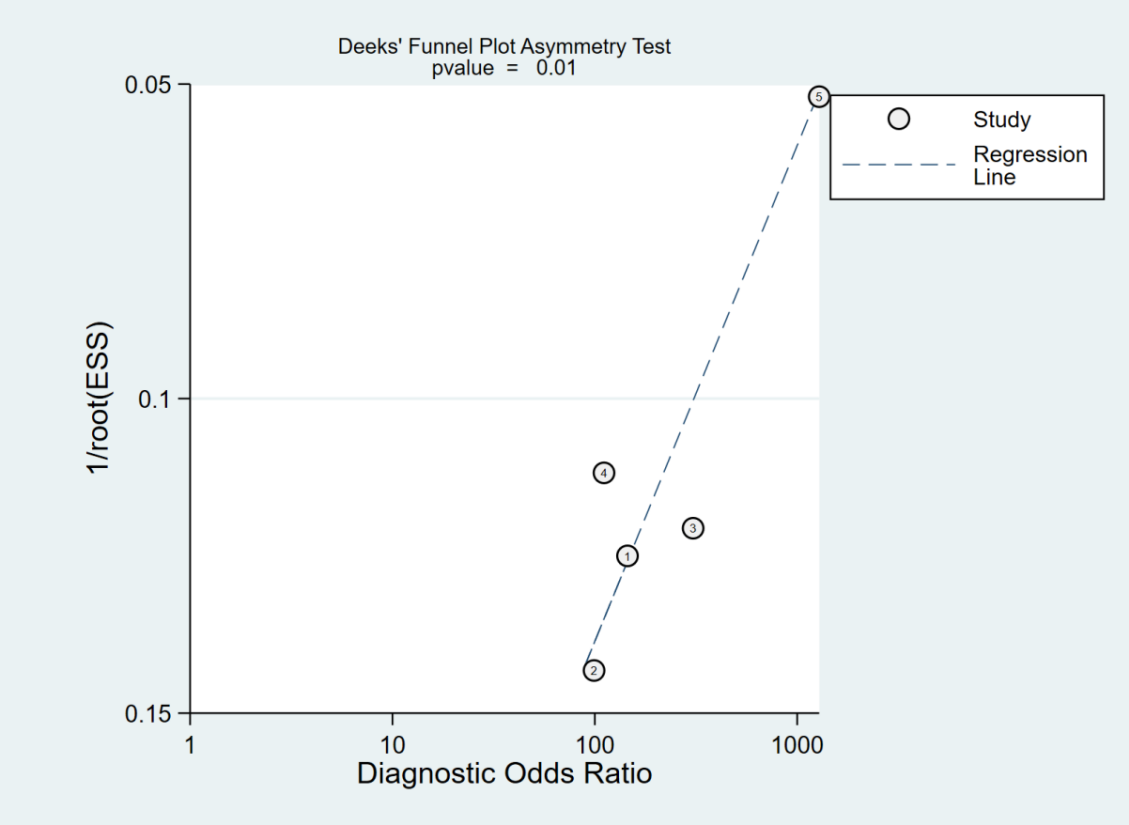


Figure S11 Deek’s funnel plot for sensitivity and specialty of DL


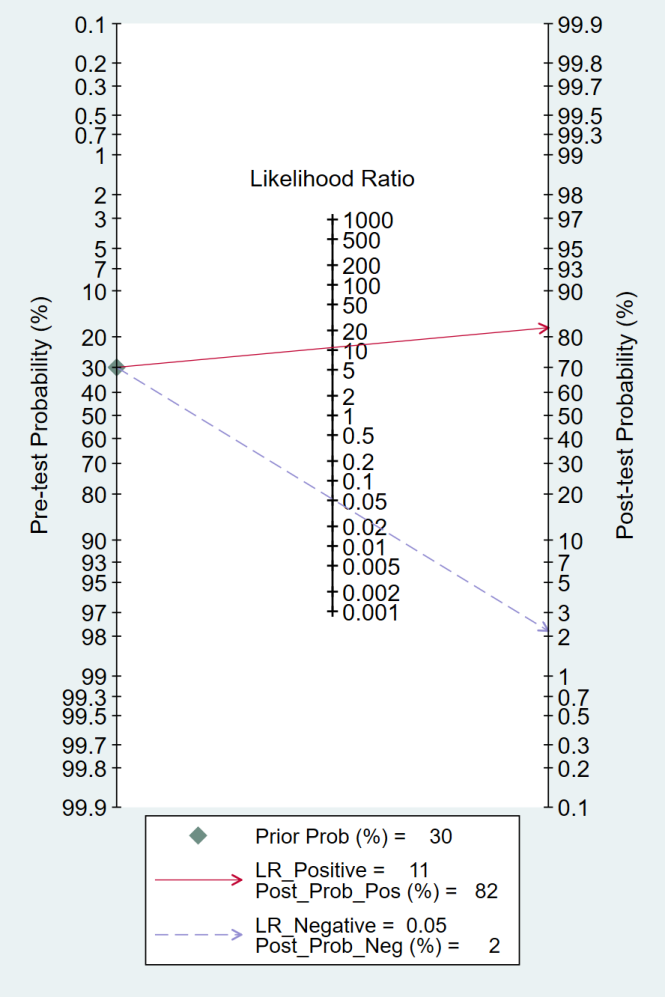


Figure S12 Fagan’s nomogram for sensitivity and specialty of DL


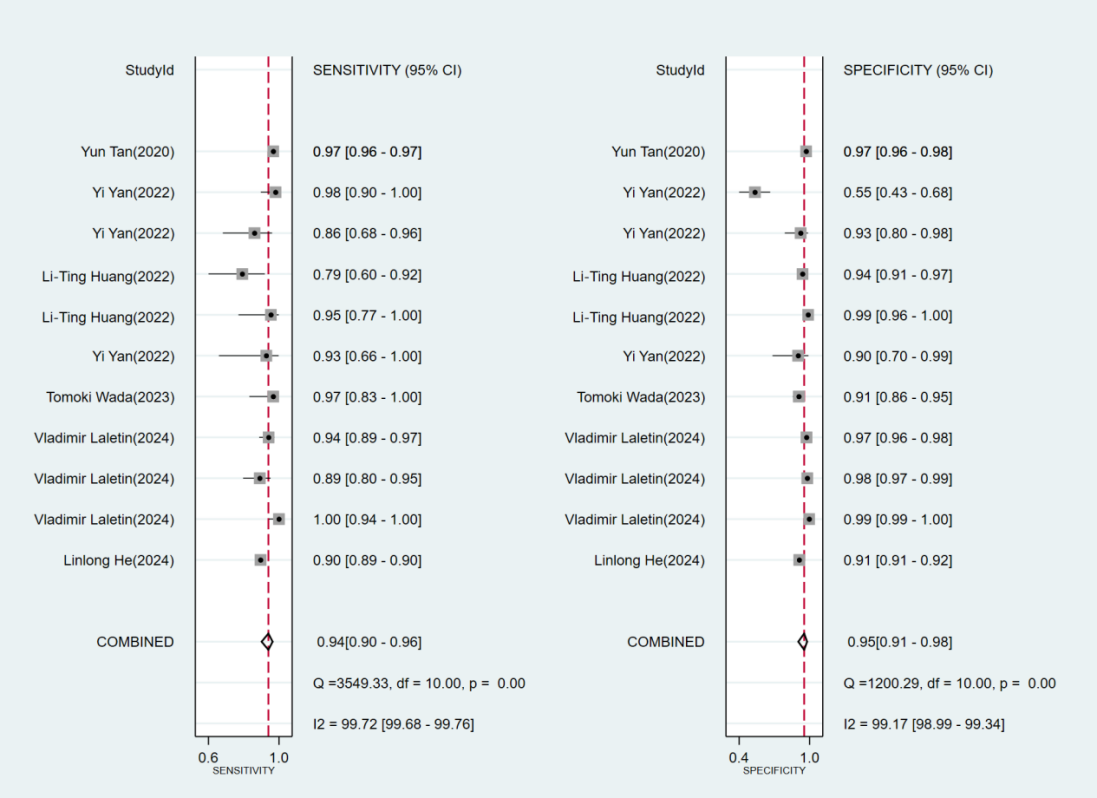


Figure S13 Forest plot for sensitivity and specialty of DL


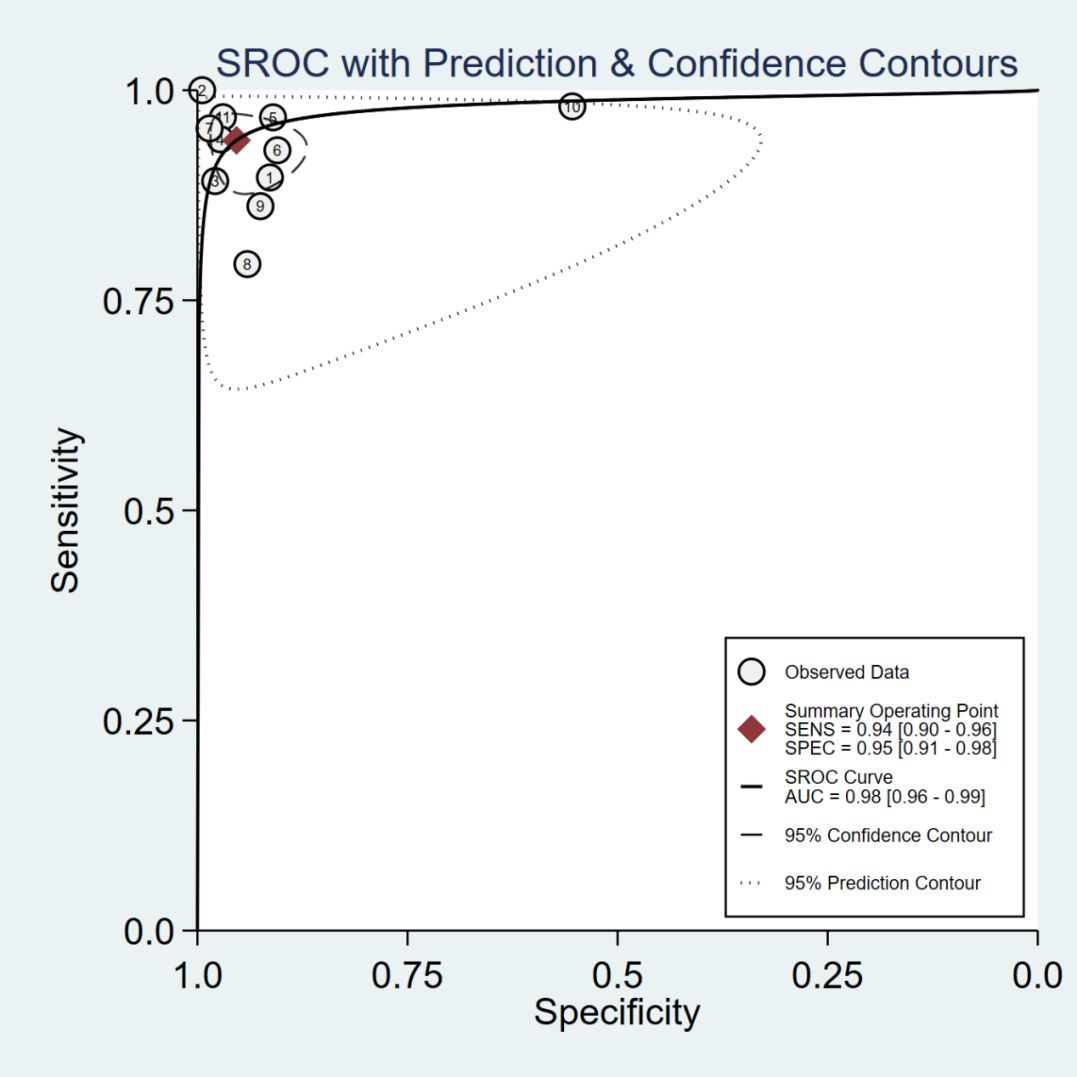


Figure S14 SROC curve for sensitivity and specialty of DL


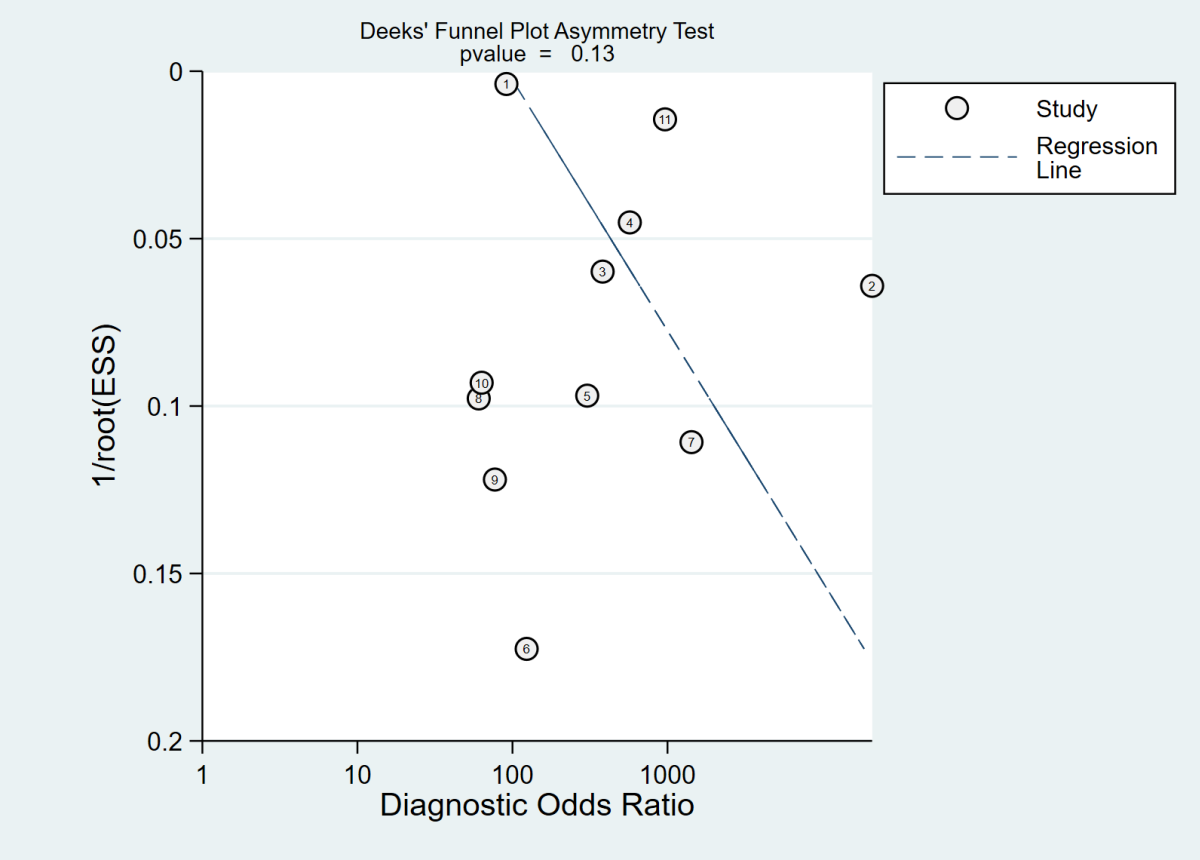


Figure S15 Deek’s funnel plot for sensitivity and specialty of DL


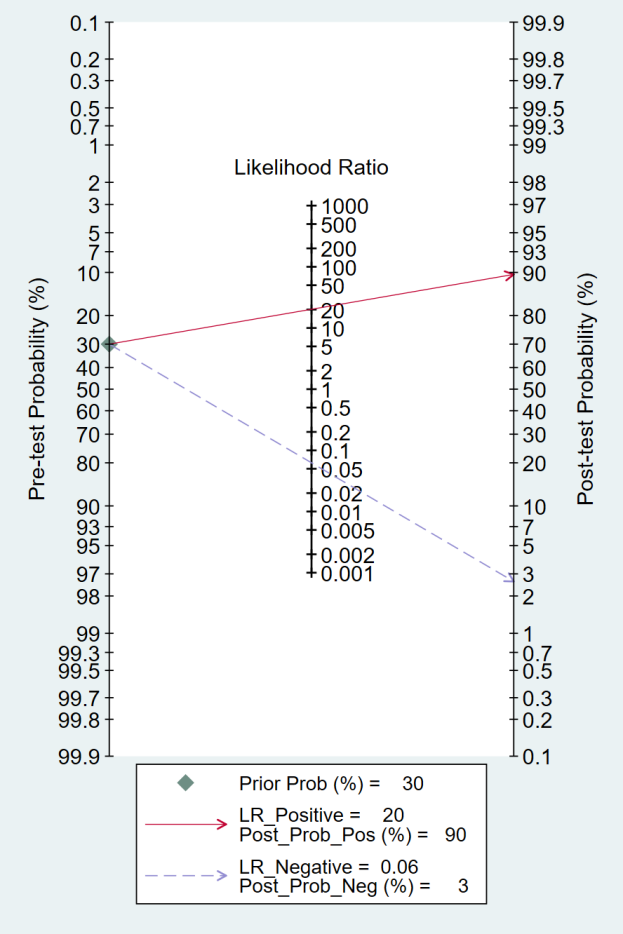


Figure S16 Fagan’s nomogram for sensitivity and specialty of DL


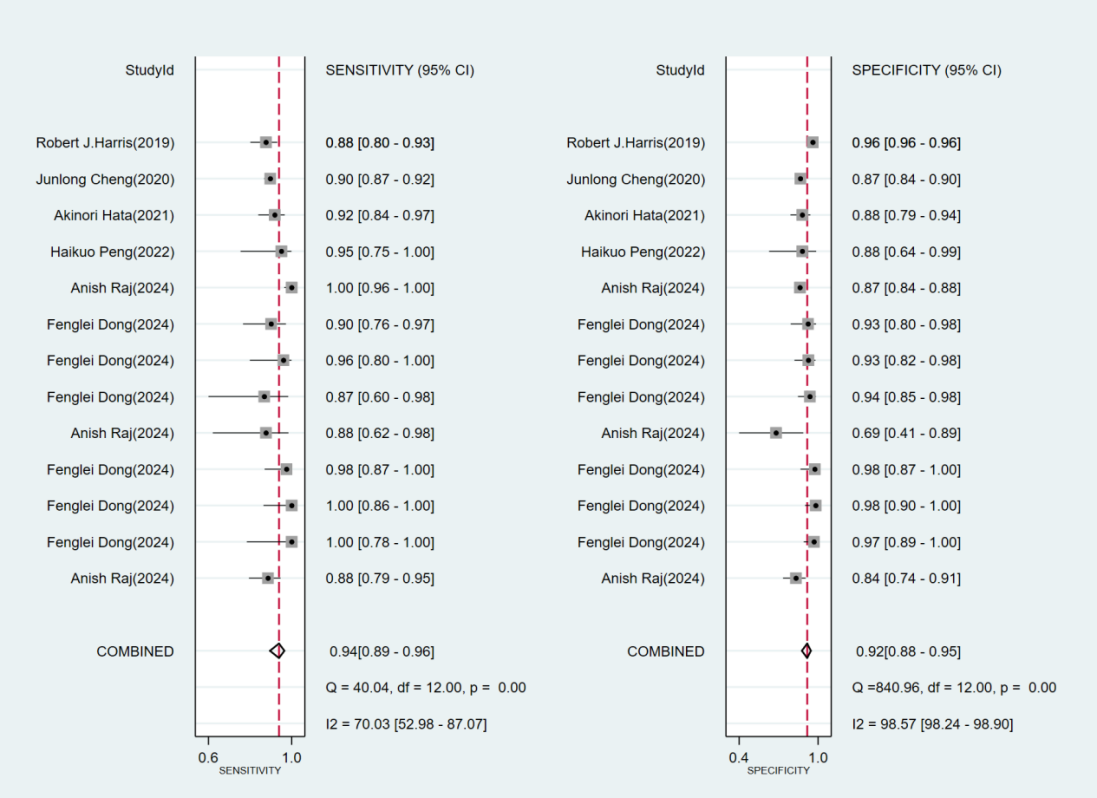


Figure S17 Forest plot for sensitivity and specialty of DL


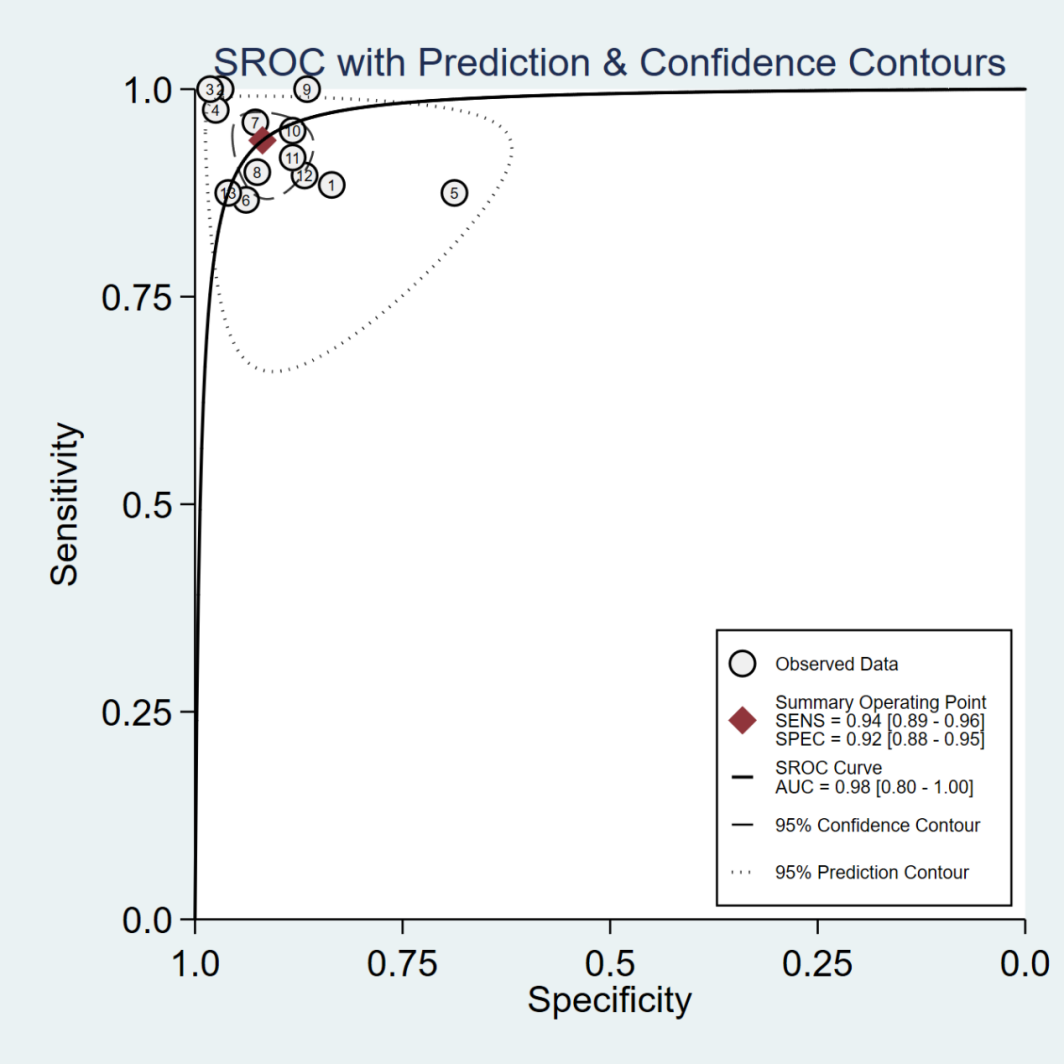


Figure S18 SROC curve for sensitivity and specialty of DL


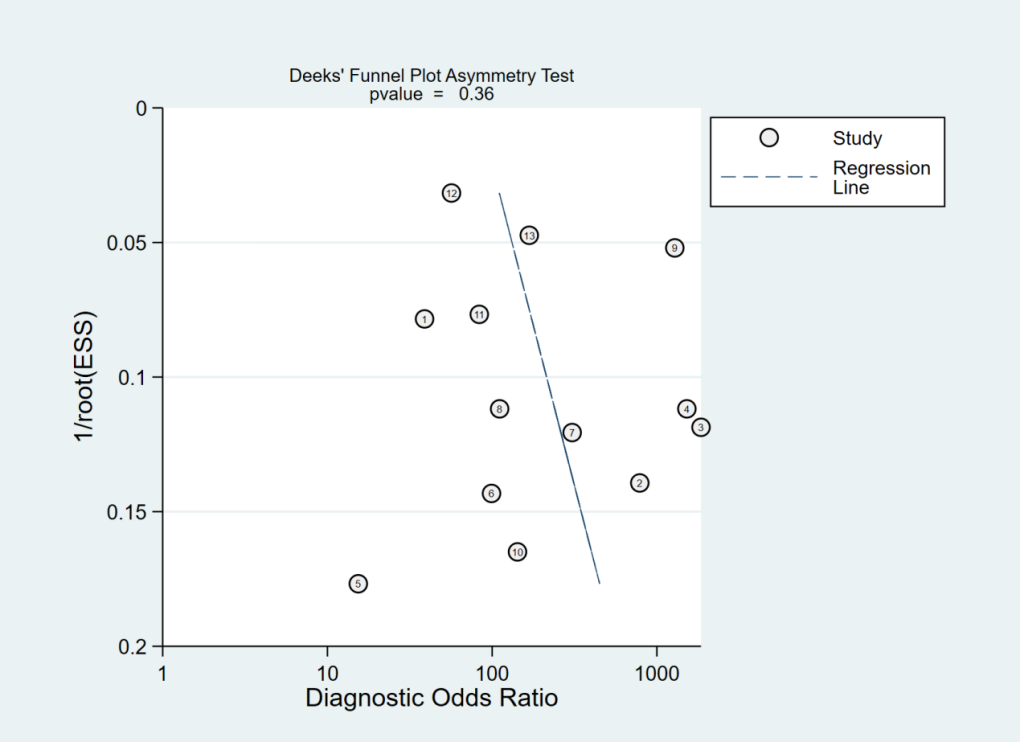


Figure S19 Deek’s funnel plot for sensitivity and specialty of DL


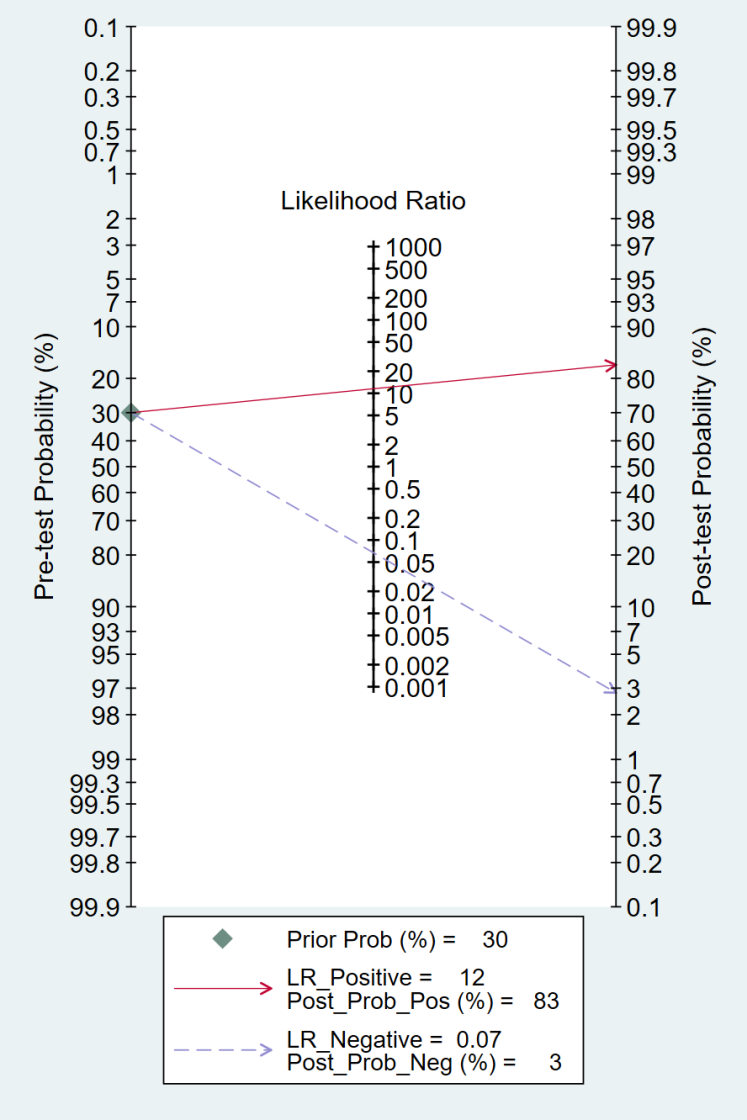


Figure S20 Fagan’s nomogram for sensitivity and specialty of DL


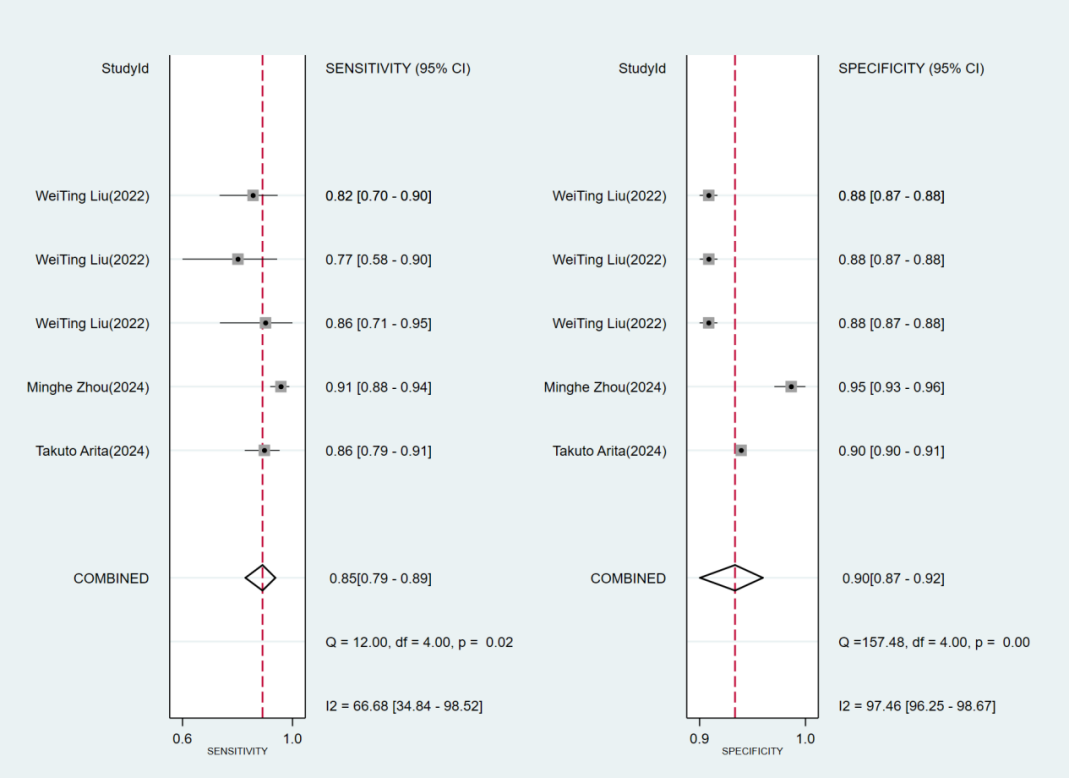


Figure S21 Forest plot for sensitivity and specialty of DL


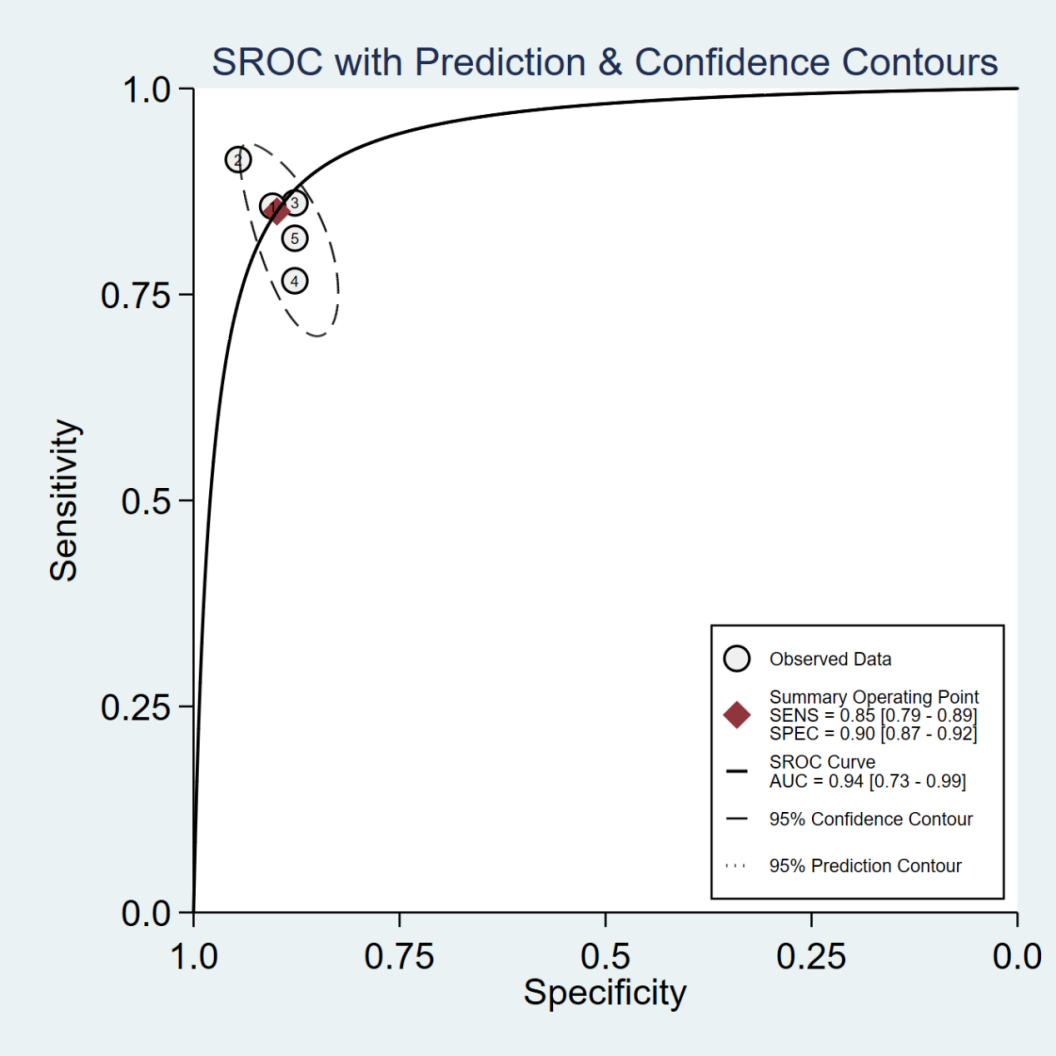


Figure S22 SROC curve for sensitivity and specialty of DL


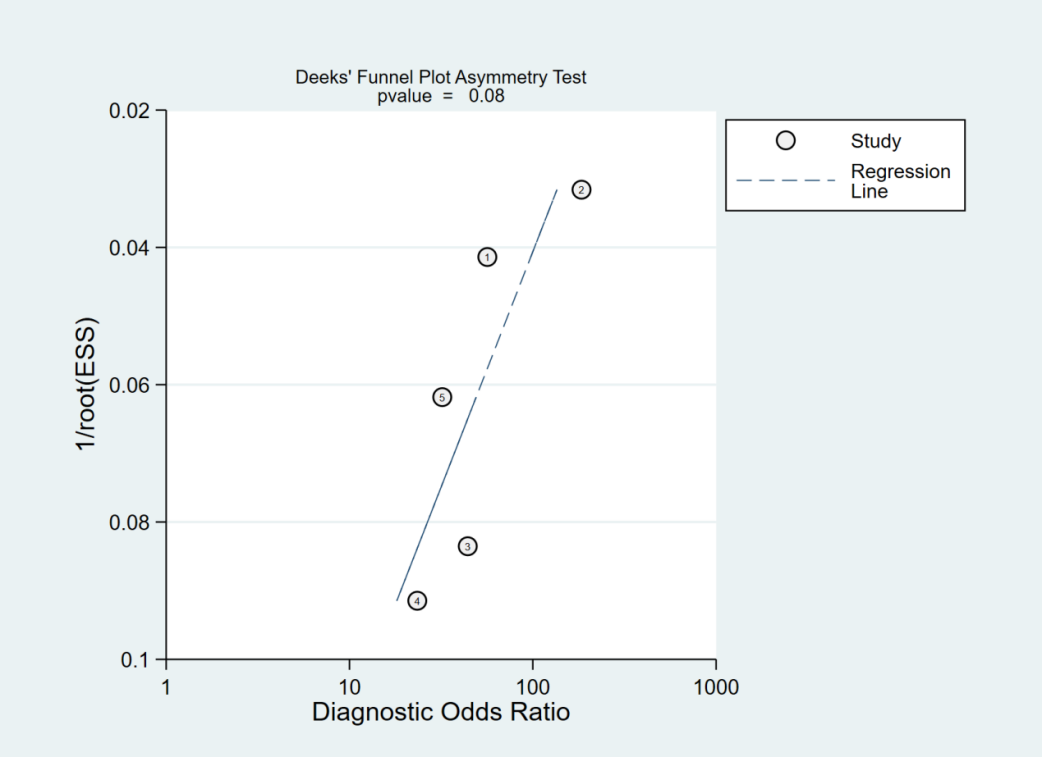


Figure S23 Deek’s funnel plot for sensitivity and specialty of DL


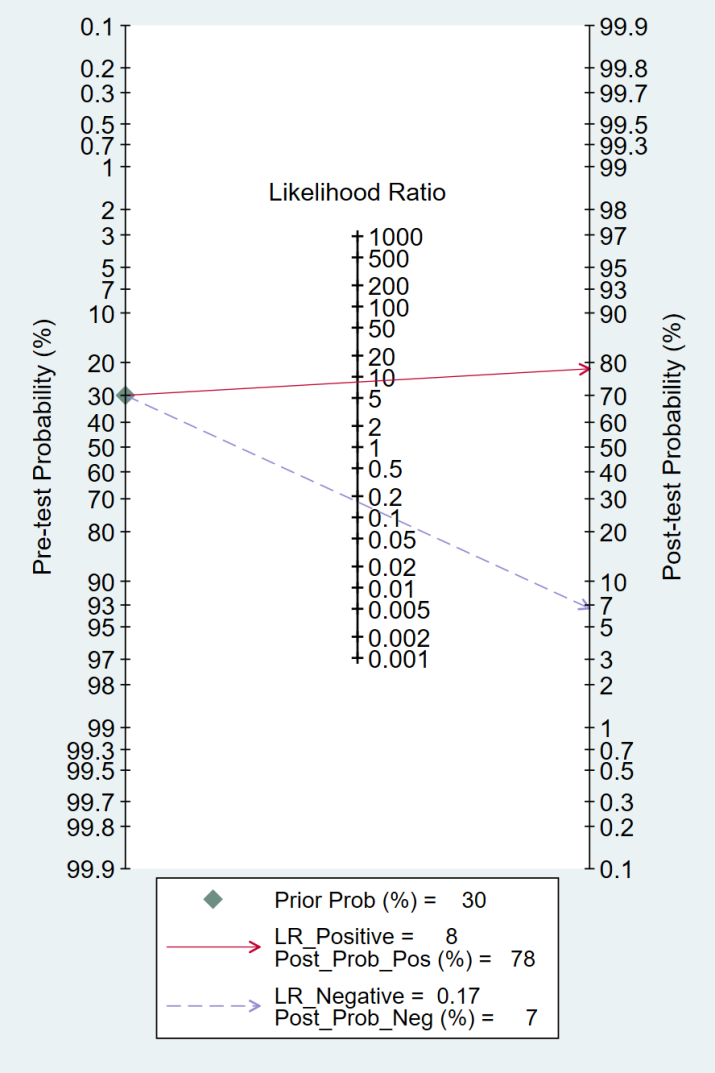


Figure S24 Fagan’s nomogram for sensitivity and specialty of DL


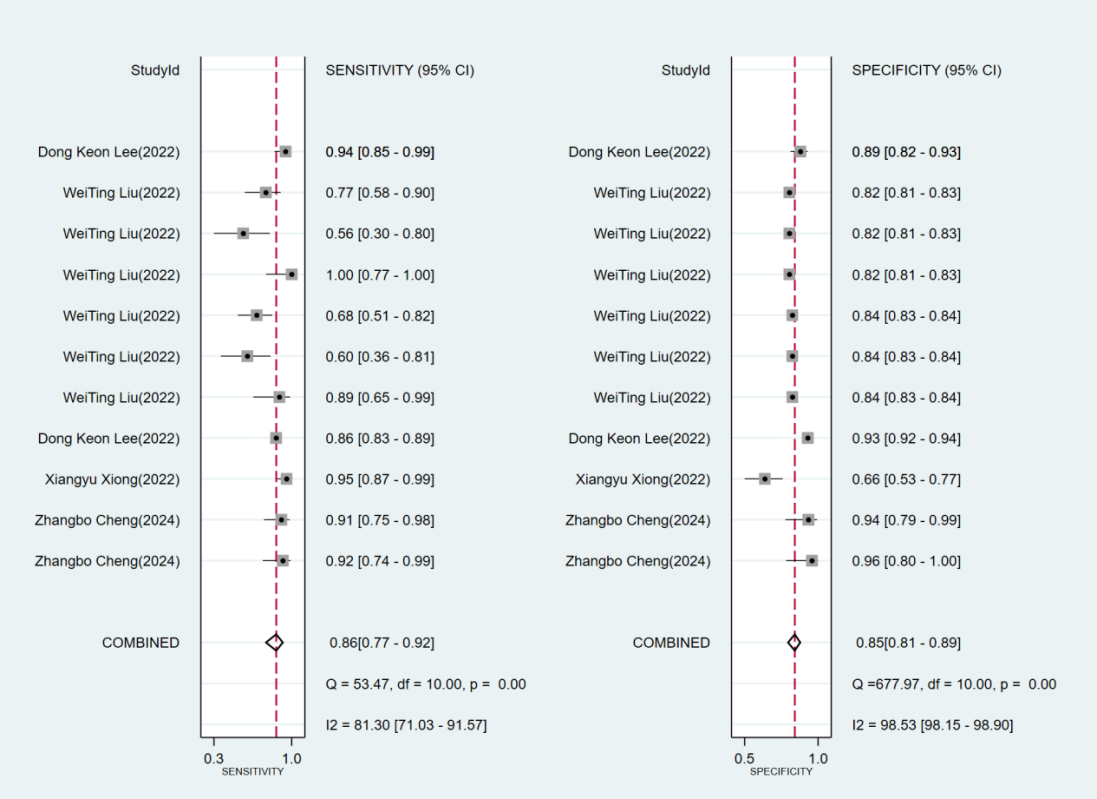


Figure S25 Forest plot for sensitivity and specialty of DL


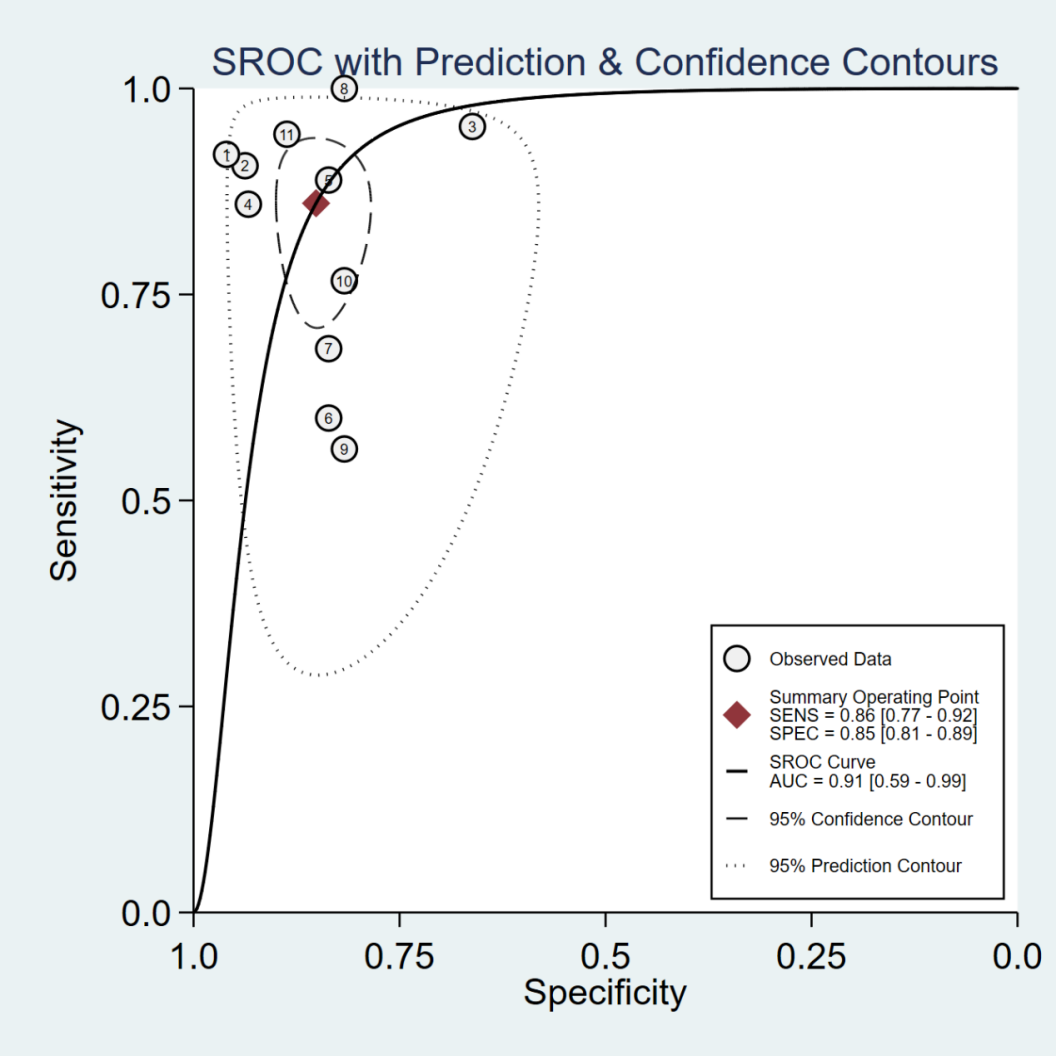


Figure S26 SROC curve for sensitivity and specialty of DL


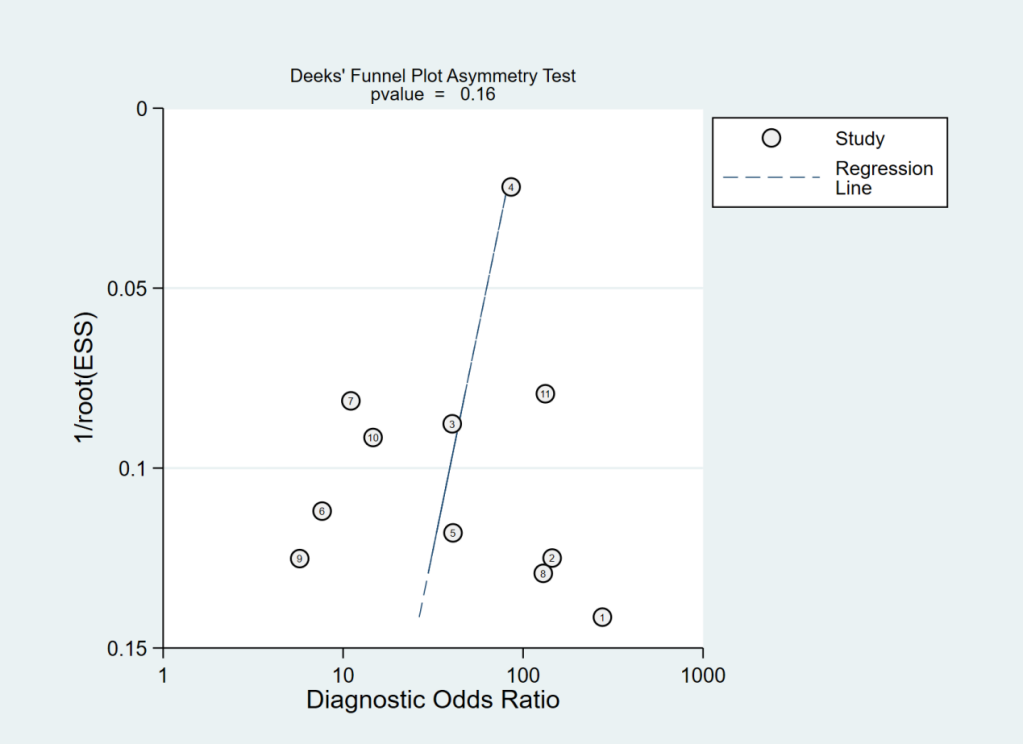


Figure S27 Deek’s funnel plot for sensitivity and specialty of DL


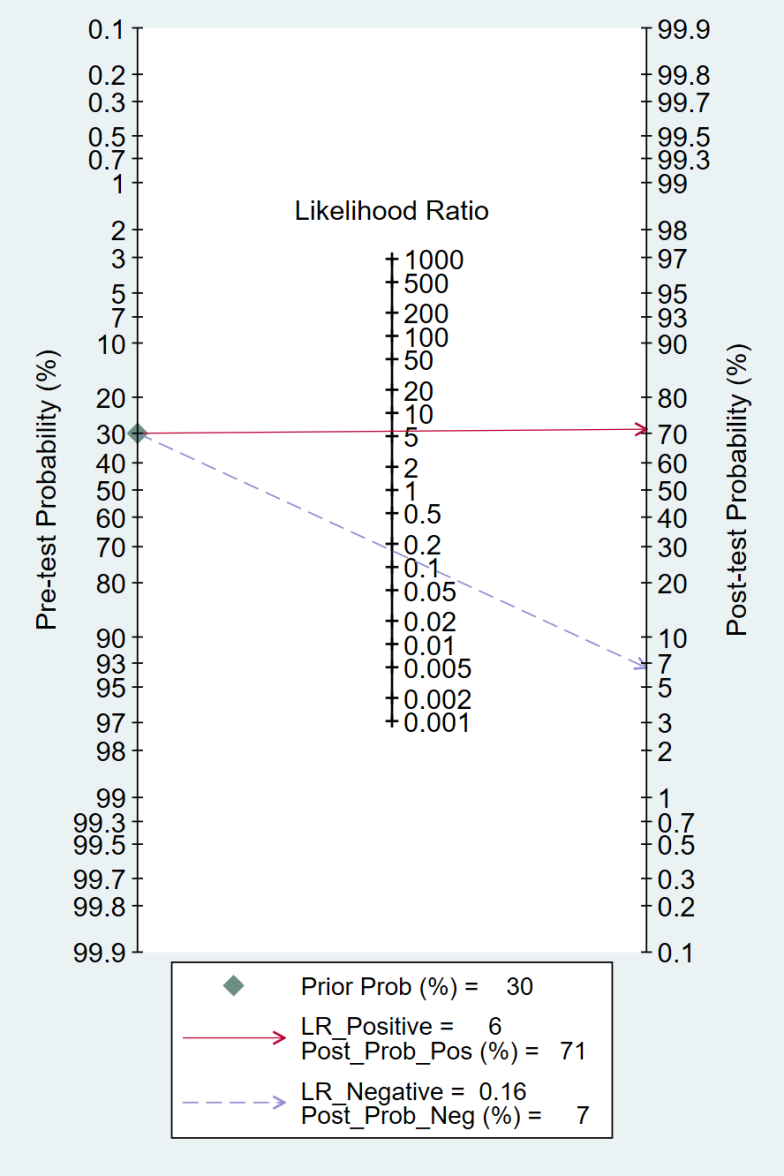


Figure S28 Fagan’s nomogram for sensitivity and specialty of DL


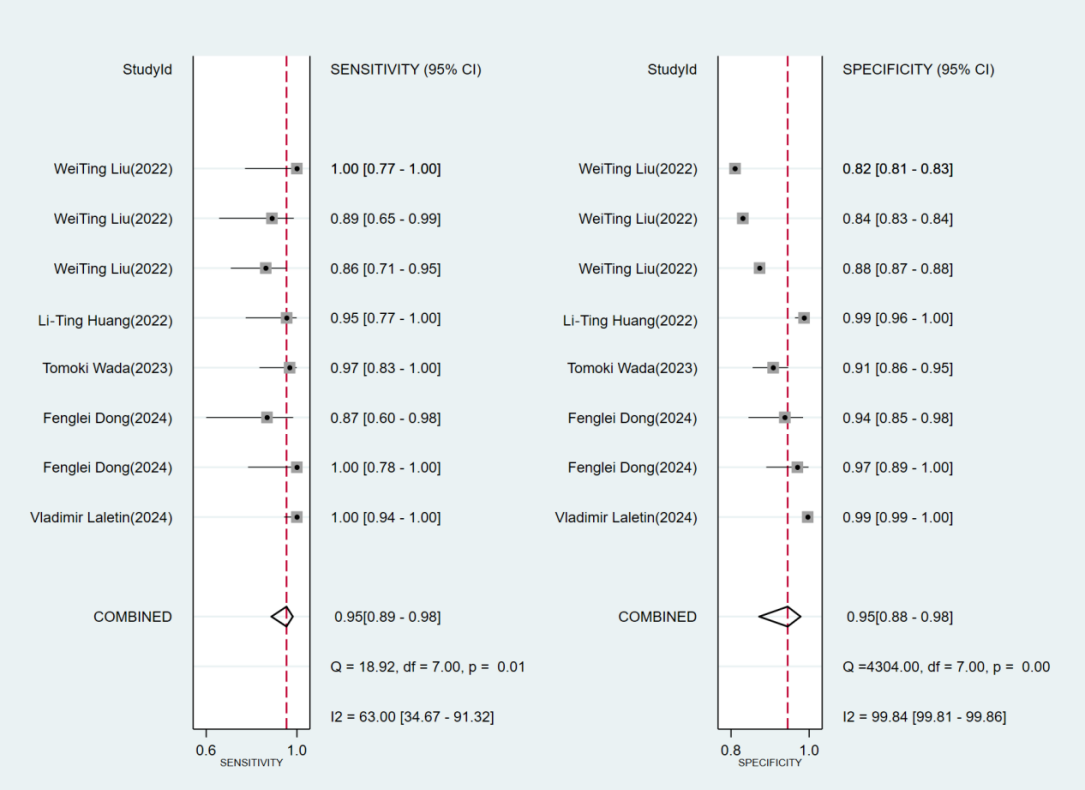


Figure S29 Forest plot for sensitivity and specialty of DL


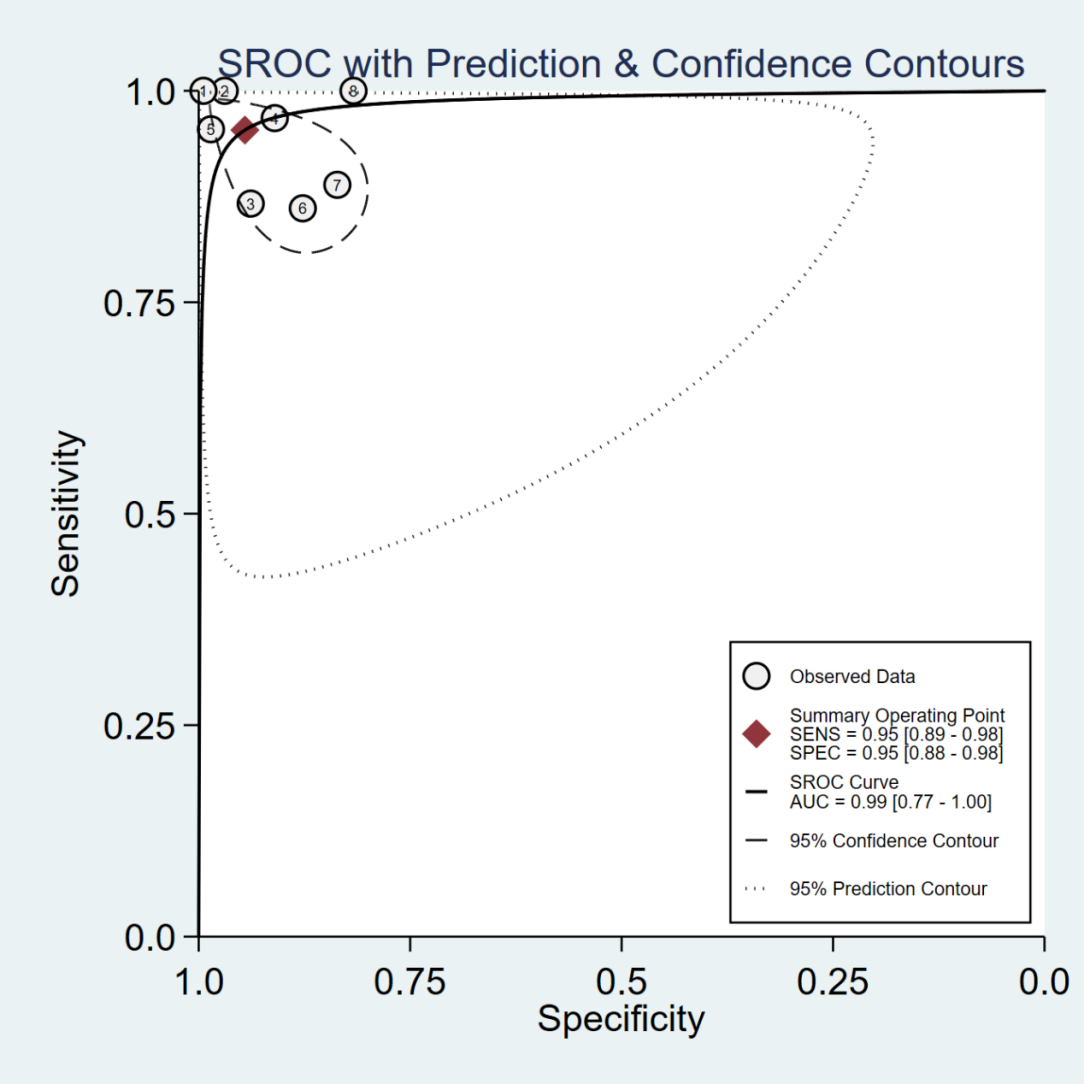


Figure S30 SROC curve for sensitivity and specialty of DL
