# Supplementary material for: Performance of image-based deep learning models for aortic dissection segmentation and diagnosis: a systematic review and meta-analysis
Source: Front Cardiovasc Med. 2026 Apr 14;13:1734208. doi: 10.3389/fcvm.2026.1734208 (PMC13121068; doi:10.3389/fcvm.2026.1734208)
Supplement: Supplementary file 6 [file Supplementaryfile3.docx]

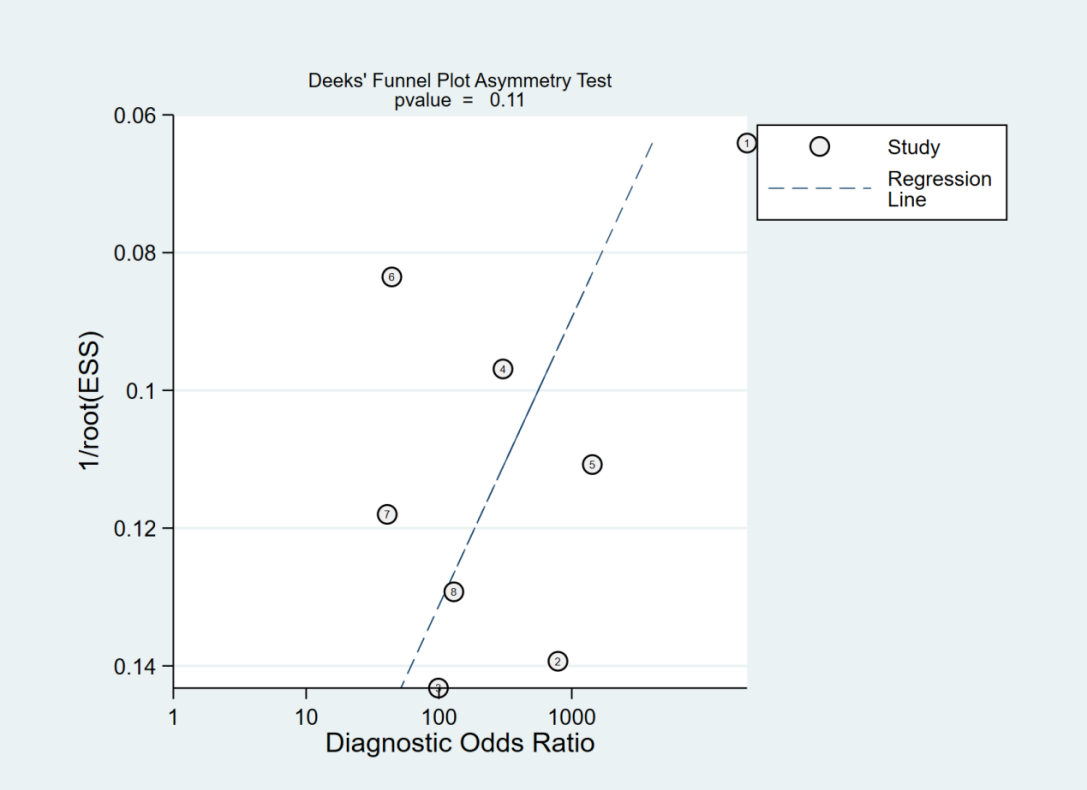


Figure S31 Deek’s funnel plot for sensitivity and specialty of DL


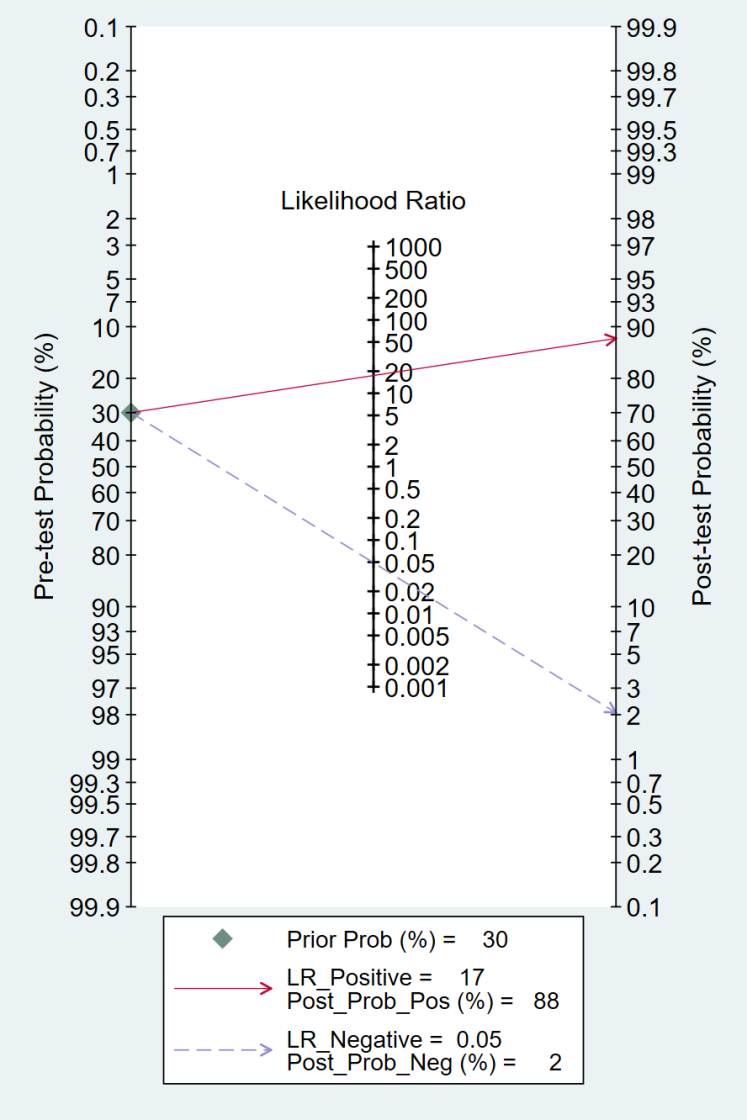


Figure S32 Fagan’s nomogram for sensitivity and specialty of DL


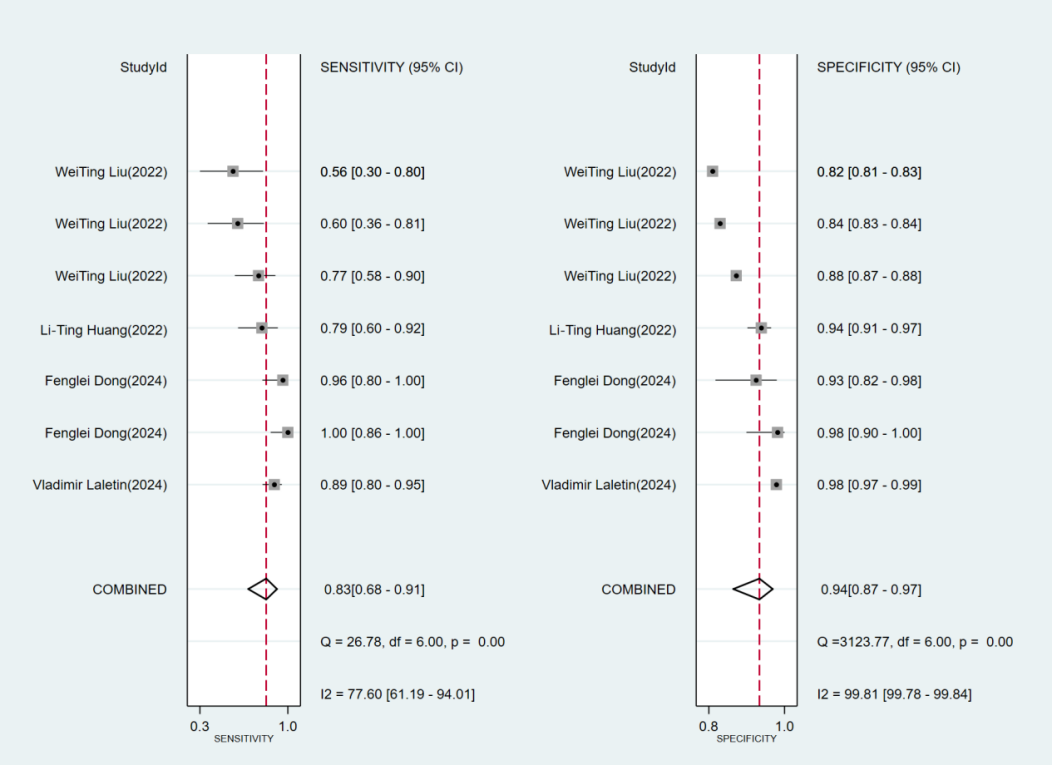


Figure S33 Forest plot for sensitivity and specialty of DL


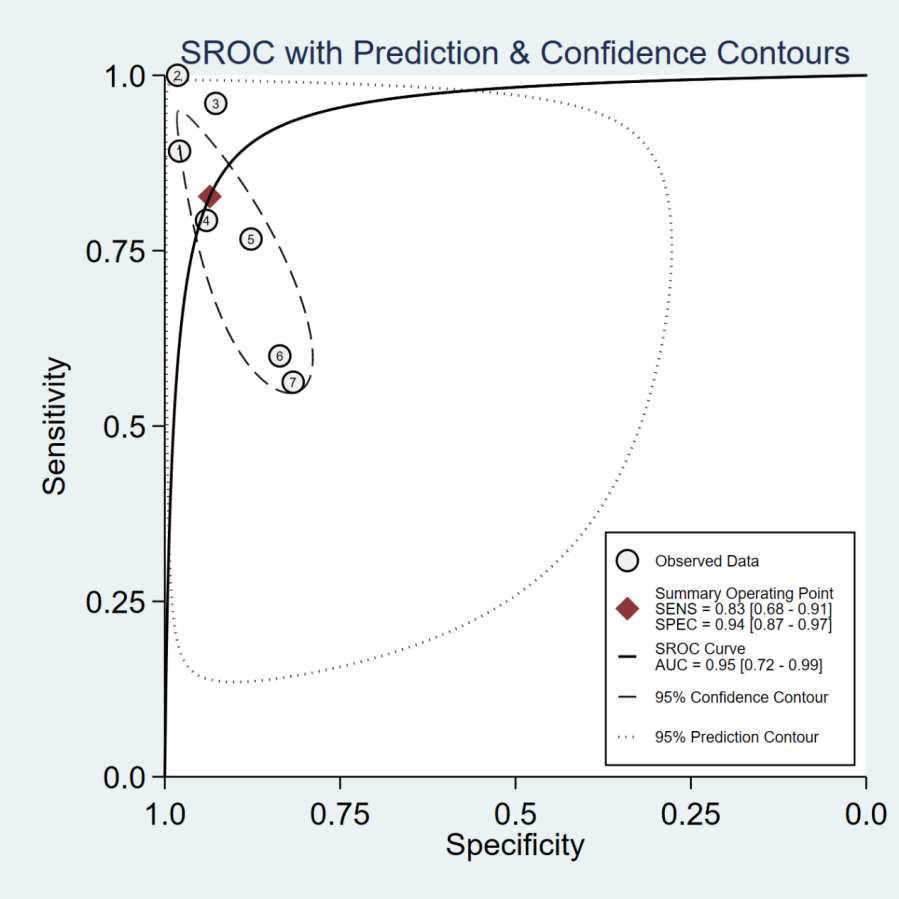


Figure S34 SROC curve for sensitivity and specialty of DL


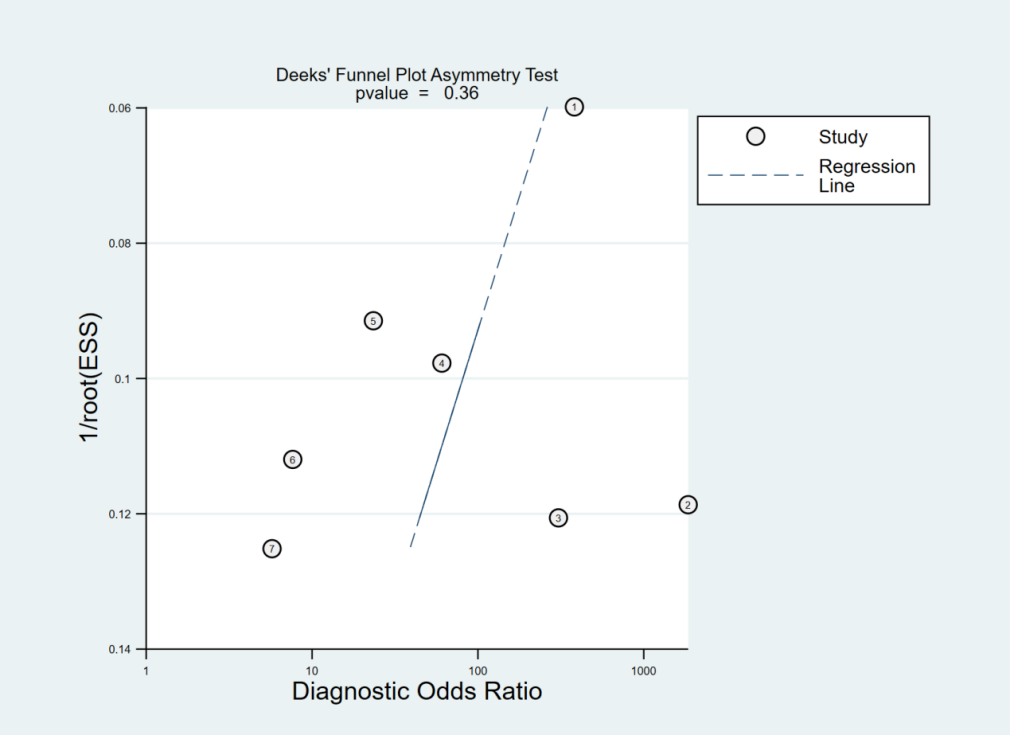


Figure S35 Deek’s funnel plot for sensitivity and specialty of DL


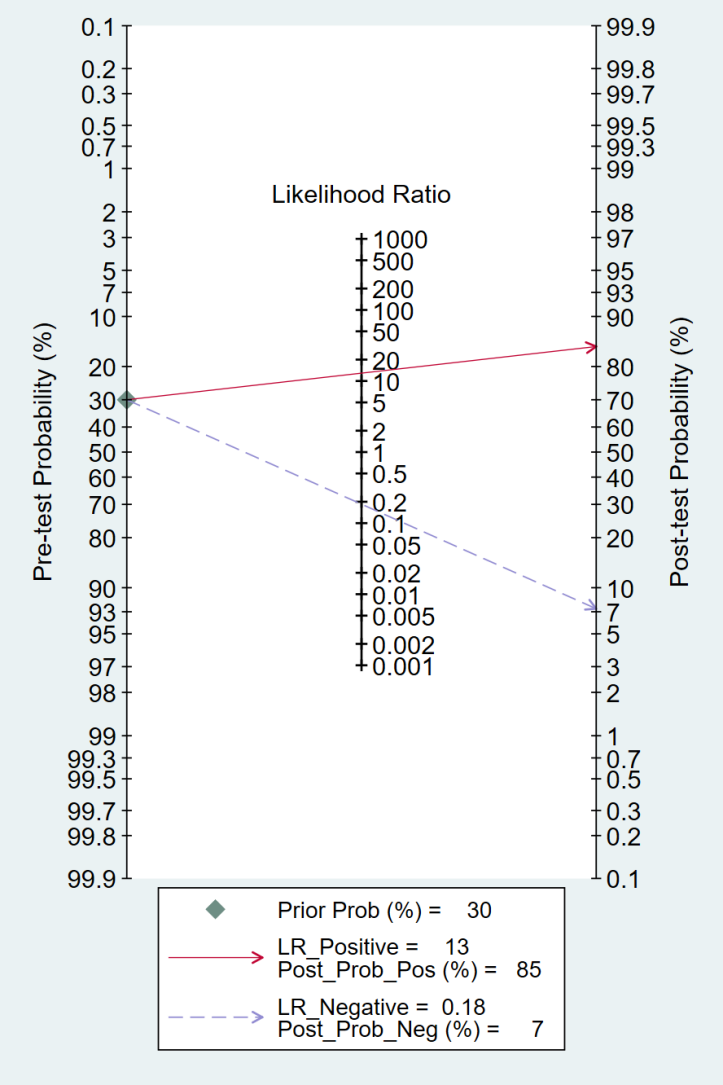


Figure S36 Fagan’s nomogram for sensitivity and specialty of DL


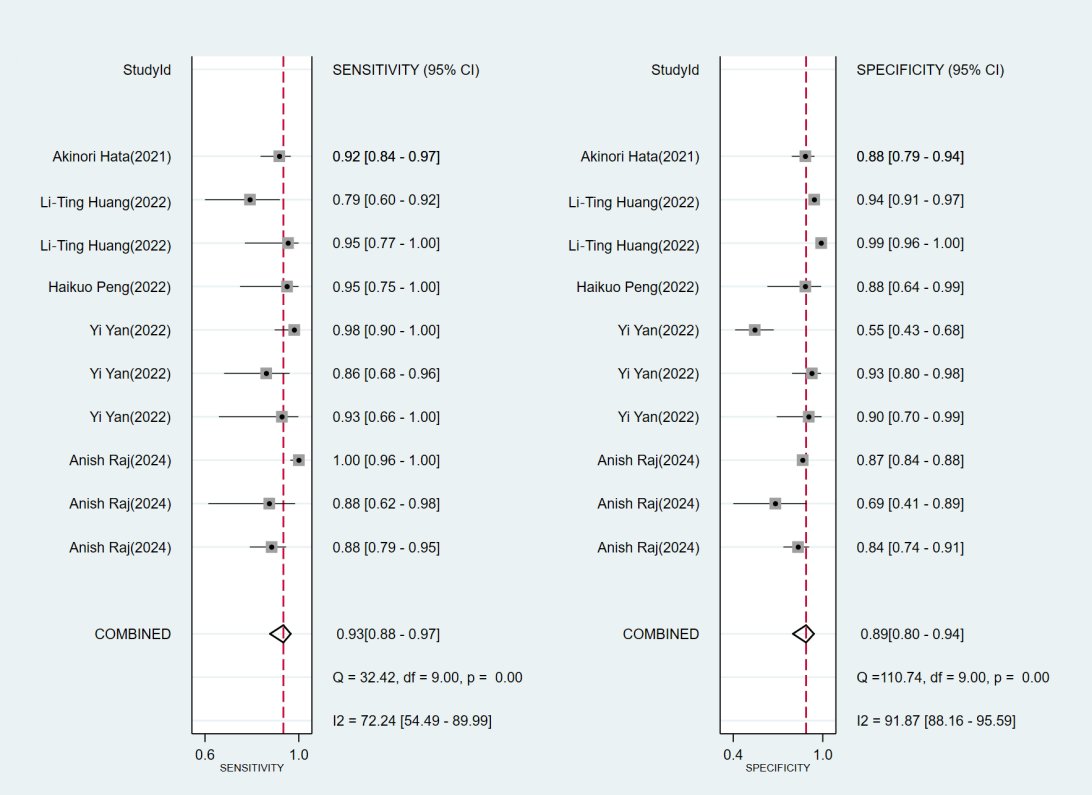


Figure S37 Forest plot for sensitivity and specialty of DL


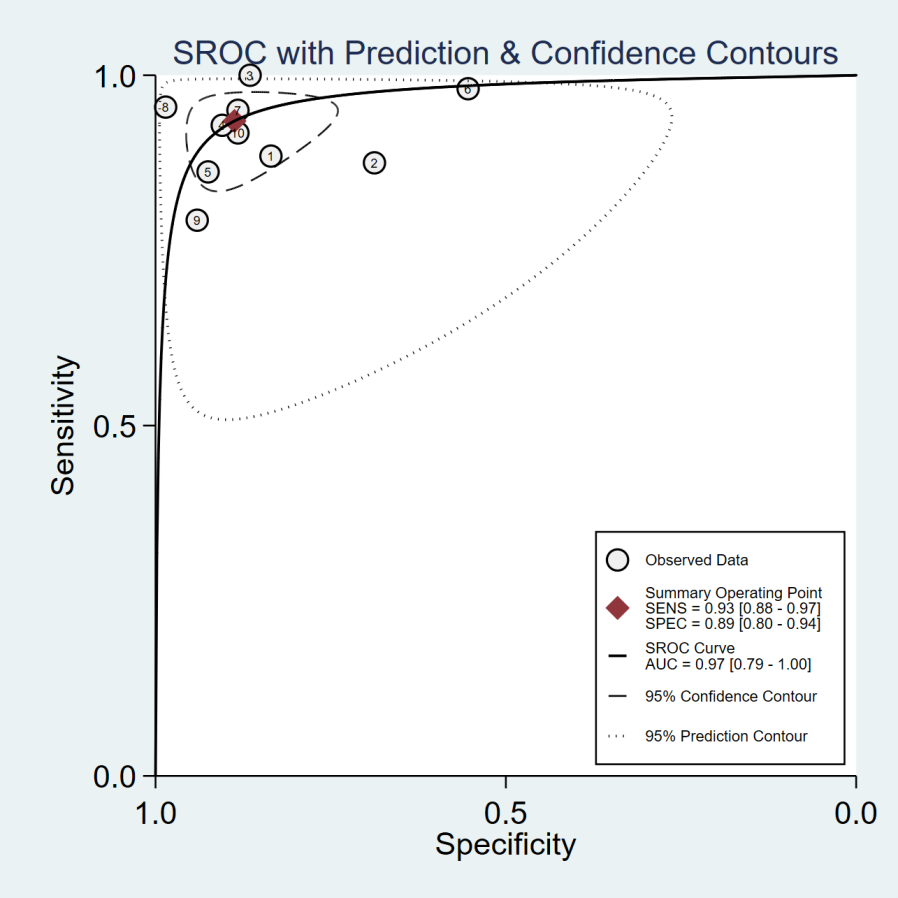


Figure S38 SROC curve for sensitivity and specialty of DL


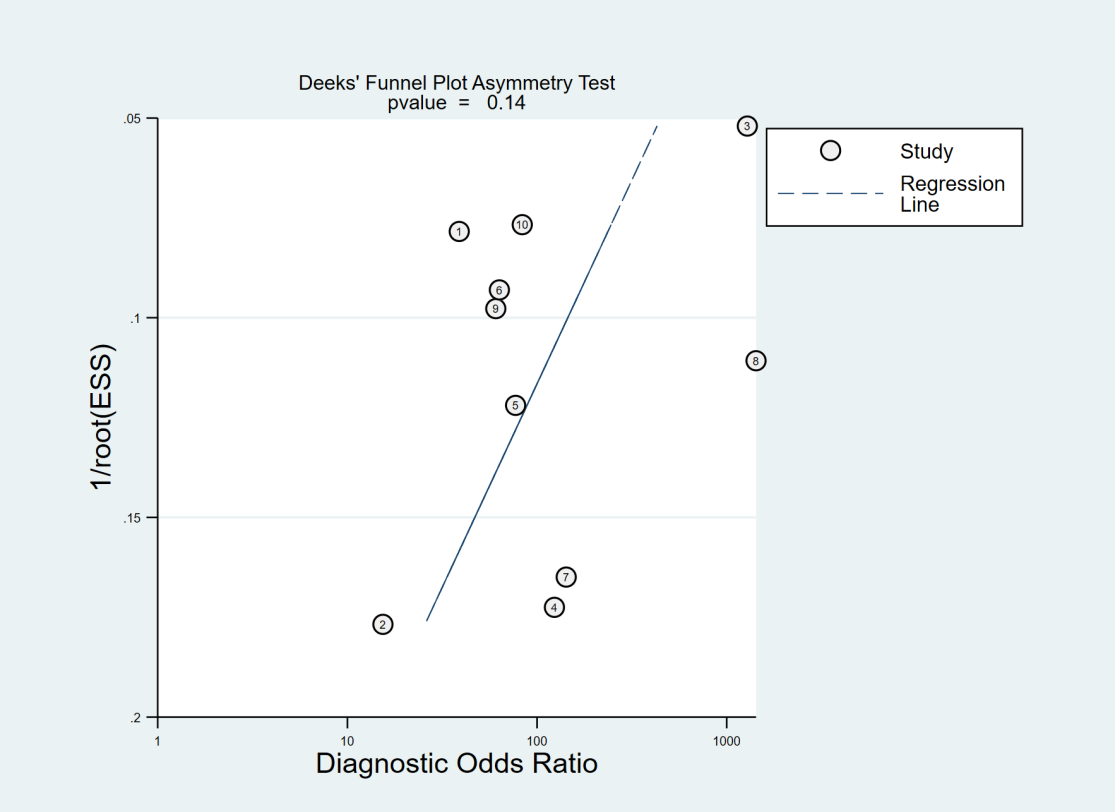


Figure S39 Deek’s funnel plot for sensitivity and specialty of DL


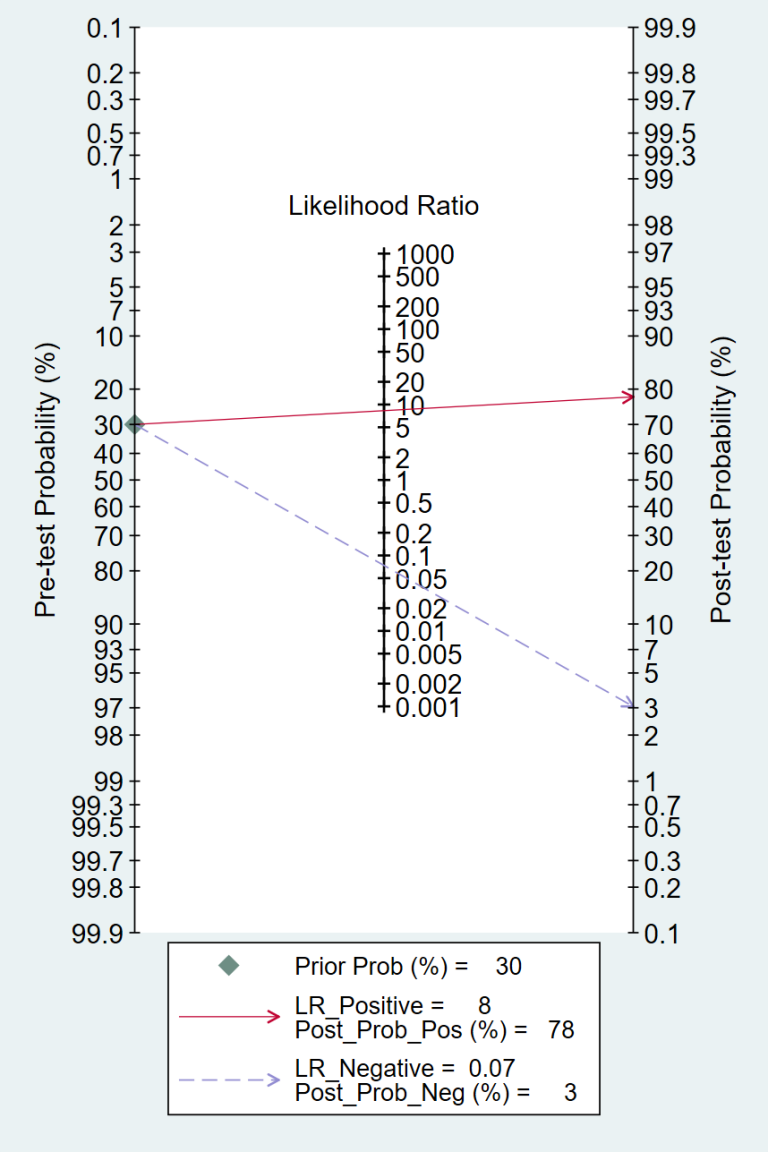


Figure S40 Fagan’s nomogram for sensitivity and specialty of DL


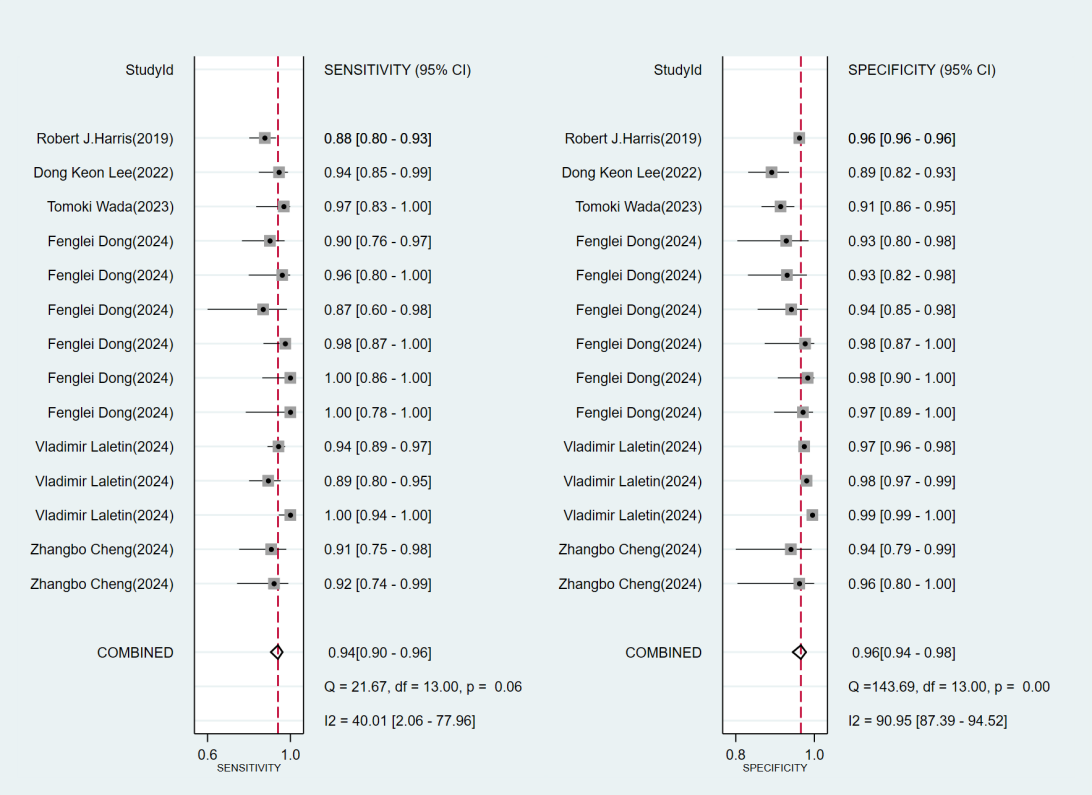


Figure S41 Forest plot for sensitivity and specialty of DL


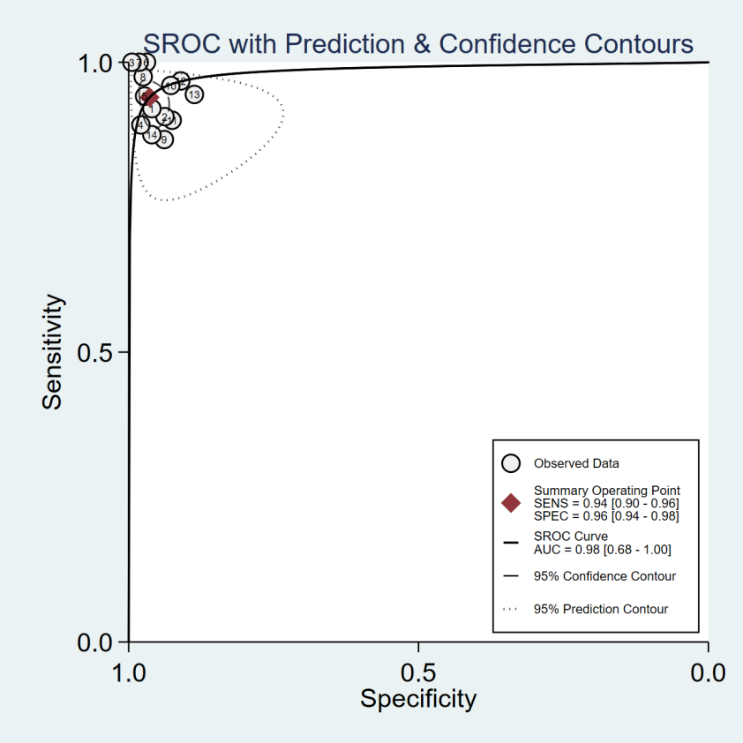


Figure S42 SROC curve for sensitivity and specialty of DL


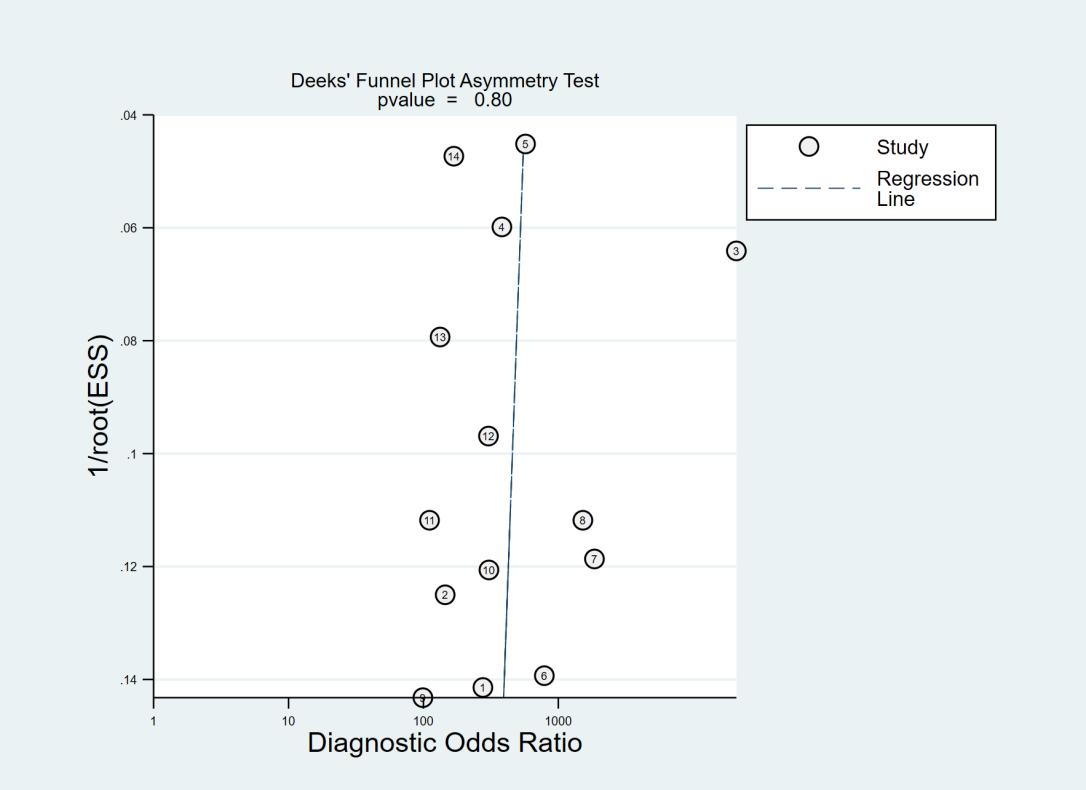


Figure S43 Deek’s funnel plot for sensitivity and specialty of DL


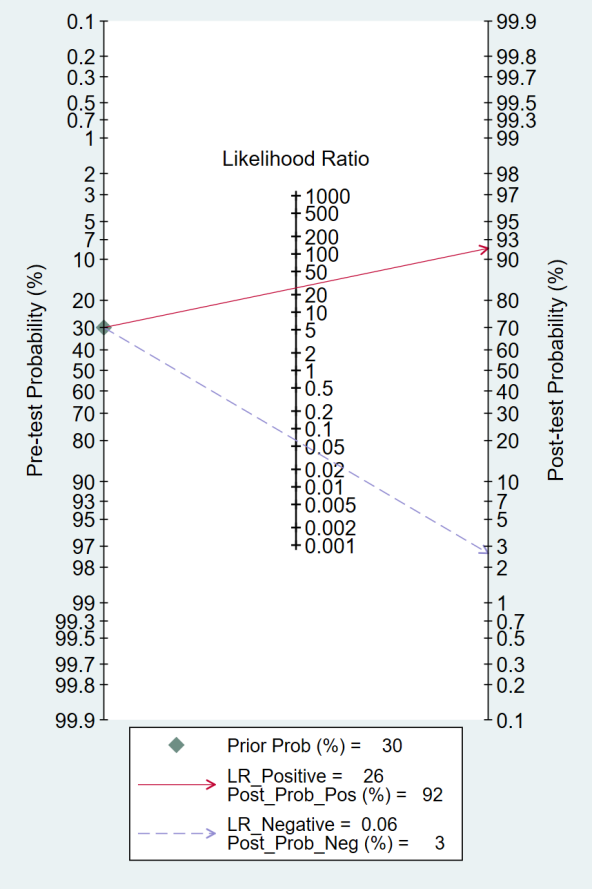


Figure S44 Fagan’s nomogram for sensitivity and specialty of DL


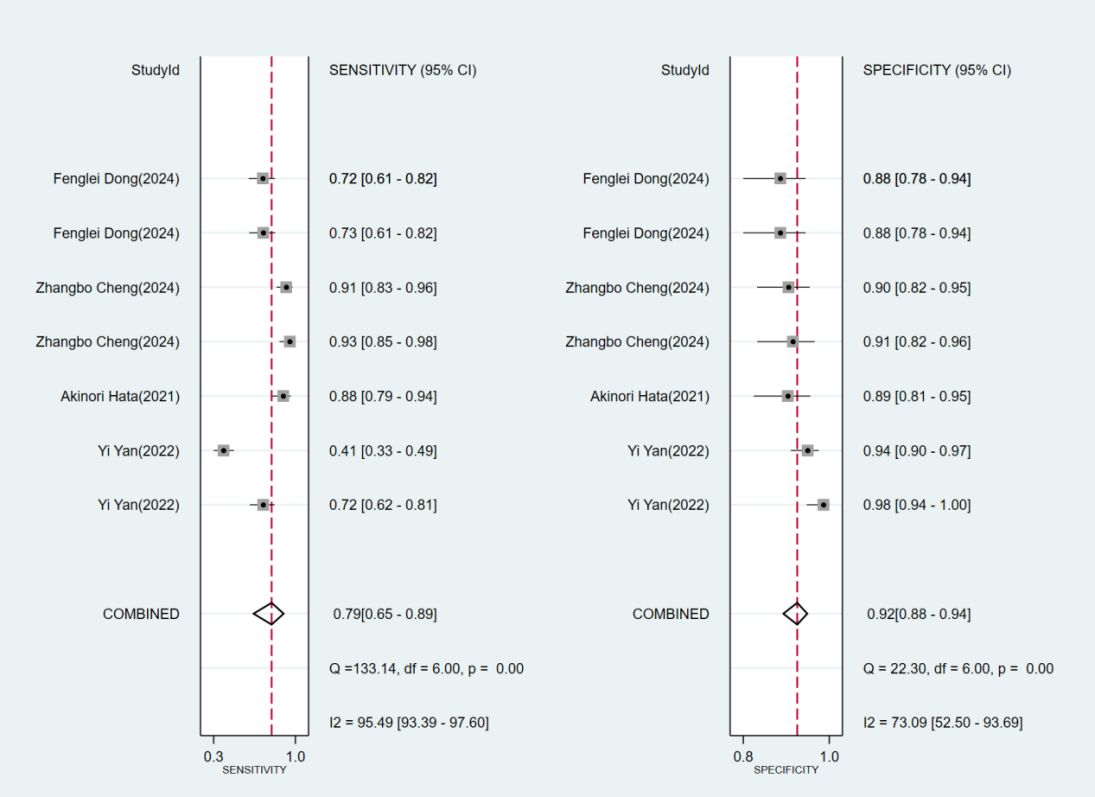


Figure S45 Forest plot for sensitivity and specialty of clinicians


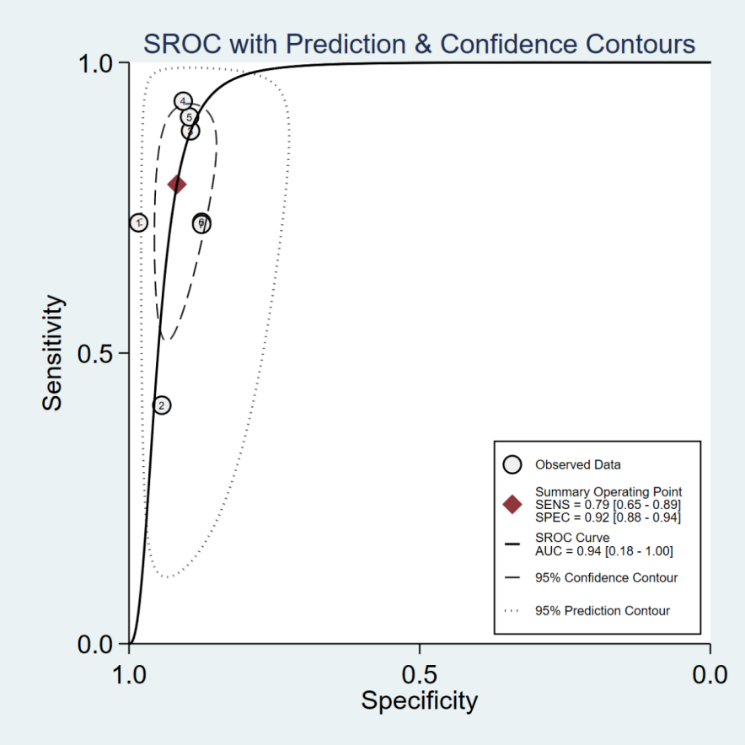


Figure S46 SROC curve for sensitivity and specialty of clinicians


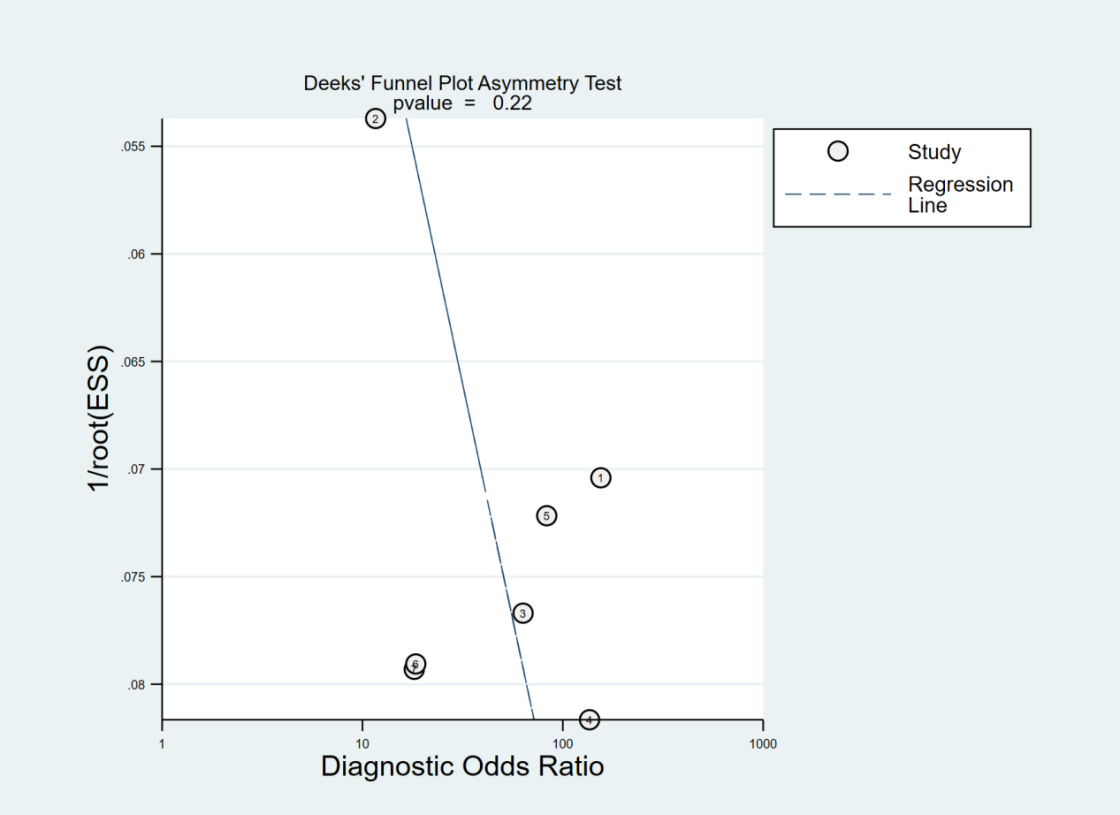


Figure S47 Deek’s funnel plot for sensitivity and specialty of clinicians


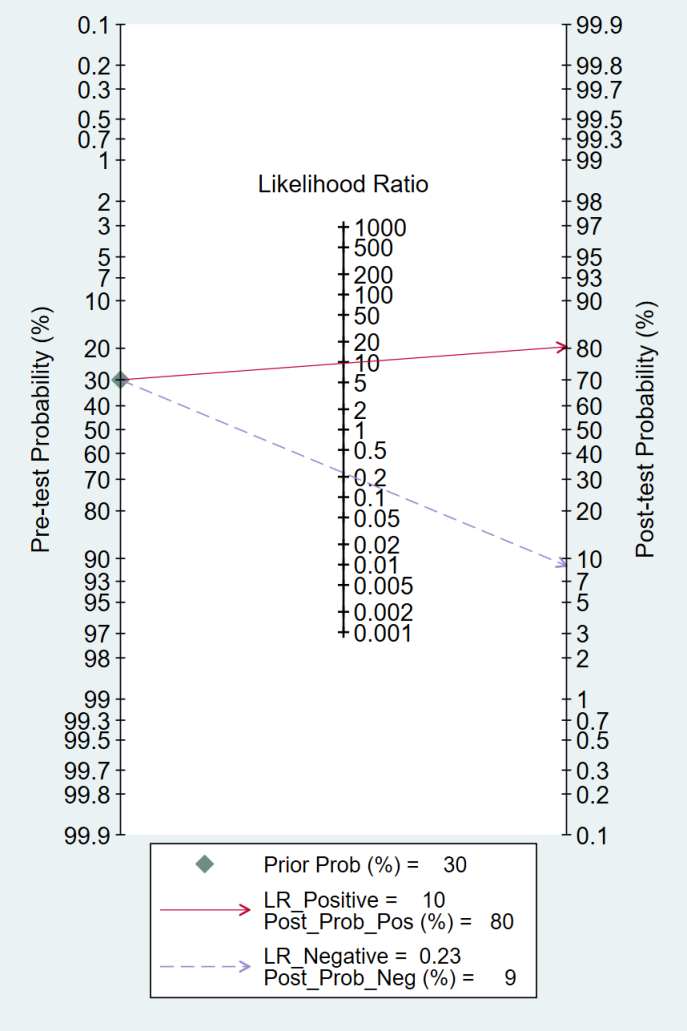


Figure S48 Fagan’s nomogram for sensitivity and specialty of clinicians


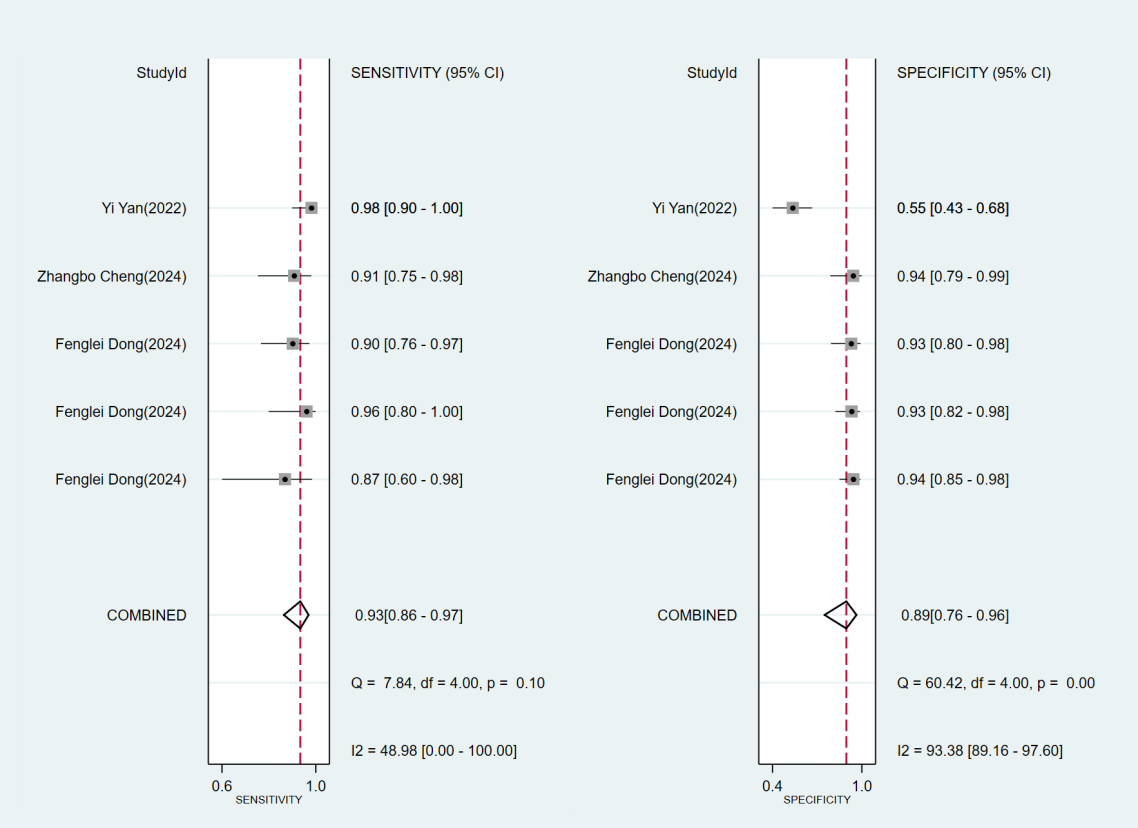


Figure S49 Forest plot for sensitivity and specialty of DL


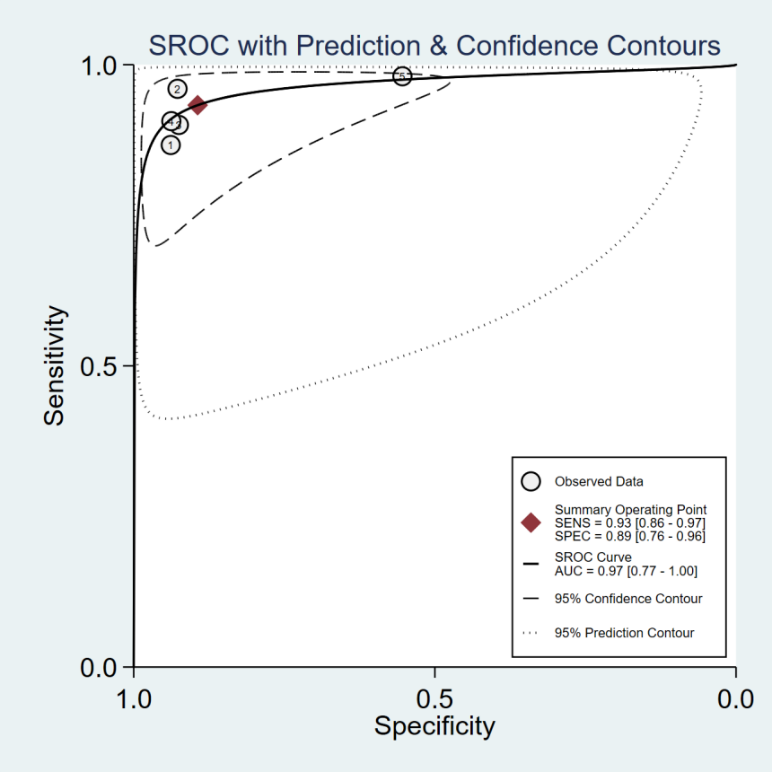


Figure S50 SROC curve for sensitivity and specialty of DL
